# Highly Enantioselective Lewis Acid Catalyzed Conjugate Addition of Imidazo[1,2-*a*]pyridines to α,β-Unsaturated 2-Acylimidazoles under Mild Conditions

**DOI:** 10.1021/acs.joc.4c00445

**Published:** 2024-06-06

**Authors:** Maria Eduarda C. Thedy, Vanessa Pereira, Caio Rodrigo dos Santos, Luiz Paulo A. Belli, Marcelo S. Franco, Adailton J. Bortoluzzi, Louis P. Sandjo, Antonio L. Braga, Francisco F. de Assis

**Affiliations:** Department of Chemistry, Universidade Federal de Santa Catarina, Florianópolis, Santa Catarina 88040-900, Brazil

## Abstract

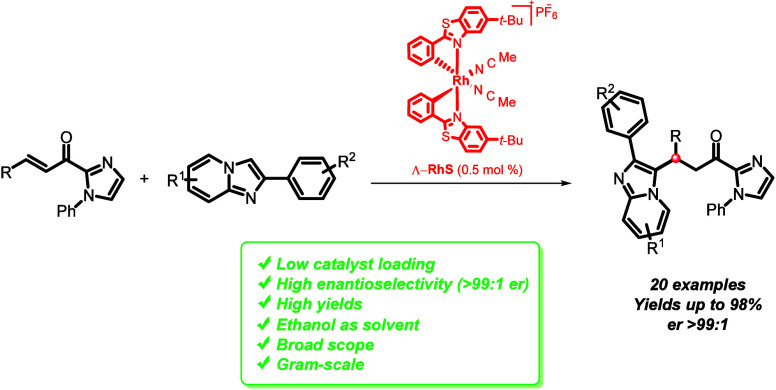

A highly enantioselective protocol for the conjugate addition of 2-arylimidazo[1,2-*a*]pyridines and other imidazo derivatives to α,β-unsaturated 2-acylimidazoles is described. The method uses a previously reported chiral-at-metal rhodium catalyst and provides the corresponding adducts in yields of 25–98% with enantioselectivities up to er > 99:1. Additionally, the transformation proceeds under mild conditions using ethanol as the solvent at room temperature.

Imidazoles and imidazopyridines have been considered privileged scaffolds as these skeletons represent the structural core of a large range of drugs and bioactive compounds^1−3^ (Figure 1). Meanwhile, imidazo[1,2-*a*]pyridines derivatives have received remarkable attention from the scientific community owing to a great number of biological activities associated–antimicrobial,^4^ antiviral,^5^ antitumor,^6^ and antiparasitic^7^ (Figure 1). The imidazo[1,2-*a*]pyridine moiety is embedded in the structure of many commercial drugs such as the anxiolytics zolpidem, alpidem, and saripidem and anti-inflammatory miroprofen. Moreover, this heterocyclic aromatic system because of its electronic density and reactivity, offers the possibility to construct complex molecules^8^ and materials.^9^ Based on the previous information, the development of new and efficient synthetic methodologies capable of generating unprecedented structural derivatives of this privileged heterocyclic core is of great importance. The most attractive reaction pattern for the design of synthetic transformation using imidazo[1,2-*a*]pyridines is the nucleophilicity of C3. This reaction bias has been explored in recently reported synthetic methods designed to promote the functionalization of these heterocycles, including a 1,6-conjugate addition reaction of imidazopyridines to *p*-quinone methides,^10^ a Rh-catalyzed 2-fold conjugate addition of 2-arylimidazo[1,2-*a*]pyridines to *p*-quinols,^11^ and also an alkylation protocol under Lewis acid catalyzed conditions, among others^12^ (Scheme 1).

**Figure 1:**
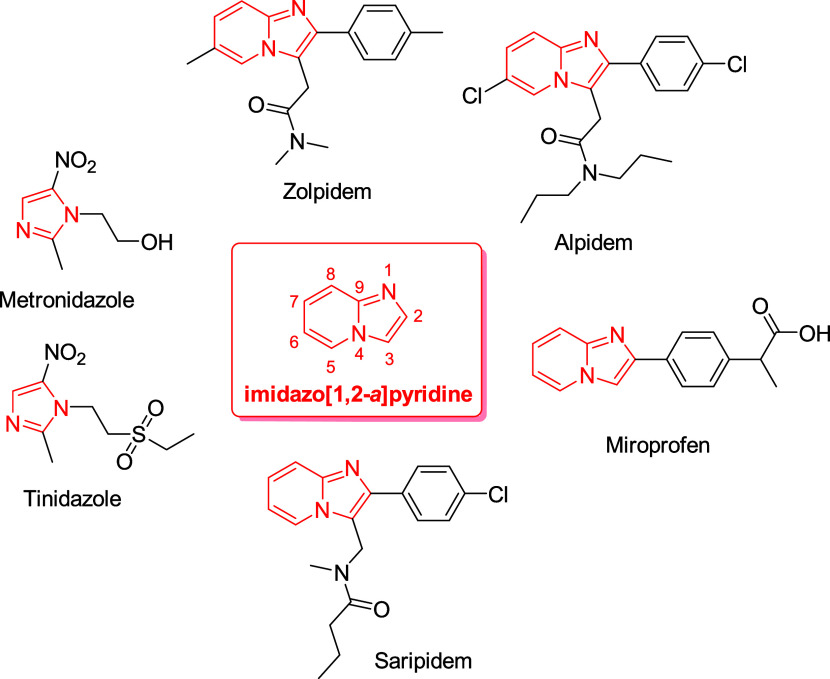
Representative pharmaceuticals containing an imidazole or imidazopyridine moiety.

**Scheme 1:**
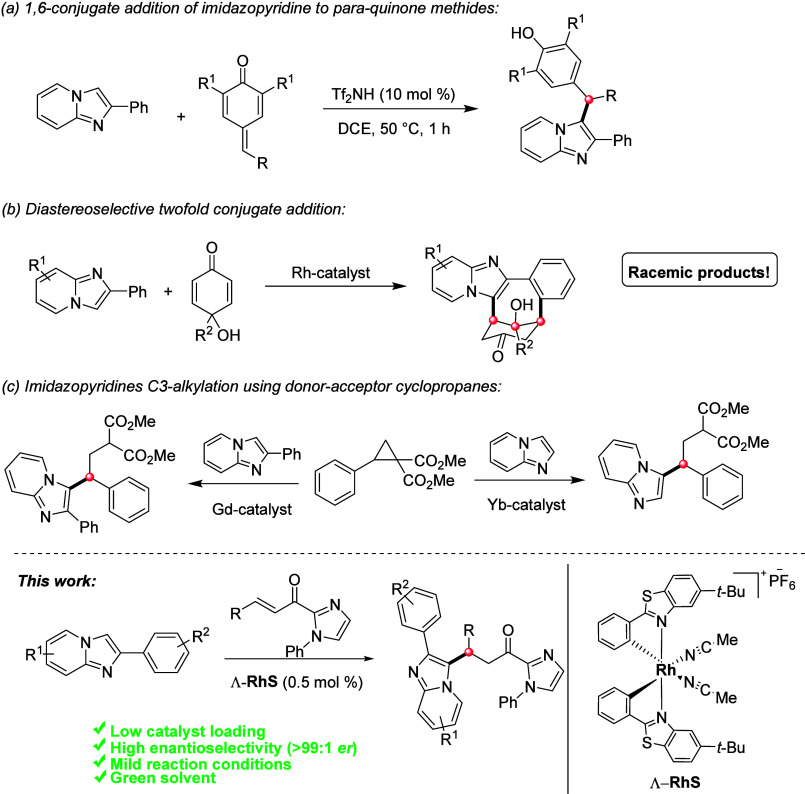
Selected Examples of Imidazo[1,2-*a*]pyridines C3-Functionalization and the Work Presented in This Study

Chirality is an intrinsic characteristic of living organisms. The human body uses only l-amino acids to produce proteins and enzymes, causing these biomacromolecules to interact differently with the enantiomers of a chiral compound, subsequently leading to different biological responses.

Despite the recognized relevance of chirality in drug development, the major limitation remains the access to optically pure substances, since the enantioselective synthesis of chiral compounds is still very challenging. In this context, asymmetric catalysis emerges as an attractive approach in the enantioselective synthesis of chiral molecules, avoiding the use of stoichiometric chiral reagents and the often difficult and tedious enantiomer resolution protocols. Additionally, compliance with the green chemistry guidelines must also be addressed in the conception of new synthetic methods, prioritizing the use of mild reagents along with smooth, safe, and practical reaction conditions. To the best of our knowledge, there have been no reports of enantioselective approaches toward the synthesis of chiral imidazo[1,2-*a*]pyridines derivatives described in the literature so far. Therefore, in this work, we report a simple and robust catalytic asymmetric conjugate addition of 2-arylimidazo[1,2-*a*]pyridines to α,β-unsaturated 2-acylimidazoles in the presence of a rhodium-based chiral Lewis acid catalyst, using mild reaction conditions and with very high stereoselectivity. A huge variety of asymmetric catalysts have been devised for promoting enantioselective conjugate addition reactions,^13^ and our main focus was to employ a catalytic system with a strong background of elevated enantioselectivity associated with low catalyst loading. Recently, we have reported a new enantioselective synthesis of the active pharmaceutical ingredient (API) brivaracetam, using a “chiral-at-metal” catalyst developed by Meggers’ group.^14^ In several works reported,^15−20^ including ours, this catalyst demonstrated very good levels of stereoinduction, so we decided to evaluate if it would suit our purposes in this work.

## Results and Discussion

We started our study by evaluating the reaction between α,β-unsaturated 2-acylimidazole **1a** (0.1 mmol) and 2-phenylimidazo[1,2-*a*]pyridine **2a** (1 equiv), using 2.0 mol % catalyst Λ-RhS and dichloromethane as solvent (0.1 M) under air atmosphere. The reaction was monitored by TLC analysis and interrupted after total consumption of starting materials was observed. After 3 h at room temperature, product **(*****R*****)-3aa** was obtained in 99% yield with an enantiomeric ratio (er) of >99:1 (Table 1, entry 1). Inspired by this result and interested in the development of an ecofriendly synthetic protocol, we tested the same reaction condition in the presence of several green solvents, such as ethanol, ethyl acetate, 2-methyltetrahydrofuran and dimethyl carbonate (Table 1, entries 2–5). To our delight, in reaction times ranging between 3 and 4 h, product **(*****R*****)-3aa** was obtained with high yields (97% up to 99%) and maintaining er > 99:1. To continue our investigation, ethanol was selected as the best solvent for this protocol. Besides providing a high yield and er for **(*****R*****)-3aa** (99%, er > 99:1), it is known that ethanol can be obtained by fermentation of sugar-containing feedstock, being a sustainable choice in replacement of organic solvents derived from fossil resources.^21^ When the catalyst loading of Λ-RhS was reduced to 1.0 mol % and then to 0.5 mol %, both yield and er of product **(*****R*****)-3aa** remained practically the same (Table 1, entries 6 and 7). It is worth mentioning that with a catalyst loading of 0.5 mol % (Table 1, entry 7) the reaction required a bit more time to be completed. By increasing the substrate concentration from 0.1 to 0.25 M (Table 1, entry 8) and elevating the reaction scale from 0.1 to 0.25 mmol (Table 1, entry 9), compound **(*****R*****)-3aa** was successfully obtained with no significant alterations in yield or er (entries 8 and 9). No product was afforded in the absence of the rhodium catalyst (Table 1, entry 10), which demonstrates the catalyst is essential for the reaction to occur. We identified the conditions described in entry 9 of Table 1 as the best for this reaction. Under these conditions, the target compound was obtained with a very high yield and er, using only 0.5 mol % rhodium chiral catalyst. It is important to highlight that such levels of enantioselectivity associated with a much-reduced catalyst loading are not reported very often in the literature.

**Table 1:**
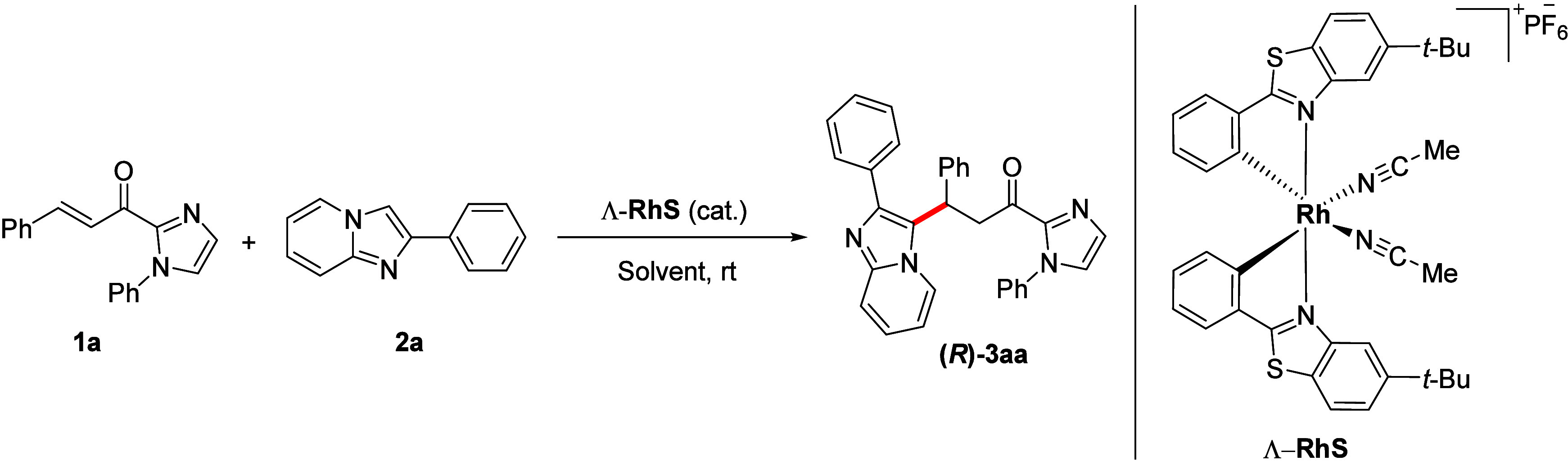
Optimization of Reaction Conditions between **1a** and **2a** Employing the Chiral Lewis Acid Catalyst Λ-RhS

entry^a^	Λ-RhS (mol %)	solvent	time (h)	yield (%)^b^	er^c^
1	2.0	DCM	3	99	>99:1
2	2.0	EtOH	3	99	>99:1
3	2.0	EtOAc	4	97	>99:1
4	2.0	2-MeTHF	4	98	>99:1
5	2.0	(MeO)_2_CO	3	99	>99:1
6	1.0	EtOH	3	99	>99:1
7	0.5	EtOH	6	97	>99:1
8^d^	0.5	EtOH	3	95	>99:1
**9**^d^^,^^e^	**0.5**	**EtOH**	**4**	**98**	**>99:1**
10^e^		EtOH	24		

a Conditions: **1a** (0.1 mmol), **2a** (1 equiv), concentration 0.1 M, room temperature, air atmosphere.

b Isolated yields.

c Enantioselectivities of **(*****R*****)-3aa** determined by HPLC on chiral stationary phase.

d Concentration of 0.25 M.

e Reaction scale of 0.25 mmol.

f Reaction in the absence of catalyst.

**Scheme 2:**
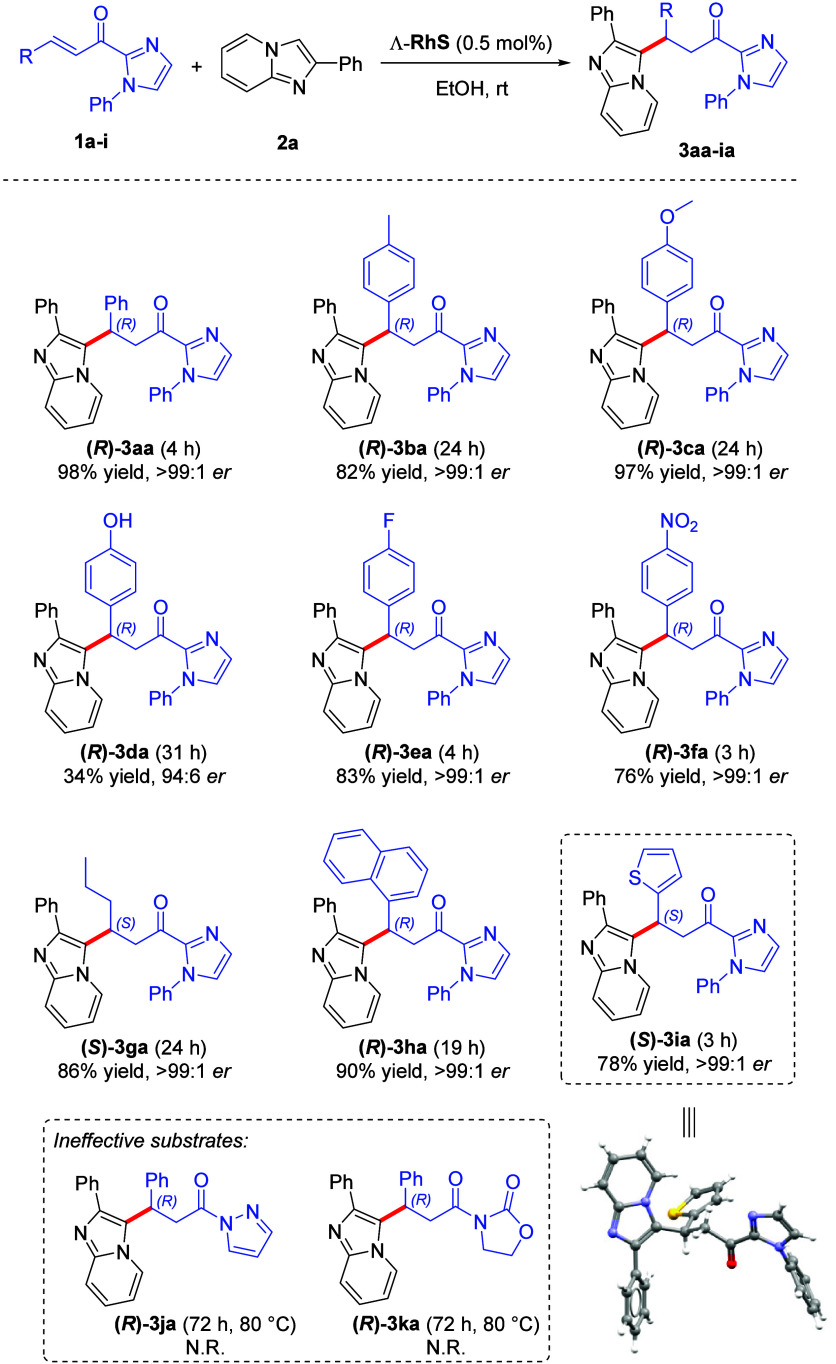
Substrate Scope Evaluation for α,β-Unsaturated 2-Acylimidazoles **1** Reaction conditions: **1** (0.25 mmol), **2** (0.25 mmol), Λ-RhS (0.5 mol %), EtOH (1 mL) at room temperature, under open air; isolated yields enantioselectivity determined by chiral HPLC analysis on a chiral stationary phase. (N.R. = No reaction).

**Scheme 3:**
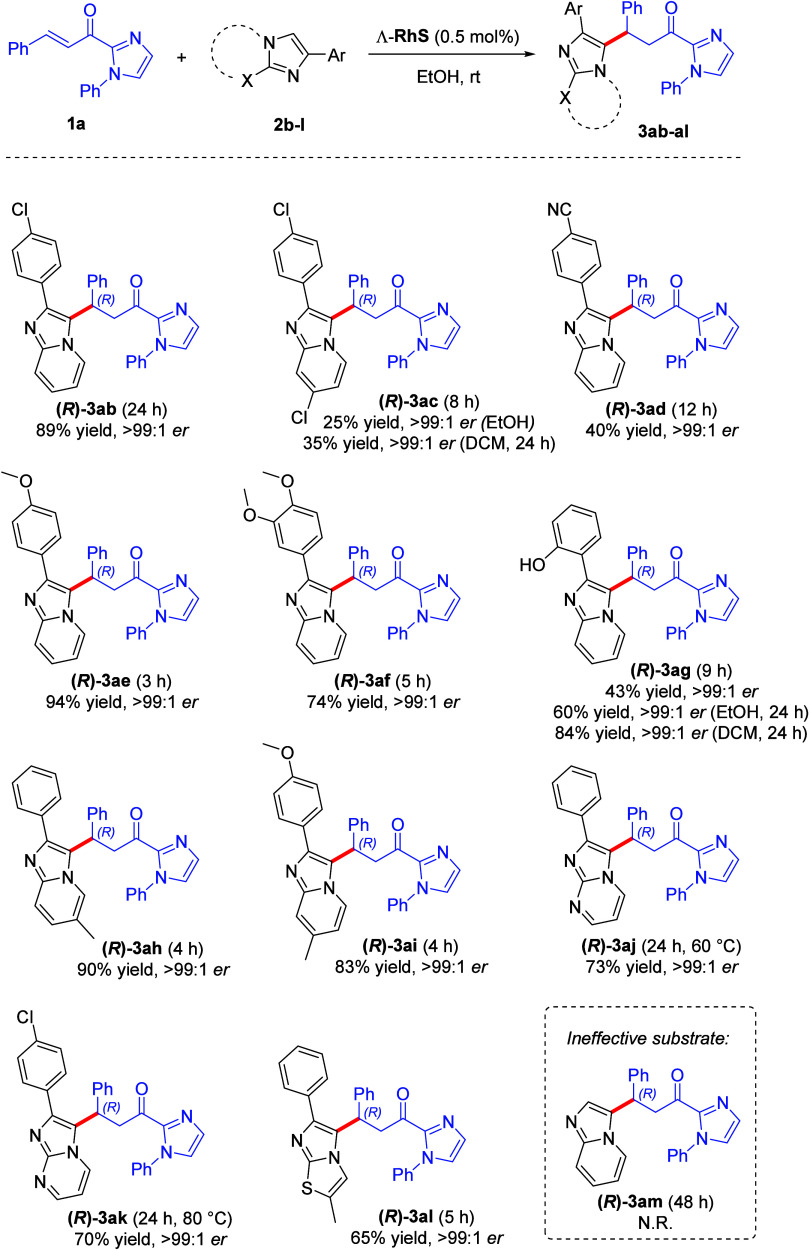
Substrate Scope Evaluation for Imidazo[1,2-*a*]pyridines **2** Reaction conditions: **1** (0.25 mmol), **2** (0.25 mmol), Λ-RhS (0.5 mol %), EtOH (1 mL) at room temperature, under open air; isolated yields enantioselectivity determined by chiral HPLC analysis on a chiral stationary phase.

The next step was the substrate scope evaluation, as summarized in Schemes 2 and 3. First, we evaluated the influence of the substituent at the β position of the α,β-unsaturated 2-acylimidazoles **1** (Scheme 2). Substrates bearing electron-donating or -withdrawing groups were employed and corresponding products **(*****R*****)-3ba**–**(*****R*****)-3fa** could be obtained in excellent yields and no changes were observed for the er. The reactions that afforded products **(*****R*****)-3ba** and **(*****R*****)-3ca** required more time to reach completion, which was already expected due to the presence of electron-donating groups that make the Michael acceptor less electrophilic. In exception, product **(*****R*****)-3da** showed a yield of only 34%, a result that we attributed to the low solubility observed for this substrate in the reaction medium. The substitution of the aromatic group at the β position of the electrophile was well tolerated, and compound **(*****S*****)-3ga**, with an alkyl chain, was obtained in 86% yield and again er > 99:1. The reactions employing substrates containing 1-naphthyl **(*****R*****)-3ha** and 2-thienyl substituent **(*****S*****)-3ia** proceeded successfully, providing the corresponding products in yields of 90 and 78% respectively, and keeping the same er. The absolute configuration of product **(*****S*****)-3ia** was determined by single-crystal X-ray analysis and assigned *S* (see Supporting Information for details). We also tested others Michael acceptors as α,β-unsaturated *N*-acyl pyrazol and 2-acyl oxazolidinone. Even under heating and longer reaction times, the respective conjugate addition products (or any products) were not observed for both reactions, **(*****R*****)-3ja** and **(*****R*****)-3ka**.

Furthermore, we evaluated the reaction performance regarding variation at the 2-phenylimidazo[1,2-*a*]pyridine substrate (Scheme 3). The presence of an electron-withdrawing group −Cl at the phenyl ring provided the corresponding product **(*****R*****)-3ab** in 89% yield and er > 99:1. However, when a second −Cl group was attached directly at the imidazopyridine core, product **(*****R*****)-3ac** showed a yield of 25%, but no alteration regarding er. This result can be explained by an expected decrease in imidazopyridine nucleophilicity caused by the presence of an electron-withdrawing group. The presence of a strong electron-withdrawing group such as −CN brought the yield down to 40% for product **(*****R*****)-3ad**. In turn, imidazopyridines containing electron-donating groups afforded corresponding products **(*****R*****)-3ae**–**(*****R*****)-3ai** in good yields ranging from 43% to 94% and er > 99:1. In exception, product **(*****R*****)-3ag** with a −OH group at the phenyl ring showed a yield of only 43%, a result that we attributed once again to the lower solubility observed for this substrate in the reaction medium. Even when we tested the same reaction in a time of 24 h, product **(*****R*****)-3ag** could be afforded with a yield of 60% (er > 99:1).

In an attempt to enhance the yield of products **(*****R*****)-3ac** and **(*****R*****)-3ag**, we performed these two reactions using DCM in substitution to ethanol, since both imidazo[1,2-*a*]pyridines **2c** and **2g** used as starting materials showed better solubility in this solvent (Scheme 3). For product **(*****R*****)-3ac**, after a 24 h period the yield was slightly increased to 35% and er remained the same (>99:1). However, for product **(*****R*****)-3ag**, a very significant improvement in yield was observed when the reaction was carried out in DCM for 24 h (from 43% to 84%), without affecting er.

The reactions employing other imidazole derivatives–such as imidazopyrimidines and imidazothiophene–also afforded the corresponding products with high yields and er. For the imidazopyrimidines the reaction required to be performed under heating and for longer reaction times, probably because of the lower reactivity expected for these compounds compared to imidazopyridines. Even so, the products **(*****R*****)-3aj** and **(*****R*****)-3ak** could be obtained in yields of 73 and 70% respectively, with the same standard enantioselectivity observed for the previous compounds. The reaction using imidazothiophene as the substrate provided the corresponding product **(*****R*****)-3al** in a yield of 65% with er > 99:1 at room temperature for 5 h. Despite the successful results found with 2-arylimidazo[1,2-*a*]pyridines, when we performed a reaction using commercially available imidazo[1,2-*a*]pyridine **2m**, corresponding product **(*****R*****)-3am** could not be afforded, even after 48 h of reaction.

**Scheme 4:**
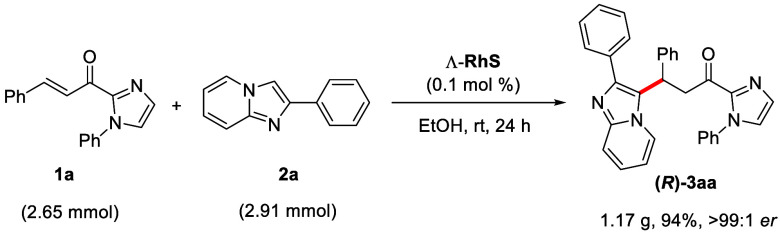
Gram-Scale Experiment

We also evaluated the scalability of the reaction by performing a gram-scale experiment. To our delight, the reaction proceeded as expected, affording product **(*****R*****)-3aa** in 94% yield (1.17 g) with er > 99:1. During the studies of scalability, we also found that the catalyst loading could be reduced even beyond the previously established 0.5 mol %, reaching an impressive 0.1 mol % (2.27 mg) loading of the catalyst Λ-RhS. (Scheme 4).

**Scheme 5:**
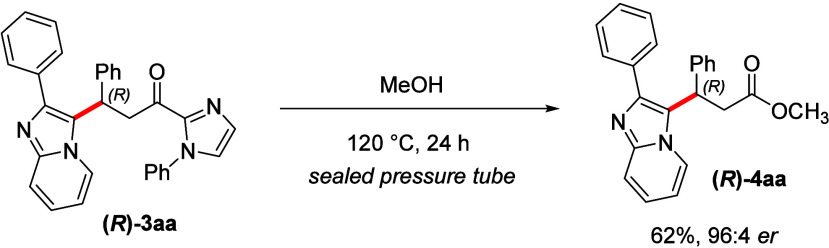
Synthetic Transformation for Product **(*****R*****)-3aa**

Moreover, we investigated the transformation of the imidazole scaffold to useful synthetic building blocks. By adapting the methodology described by Morimoto and Ohshima,^22^ we found that the acyl imidazole moiety in product **(*****R*****)-3aa** could be converted into optically active ester **(*****R*****)-4aa** with 62% yield and almost unaltered er of 96:4 (Scheme 5).

**Scheme 6:**
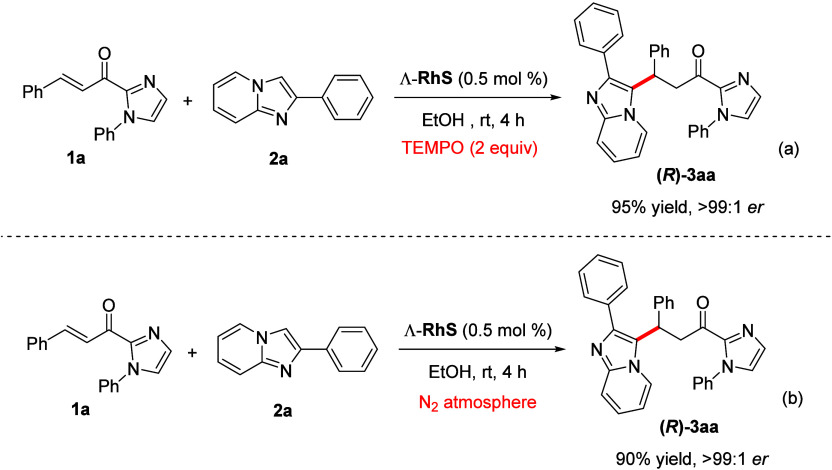
TEMPO Trapping and Inert Atmosphere Experiments

In terms of mechanism, this reaction seems to behave like a traditional Friedel–Crafts Alkylation. A radical pathway was ruled out when the use of TEMPO did not interfere with the formation or yield of the target product (eq (a), Scheme 6). We also demonstrated the reaction does not require the presence of oxygen, since degasification of the medium and use of inert atmosphere provided the same outcome as the standard conditions (eq (b), Scheme 6).

**Scheme 7:**
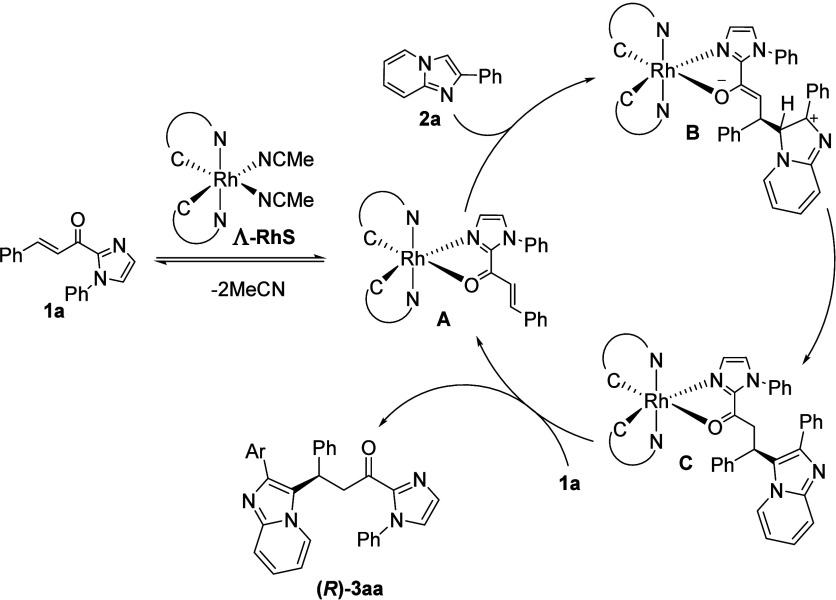
Proposed Mechanism for the Reaction between **1** and **2** Promoted by the Catalyst Λ-RhS

Based on the previous information, we proposed a hypothetical mechanism involved in this transformation (Scheme 7). First, substrate **1a** was activated by chiral rhodium(III) complex through bidentate N,O-coordination, to obtain intermediate **A**. In the sequence, compound **2a** attacks this intermediate to form **B**. The next step consists of rearomatization of the imidazo[1,2-*a*]pyridine core with delivery of the proton to the Rh enolate, leading to intermediate **C**, which releases product **(*R*)-3aa** by ligand exchange with **1a**, while a new catalytic cycle is restarted (Scheme 7). Regarding the absolute stereochemistry of the conjugate addition products, a stereoinduction model has already been established for this catalytic system by Meggers and co-workers.^23a^

**Figure 2:**
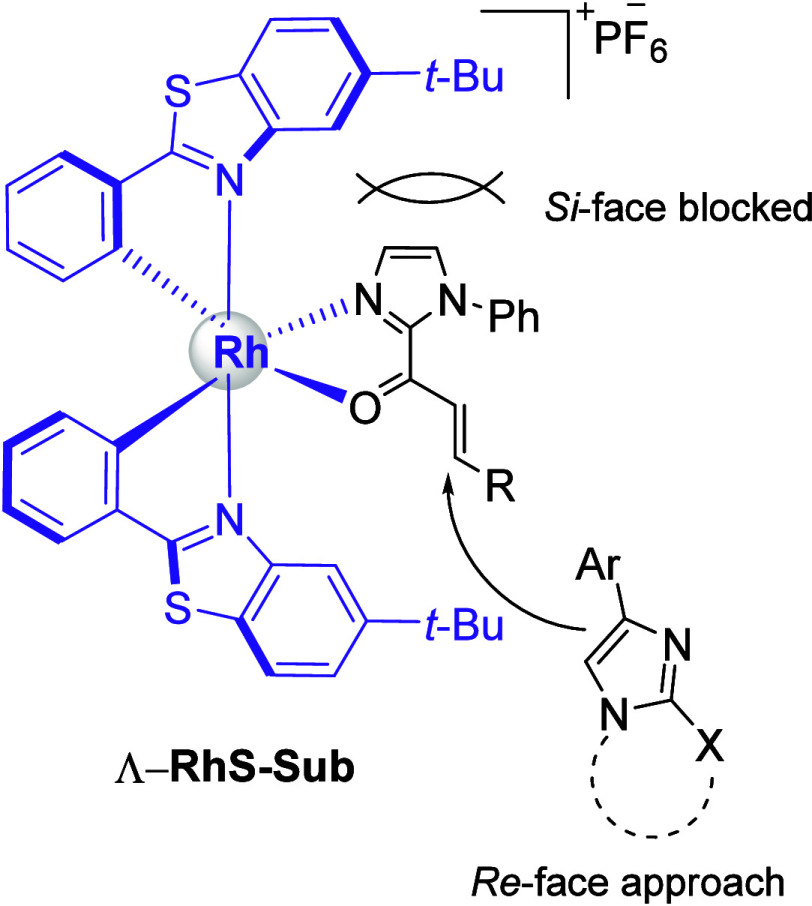
Trajectory of attack proposed to account for the stereochemical outcome of the conjugate addition products.

The authors demonstrated that when the α,β-unsaturated 2-acylimidazole coordinates to the Rh, one of the prochiral faces of the α,β-unsaturated double bond is shielded by the *tert*-butyl group of one of the benzothiazole ligands. In Figure 2 we can see substrate **1** coordinated to the Λ-RhS catalyst. The spatial arrangement of the ligands shields the *Si* face of the β carbon, directing the nucleophile approach from the *Re* face. The absolute stereochemistry of product depends on the relative priority between the substituents previously attached to the carbon under attack and the incoming nucleophile. This rationalization has led to the correct stereochemical outcome in several other works that employed this class of catalysts^23^ and in our case it was confirmed by the X-ray analysis of compound **(*****S*****)-3ia**, which was proven to present *S* configuration, compatible with the model previously presented (see Supporting Information).

In this work, we successfully achieved an enantioselective conjugate addition of 2-arylimidazo[1,2-*a*]pyridines to α,β-unsaturated 2-acylimidazoles. The protocol was also amenable to other imidazole-derived compounds such as imidazopyrimidines and imidazothiophene. The method employed a chiral Rh complex as an asymmetric catalyst and was proven highly efficient in terms of catalytic performance by providing an enantioselectivity of >99:1 for almost all products, using a very reduced catalyst loading (down to 0.5 or 0.1 mol % in gram-scale). The protocol also includes the use of sustainable solvents such as ethanol, mild reaction conditions, practical and safe protocol and high atomic economy. As far as we know, this is the first example of an enantioselective derivatization of 2-arylimidazo[1,2-*a*]pyridines, which now allows for the individual evaluation of its enantiomers regarding biological activities and open up new avenues for drug development based on this class of compounds.

## Experimental Section

### General

Unless otherwise noted, all reagents were obtained from commercial sources and were used without any further purification. Solvents of technical grade were purified via distillation prior to use (hexane, ethyl acetate, dichloromethane, and methanol), and solvents of PA quality were used without further purification. THF was distilled under nitrogen from sodium/benzophenone. HPLC-grade hexane, ethanol, and 2-propanol were used without further purification. Column chromatography was performed using Merck silica gel (230–400 mesh). Thin layer chromatography (TLC) was performed using Merck Silica Gel 60 F_254_, 0.20 mm thickness. For visualization, TLC plates were either placed under ultraviolet light, stained with iodine vapor and heating with acidic vanillin solution. The melting points were measured using Incoterm-5036 (2302/22) equipment without prior calibration. All proton nuclear magnetic resonance spectra (^1^H NMR) and proton-decoupled carbon-13 nuclear magnetic resonance spectra (^13^C{^1^H} NMR) were recorded at 400 and 100 MHz, respectively, on a Bruker AVANCE DRX-400 spectrometer. Spectra were recorded in CDCl_3_ and DMSO-*d*_6_ and chemical shifts (δ) were reported in parts per million (ppm). For ^1^H NMR spectra tetramethylsilane (TMS) was used as an internal reference (δ = 0.00 ppm), and all chemical shifts were reported relative to it. For ^13^C NMR spectra the signal of CDCl_3_ (δ = 77.2 ppm) or DMSO-*d*_6_ (δ = 39.5 ppm) were used as references to report chemical shifts. For ^1^H NMR spectra data are reported as follows: chemical shift (δ), multiplicity and coupling constant (*J*). Multiplicity of peaks are described as singlets (*s*), doublets (*d*), doublet of doublets (*dd*), doublet of doublets of doublets (*ddd*), triplets (*t*), doublet of triplets (*dt*), and multiplets (*m*). Chiral HPLC chromatography was performed using a Shimadzu UFLC Prominence 20-A HPLC employing a Daicel Chiralpak IG-3 chiral analytical column (150 × 4.6 mm i.d.) using the *n*-hexane/ethanol or *n*-hexane/2-propanol isocratic solvent system as mobile phase. The column temperature was 25 °C, and UV absorption was measured at 278 nm. Optical rotations were measured on a Schmidt–Haensch Polartronic E polarimeter equipped with a sodium lamp (589 nm) using a 0.95 dm long cell and reported as follows: [α]_D_^*T*^, (*c* = g·mL^–1^, solvent). High-resolution mass spectra were recorded on a Waters Xevo G2-XS instrument (QTOF analyzer) using the ESI technique.

### General Route for the Synthesis of 2-Acylimidazoles **1a**–**i**^24^

To a 100 mL vacuum flame-dried round-bottomed flask was added 1.44 g of *N*-phenylimidazole (10 mmol, 1.0 equiv) and 10 mL of anhydrous THF. Then, the mixture was cooled to −78 °C (using acetone and liquid N_2_) over 30 min followed by the addition of 5.04 mL of *n*-butyllithium in hexane (2.48 M, 12.5 mmol, 1.25 equiv). The acetone bath was removed and the reaction was warmed up to room temperature over 2 h. The reaction was cooled to −78 °C for 30 min and 1.80 mL of *t*-butyl chloroacetate (1.25 equiv) was added as a single portion. The acetone bath was removed and the reaction was warmed up to room temperature over 1 h. The reaction was diluted with 100 mL of EtOAc and washed with NaHCO_3_ (sat. solution, 2 × 50 mL) and brine (2 × 50 mL). The aqueous layer was separated and extracted 3 × 50 mL with EtOAc. The organic layers were combined and dried with Na_2_SO_4_. The drying agent was removed by filtration and the filtrate was concentrated under vacuum. Without any purification, the obtained product 2-chloro-1-(1-phenyl-1*H*-imidazol-2-yl)ethanone was used in the next step. To a 100 mL round-bottomed flask containing 2-chloro-1-(1-phenyl-1*H*-imidazol-2-yl)ethanone was added 16 mL of toluene and 4.72 g of triphenylphosphine (18 mmol, 1.8 equiv). The reaction was refluxed for 16 h. The reaction was cooled to room temperature and then diluted with 30 mL of 1 M HCl. The aqueous layer was separated and washed with diethyl ether (3 × 50 mL). To the aqueous layer was then added Na_2_CO_3_ (solid) until reaching pH 9. The precipitated solid, the Wittig reagent, was filtered under vacuum and washed with H_2_O and diethyl ether. To a solution of Wittig reagent (1.5 mmol, 1 equiv) in toluene (7.5 mL) was added the corresponding aldehyde (1.5 equiv). The reaction was refluxed overnight. The solvent was removed under vacuum, and the crude product was purified by flash chromatography on silica gel (EtOAc/hexane) to produce α,β-unsaturated alkenes **1a**–**i**. The NMR spectra of the compounds corroborate the data found in the literature,^25^ with exception of compounds **1d** and **1h** that have not been described so far.

#### (*E*)-3-(4-Hydroxyphenyl)-1-(1-phenyl-1*H*-imidazol-2-yl)prop-2-en-1-one (**1d**)

Yellow solid obtained by column chromatography on silica gel using hexane and ethyl acetate (50:50) as eluent with 26% yield (113.5 mg). Melting point: 88–90 °C. Chemical formula: C_18_H_14_N_2_O_2_. ^1^H NMR (400 MHz, Chloroform-*d*) δ 7.82–7.27 (*m*, 12H), 6.99 (*d*, *J* = 8.5 Hz, 1H), 6.88 (*d*, *J* = 8.2 Hz, 1H). ^13^C{^1^H} NMR (100 MHz, DMSO) δ 160.2, 143.5, 143.2, 142.9, 138.4, 130.7, 129.5, 128.8, 128.3, 128.0, 125.9, 125.5, 119.6, 116.0. HRMS *m*/*z* calculated for C_18_H_15_N_2_O_2_ [M + H]^+^ = 291.1113. Found: 291.1113.

#### (*E*)-3-(Naphthalen-1-yl)-1-(1-phenyl-1*H*-imidazol-2-yl)prop-2-en-1-one (**1h**)

Yellow solid obtained by column chromatography on silica gel using hexane and ethyl acetate (90:10) as eluent with 42% yield (203.9 mg). Melting point: 90–92 °C. Chemical formula: C_22_H_16_N_2_O. ^1^H NMR (400 MHz, Chloroform-*d*) δ 8.57 (*d*, *J* = 15.7 Hz, 1H), 8.18–8.08 (*m*, 2H), 7.96 (*d*, *J* = 7.2 Hz, 1H), 7.85–7.76 (*m*, 2H), 7.43 (*dtt*, *J* = 7.3, 5.3, 2.2 Hz, 6H), 7.31–7.28 (*m*, 3H), 7.17 (*d*, *J* = 0.9 Hz, 1H). ^13^C{^1^H} NMR (100 MHz, CDCl_3_) δ 179.2, 144.2, 140.5, 138.7, 133.8, 132.1, 132.0, 131.0, 130.1, 129.1, 128.9, 128.8, 127.5, 127.0, 126.2, 126.1, 125.6, 125.6, 124.9, 123.5. HRMS *m*/*z* calculated for C_22_H_17_N_2_O [M + H]^+^ = 325.1341. Found: 325.1338.

### General Route for the Synthesis of Imidazo[1,2-*a*]pyridines **2a**–**i**

Compounds **2a**–**l** were synthesized and purified according to the procedure reported in literature.^26^

### Synthesis of Λ-RhS Catalyst

The catalyst Λ-RhS was synthesized according to previous published procedure.^27^

### General Procedure for the Preparation of Compound **(*R*)-3aa**

2-Acylimidazoles (0.25 mmol), 2-arylimidazo[1,2-*a*]pyridines or derivatives (0.25 mmol), rhodium chiral catalyst Λ-RhS (0.5 mol %, 1.07 mg), and ethanol as the reaction solvent (1 mL) were placed in a test tube equipped with a magnetic stir bar at room temperature. Afterward, the reaction progress was monitored by TLC. When the transformation was complete (total consumption of starting materials), the reaction mixture was concentrated under vacuum in a rotatory evaporator. The crude product was purified by silica gel column chromatography with a mixture of ethyl acetate/hexane to afford the correspondent product.

#### (*R*)-3-Phenyl-1-(1-phenyl-1*H*-imidazol-2-yl)-3-(2-phenylimidazo[1,2-*a*]pyridin-3-yl)propan-1-one **(*R*)-3aa**

Slightly yellow solid obtained by column chromatography on silica gel using hexane and ethyl acetate (50:50–30:70) as eluent with 98% yield (114.7 mg). Melting point: 91–93 °C. Chemical formula: C_31_H_24_N_4_O. [α]_D_^20^ + 129.6 (*c* = 0.0020, CH_2_Cl_2_). ^1^H NMR (400 MHz, Chloroform-*d*) δ 7.83–7.78 (*m*, 1H), 7.68–7.60 (*m*, 3H), 7.38–7.15 (*m*, 13H), 7.03 (*ddd*, *J* = 8.8, 4.7, 1.4 Hz, 3H), 6.71–6.64 (*m*, 1H), 5.68 (*t*, *J* = 7.8 Hz, 1H), 4.23 (*dd*, *J* = 15.8, 7.4 Hz, 1H), 3.79 (*dd*, *J* = 15.8, 8.3 Hz, 1H). ^13^C{^1^H} NMR (100 MHz, CDCl_3_) δ 188.1, 144.9, 143.9, 142.2, 139.8, 138.0, 134.2, 129.7, 129.1, 129.0, 129.0, 128.9, 128.9, 128.8, 128.5, 128.0, 127.3, 127.1, 125.8, 124.8, 124.8, 120.9, 117.5, 112.4, 41.6, 35.9. Enantiomeric ratio established by HPLC analysis using a Chiralpak IG-3 column, er > 99:1 (HPLC: IG-3 column, λ 278 nm, *n*-hexane/ethanol = 70:30, flow rate 1.0 mL min^–1^, 25 °C, tr (peak 1) = 10.937 min and tr (peak 2) = 14.329 min. HRMS *m*/*z* calculated for C_31_H_25_N_4_O [M + H]^+^ = 469.2029. Found: 469.2035.

#### (*R*)-1-(1-Phenyl-1*H*-imidazol-2-yl)-3-(2-phenylimidazo[1,2-*a*]pyridin-3-yl)-3-(p-tolyl)propan-1-one **(*R*)-3ba**

Slightly yellow solid obtained by column chromatography on silica gel using hexane and ethyl acetate (50:50–30:70) as eluent with 82% yield (99.3 mg). Melting point: 90–92 °C. Chemical formula: C_32_H_26_N_4_O. [α]_D_^20^ + 269.9 (*c* = 0.0001, CH_2_Cl_2_). ^1^H NMR (400 MHz, Chloroform-*d*) δ 7.81 (*d*, *J* = 6.9 Hz, 1H), 7.70–7.60 (*m*, 3H), 7.39–6.98 (*m*, 16H), 6.71–6.65 (*m*, 1H), 4.20 (*dd*, *J* = 15.7, 7.4 Hz, 1H), 3.77 (*dd*, *J* = 15.7, 8.4 Hz, 1H), 2.31 (*s*, 3H). ^13^C{^1^H} NMR (100 MHz, CDCl_3_) δ 188.2, 144.8, 142.2, 138.1, 136.7, 136.6, 134.1, 129.8, 129.6, 129.0, 129.0, 128.8, 128.5, 128.0, 127.3, 127.2, 127.0, 125.8, 125.0, 124.8, 124.8, 121.0, 117.4, 112.3, 41.6, 35.7, 21.1. Enantiomeric ratio established by HPLC analysis using a Chiralpak IG-3 column, er > 99:1 (HPLC: IG-3 column, λ 278 nm, *n*-hexane/ethanol = 70:30, flow rate 1.0 mL min^–1^, 25 °C, tr (peak 1) = 11.664 min and tr (peak 2) = 14.277 min. HRMS *m*/*z* calculated for C_32_H_27_N_4_O [M + H]^+^ = 483.2185. Found: 483.2185.

#### (*R*)-3-(4-Methoxyphenyl)-1-(1-phenyl-1*H*-imidazol-2-yl)-3-(2-phenylimidazo[1,2-*a*]pyridin-3-yl)propan-1-one **(*R*)-3ca**

Slightly yellow oil obtained by column chromatography on silica gel using hexane and ethyl acetate (30:70) as eluent with 97% yield (121.1 mg). Chemical formula: C_32_H_26_N_4_O_2_. [α]_D_^20^ + 76.5 (*c* = 0.0011, CH_2_Cl_2_). ^1^H NMR (400 MHz, Chloroform-*d*) δ 7.81 (*d*, *J* = 6.9 Hz, 1H), 7.62 (*dq*, *J* = 8.4, 4.6, 3.4 Hz, 3H), 7.39–7.26 (*m*, 7H), 7.17–7.12 (*m*, 3H), 7.04–6.98 (*m*, 3H), 6.81 (*d*, *J* = 8.7 Hz, 2H), 6.65 (*t*, *J* = 6.8 Hz, 1H), 5.64 (*t*, *J* = 7.8 Hz, 1H), 4.22 (*dd*, *J* = 15.7, 7.6 Hz, 1H), 3.79–3.70 (*m*, 4H). ^13^C{^1^H} NMR (100 MHz, CDCl_3_) δ 188.2, 158.5, 144.9, 143.9, 142.1, 138.0, 134.4, 131.6, 129.6, 128.9, 128.9, 128.7, 128.4, 128.1, 127.8, 127.2, 125.7, 124.8, 124.5, 121.0, 117.5, 114.3, 112.1, 55.3, 41.7, 35.2. Enantiomeric ratio established by HPLC analysis using a Chiralpak IG-3 column, er > 99:1 (HPLC: IG-3 column, λ 278 nm, *n*-hexane/ethanol = 70:30, flow rate 1.0 mL min^–1^, 25 °C, tr (peak 1) = 14.290 min and tr (peak 2) = 35.354 min. HRMS *m*/*z* calculated for C_32_H_27_N_4_O_2_ [M + H]^+^ = 499.2134. Found: 499.2134.

#### (*R*)-3-(4-Hydroxyphenyl)-1-(1-phenyl-1*H*-imidazol-2-yl)-3-(2-phenylimidazo[1,2-*a*]pyridin-3-yl)propan-1-one **(*R*)-3da**

Yellow solid obtained by column chromatography on silica gel using hexane and ethyl acetate (30:70) as eluent with 34% yield (41.1 mg). Melting point: 163–165 °C. Chemical formula: C_31_H_24_N_4_O_2_. [α]_D_^20^ – 5.34 (*c* = 0.0019, AcOEt). ^1^H NMR (400 MHz, DMSO-*d*_*6*_) δ 9.40 (*s*, 1H), 8.04 (*d*, *J* = 6.9 Hz, 1H), 7.62–7.53 (*m*, 4H), 7.42–7.30 (*m*, 6H), 7.27–7.21 (*m*, 1H), 7.15 (*s*, 1H), 7.12–7.06 (*m*, 2H), 6.99 (*d*, *J* = 8.5 Hz, 2H), 6.83 (*t*, *J* = 6.7 Hz, 1H), 6.70 (*d*, *J* = 8.5 Hz, 2H), 5.45 (*t*, *J* = 7.7 Hz, 1H), 4.30 (*dd*, *J* = 15.8, 7.6 Hz, 1H), 3.66 (*dd*, *J* = 15.8, 7.9 Hz, 1H). ^13^C{^1^H} NMR (100 MHz, DMSO) δ 188.2, 156.0, 144.0, 142.8, 141.6, 137.8, 134.7, 129.6, 129.5, 129.4, 128.7, 128.4, 128.3, 128.2, 128.1, 127.8, 127.7, 127.5, 125.5, 125.0, 124.3, 121.2, 117.0, 115.6, 112.0, 39.5, 38.9, 34.5. Enantiomeric ratio established by HPLC analysis using a Chiralpak IG-3 column, er = 94:6 (HPLC: IG-3 column, λ 278 nm, *n*-hexane/2-propanol = 80:20, flow rate 1.0 mL min^–1^, 25 °C, tr (peak 1) = 13.867 min and tr (peak 2) = 16.852 min. HRMS *m*/*z* calculated for C_31_H_25_N_4_O_2_ [M + H]^+^ = 485.1978. Found: 485.1978.

#### (*R*)-3-(4-Fluorophenyl)-1-(1-phenyl-1*H*-imidazol-2-yl)-3-(2-phenylimidazo[1,2-*a*]pyridin-3-yl)propan-1-one **(*R*)-3ea**

Yellow solid obtained by column chromatography on silica gel using hexane and ethyl acetate (50:50–30:70) as eluent with 83% yield (101.1 mg). Melting point: 108–110 °C. Chemical formula: C_31_H_23_FN_4_O. [α]_D_^20^ + 105.2 (*c* = 0.0020, CH_2_Cl_2_). ^1^H NMR (400 MHz, Chloroform-*d*) δ 7.70 (*d*, *J* = 6.9 Hz, 1H), 7.58–7.49 (*m*, 3H), 7.31–7.18 (*m*, 6H), 7.14–7.05 (*m*, 3H), 6.99–6.85 (*m*, 6H), 6.64–6.56 (*m*, 1H), 5.56 (*t*, *J* = 7.7 Hz, 1H), 4.15 (*dd*, *J* = 15.9, 7.7 Hz, 1H), 3.68 (*dd*, *J* = 15.9, 7.9 Hz, 1H). ^13^C{^1^H} NMR (100 MHz, CDCl_3_) δ 187.9, 163.0, 160.5, 145.1, 144.3, 142.1, 138.0, 135.5, 134.4, 129.7, 129.0, 129.0, 128.8, 128.7, 128.6, 128.4, 127.9, 127.3, 125.7, 124.6, 124.5, 120.6, 117.7, 116.0, 115.7, 112.2, 100.1, 41.7, 35.3. C–F coupling: (D) 161.77 (*d*, *J*_C–F_ = 245.9 Hz), (C) 128.66 (*d*, *J*_C–F_ = 8.0 Hz), (B) 124.54 (*d*, *J*_C–F_ = 2.3 Hz), (A) 115.85 (*d*, *J*_C–F_ = 21.3 Hz). Enantiomeric ratio established by HPLC analysis using a Chiralpak IG-3 column, er > 99:1 (HPLC: IG-3 column, λ 278 nm, *n*-hexane/ethanol = 70:30, flow rate 1.0 mL min^–1^, 25 °C, tr (peak 1) = 10.707 min and tr (peak 2) = 21.428 min. HRMS *m*/*z* calculated for C_31_H_24_FN_4_O [M + H]^+^ = 487.1934. Found: 487.1935.

#### (*R*)-3-(4-Nitrophenyl)-1-(1-phenyl-1*H*-imidazol-2-yl)-3-(2-phenylimidazo[1,2-*a*]pyridin-3-yl)propan-1-one **(*R*)-3fa**

Slightly brown oil obtained by column chromatography on silica gel using hexane and ethyl acetate (50:50–30:70) as eluent with 76% yield (97.3 mg). Chemical formula: C_31_H_23_N_5_O_3_. [α]_D_^20^ + 110.4 (*c* = 0.0026, CH_2_Cl_2_). ^1^H NMR (400 MHz, Chloroform-*d*) δ 8.01 (*d*, *J* = 8.5 Hz, 2H), 7.67 (*d*, *J* = 6.6 Hz, 1H), 7.55 (*d*, *J* = 9.0 Hz, 1H), 7.47 (*d*, *J* = 7.3 Hz, 2H), 7.31–7.18 (*m*, 8H), 7.11–7.05 (*m*, 1H), 7.02–6.93 (*m*, 4H), 6.61 (*t*, *J* = 6.8 Hz, 1H), 5.64 (*t*, *J* = 7.5 Hz, 1H), 4.24 (*dd*, *J* = 16.2, 7.9 Hz, 1H), 3.71 (*dd*, *J* = 16.3, 7.2 Hz, 1H). ^13^C{^1^H} NMR (100 MHz, CDCl_3_) δ 187.2, 147.6, 146.8, 145.1, 144.7, 141.8, 137.8, 134.1, 129.8, 129.0, 128.9, 128.5, 128.1, 128.0, 127.6, 125.6, 124.7, 124.0, 124.0, 119.6, 117.8, 112.5, 41.3, 35.8. Enantiomeric ratio established by HPLC analysis using a Chiralpak IG-3 column, er > 99:1 (HPLC: IG-3 column, λ 278 nm, *n*-hexane/2-propanol = 60:40, flow rate 1.0 mL min^–1^, 25 °C, tr (peak 1) = 18.659 min and tr (peak 2) = 20.339 min. HRMS *m*/*z* calculated for C_31_H_24_N_5_O_3_ [M + H]^+^ = 514.1879. Found: 514.1882.

#### (*S*)-1-(1-Phenyl-1*H*-imidazol-2-yl)-3-(2-phenylimidazo[1,2-*a*]pyridin-3-yl)hexan-1-one **(*S*)-3ga**

Yellow oil obtained by column chromatography on silica gel using hexane and ethyl acetate (50:50–40:60) as eluent with 86% yield (96.8 mg). Chemical formula: C_28_H_26_N_4_O. [α]_D_^20^ + 58.8 (*c* = 0.0007, CH_2_Cl_2_). ^1^H NMR (400 MHz, Chloroform-*d*) δ 8.20 (*d*, *J* = 6.9 Hz, 1H), 7.56–7.48 (*m*, 3H), 7.34–7.21 (*m*, 6H), 7.10–7.02 (*m*, 2H), 7.01–6.98 (*m*, 1H), 6.94–6.89 (*m*, 2H), 6.70 (*t*, *J* = 6.8 Hz, 1H), 4.15–4.02 (*m*, 1H), 3.62 (*dd*, *J* = 15.2, 7.7 Hz, 1H), 3.48 (*dd*, *J* = 15.3, 7.6 Hz, 1H), 1.80 (*ddt*, *J* = 19.2, 9.9, 5.3 Hz, 1H), 1.70–1.58 (*m*, 1H), 1.19–0.92 (*m*, 2H), 0.62 (*t*, *J* = 7.3 Hz, 3H). ^13^C{^1^H} NMR (100 MHz, CDCl_3_) δ 189.3, 144.7, 144.0, 142.6, 138.0, 135.1, 129.6, 129.3, 128.9, 128.7, 128.2, 127.6, 127.2, 125.6, 124.7, 123.7, 121.8, 117.7, 111.9, 42.8, 34.7, 31.8, 21.0, 13.8. Enantiomeric ratio established by HPLC analysis using a Chiralpak IG-3 column, er > 99:1 (HPLC: IG-3 column, λ 278 nm, *n*-hexane/ethanol = 80:20, flow rate 1.0 mL min^–1^, 25 °C, tr (peak 1) = 9.816 min and tr (peak 2) = 11.129 min. HRMS *m*/*z* calculated for C_28_H_27_N_4_O [M + H]^+^ = 435.2185. Found: 435.2185.

#### (*R*)-3-(Naphthalen-1-yl)-1-(1-phenyl-1*H*-imidazol-2-yl)-3-(2-phenylimidazo[1,2-*a*]pyridin-3-yl)propan-1-one **(*R*)-3ha**

Yellow solid obtained by column chromatography on silica gel using hexane and ethyl acetate (70:30–40:60) as eluent with 90% yield (117.3 mg). Melting point: 108–111 °C. Chemical formula: C_35_H_26_N_4_O. [α]_D_^20^ + 137.1 (*c* = 0.0003, CH_2_Cl_2_). ^1^H NMR (400 MHz, Chloroform-*d*) δ 7.92 (*d*, *J* = 8.4 Hz, 1H), 7.82 (*d*, *J* = 7.0 Hz, 1H), 7.79–7.75 (*m*, 1H), 7.66 (*d*, *J* = 8.2 Hz, 1H), 7.55–7.48 (*m*, 3H), 7.42–7.18 (*m*, 10H), 7.08–6.95 (*m*, 5H), 6.59–6.51 (*m*, 1H), 6.03–5.93 (*m*, 1H), 4.07 (*dd*, *J* = 17.4, 9.1 Hz, 1H), 3.99 (*dd*, *J* = 17.5, 6.6 Hz, 1H). ^13^C{^1^H} NMR (100 MHz, CDCl_3_) δ 188.0, 144.8, 144.4, 142.1, 138.1, 135.7, 135.1, 134.3, 131.2, 129.6, 129.6, 129.2, 129.0, 128.8, 128.2, 128.1, 127.9, 127.0, 126.6, 125.8, 125.8, 125.3, 124.7, 124.4, 124.1, 123.2, 120.5, 117.6, 112.2, 42.1, 33.9. Enantiomeric ratio established by HPLC analysis using a Chiralpak IG-3 column, er > 99:1 (HPLC: IG-3 column, λ 278 nm, *n*-hexane/ethanol = 70:30, flow rate 1.0 mL min^–1^, 25 °C, tr (peak 1) = 6.188 min and tr (peak 2) = 7.252 min. HRMS *m*/*z* calculated for C_35_H_27_N_4_O [M + H]^+^ = 519.2185. Found: 519.2186.

#### (*S*)-1-(1-Phenyl-1*H*-imidazol-2-yl)-3-(2-phenylimidazo[1,2-*a*]pyridin-3-yl)-3-(thiophen-2-yl)propan-1-one **(*S*)-3ia**

White solid obtained by column chromatography on silica gel using hexane and ethyl acetate (50:50) as eluent with 78% yield (92.9 mg). Melting point: 172–174 °C. Chemical formula: C_29_H_22_N_4_OS. [α]_D_^20^ + 127.2 (*c* = 0.0005, CH_2_Cl_2_). ^1^H NMR (400 MHz, Chloroform-*d*) δ 7.82 (*d*, *J* = 6.9 Hz, 1H), 7.53 (*t*, *J* = 8.7 Hz, 3H), 7.28–7.15 (*m*, 7H), 7.08–7.00 (*m*, 2H), 6.93–6.86 (*m*, 4H), 6.81 (*dd*, *J* = 5.9, 2.6 Hz, 1H), 6.77–6.74 (*m*, 1H), 6.56 (*t*, *J* = 6.8 Hz, 1H), 5.76 (*t*, *J* = 7.6 Hz, 1H), 4.20 (*dd*, *J* = 15.5, 7.7 Hz, 1H), 3.66 (*dd*, *J* = 15.5, 7.7 Hz, 1H). ^13^C{^1^H} NMR (100 MHz, CDCl_3_) δ 187.2, 145.2, 144.4, 144.0, 141.9, 137.8, 134.2, 129.6, 128.8, 128.8, 128.6, 128.4, 127.8, 127.2, 127.0, 125.5, 124.9, 124.7, 124.4, 124.4, 120.2, 117.5, 112.1, 42.3, 32.9. Enantiomeric ratio established by HPLC analysis using a Chiralpak IG-3 column, er > 99:1 (HPLC: IG-3 column, λ 278 nm, *n*-hexane/ethanol = 80:20, flow rate 1.0 mL min^–1^, 25 °C, tr (peak 1) = 20.171 min and tr (peak 2) = 23.282 min. HRMS *m*/*z* calculated for C_29_H_23_N_4_OS [M + H]^+^ = 475.1593. Found: 475.1592.

#### (*R*)-3-(2-(4-Chlorophenyl)imidazo[1,2-*a*]pyridin-3-yl)-3-phenyl-1-(1-phenyl-1*H*-imidazol-2-yl)propan-1-one **(*R*)-3ab**

Slightly yellow solid obtained by column chromatography on silica gel using hexane and ethyl acetate (50:50) as eluent with 89% yield (112.1 mg). Melting point: 82–86 °C. Chemical formula: C_31_H_23_ClN_4_O. [α]_D_^20^ + 139.6 (*c* = 0.0009, CH_2_Cl_2_). ^1^H NMR (400 MHz, Chloroform-*d*) δ 7.67 (*d*, *J* = 6.9 Hz, 1H), 7.54–7.40 (*m*, 3H), 7.14 (*dq*, *J* = 24.6, 7.2 Hz, 10H), 6.91 (*td*, *J* = 22.7, 21.9, 7.5 Hz, 5H), 6.47 (*t*, *J* = 6.8 Hz, 1H), 5.53 (*t*, *J* = 7.6 Hz, 1H), 4.07 (*dd*, *J* = 15.6, 7.2 Hz, 1H), 3.69 (*dd*, *J* = 15.7, 8.3 Hz, 1H). ^13^C{^1^H} NMR (100 MHz, CDCl_3_) δ 187.8, 144.9, 142.9, 141.8, 139.4, 137.7, 133.5, 133.0, 130.0, 129.4, 128.9, 128.7, 128.6, 128.4, 127.1, 126.9, 126.7, 125.4, 124.5, 124.4, 120.7, 117.3, 112.0, 41.3, 35.7. Enantiomeric ratio established by HPLC analysis using a Chiralpak IG-3 column, er > 99:1 (HPLC: IG-3 column, λ 278 nm, *n*-hexane/ethanol = 70:30, flow rate 1.0 mL min^–1^, 25 °C, tr (peak 1) = 9.587 min and tr (peak 2) = 14.046 min. HRMS *m*/*z* calculated for C_31_H_24_ClN_4_O [M + H]^+^ = 503.1638. Found: 503.1639.

#### (*R*)-3-(7-Chloro-2-(4-chlorophenyl)imidazo[1,2-*a*]pyridin-3-yl)-3-phenyl-1-(1-phenyl-1*H*-imidazol-2-yl)propan-1-one **(*R*)-3ac**

Yellow solid obtained by column chromatography on silica gel using hexane and ethyl acetate (50:50) as eluent with 25% yield (34.4 mg). Melting point: 80–84 °C. Chemical formula: C_31_H_22_Cl_2_N_4_O. [α]_D_^20^ + 100.2 (*c* = 0.0002, CH_2_Cl_2_). ^1^H NMR (400 MHz, Chloroform-*d*) δ 8.02–7.98 (*m*, 1H), 7.76 (*s*, 1H), 7.56 (*d*, *J* = 9.5 Hz, 1H), 7.43 (*d*, *J* = 8.4 Hz, 2H), 7.30 (*d*, *J* = 7.7 Hz, 2H), 7.25–7.17 (*m*, 5H), 7.13 (*d*, *J* = 7.8 Hz, 2H), 7.08–6.93 (*m*, 5H), 5.48 (*t*, *J* = 7.8 Hz, 1H), 4.03 (*dd*, *J* = 16.1, 7.1 Hz, 1H), 3.78 (*dd*, *J* = 16.0, 8.6 Hz, 1H). ^13^C{^1^H} NMR (100 MHz, CDCl_3_) δ 187.8, 143.9, 143.4, 142.0, 139.0, 137.9, 134.2, 132.9, 132.5, 130.4, 130.0, 129.7, 129.3, 129.1, 129.0, 128.7, 128.4, 127.4, 127.0, 126.3, 125.7, 122.5, 121.7, 120.6, 117.9, 41.5, 35.9. Enantiomeric ratio established by HPLC analysis using a Chiralpak IG-3 column, er > 99:1 (HPLC: IG-3 column, λ 278 nm, *n*-hexane/ethanol = 70:30, flow rate 1.0 mL min^–1^, 25 °C, tr (peak 1) = 14.828 min and tr (peak 2) = 26.935 min. HRMS *m*/*z* calculated for C_31_H_23_Cl_2_N_4_O [M + H]^+^ = 537.1249. Found: 537.1251.

#### (*R*)-4-(3-(3-oxo-1-phenyl-3-(1-phenyl-1*H*-imidazol-2-yl)propyl)imidazo[1,2-*a*]pyridin-2-yl)benzonitrile **(*R*)-3ad**

Yellow solid obtained by column chromatography on silica gel using hexane and ethyl acetate (50:50–30:70) as eluent with 40% yield (49.7 mg). Melting point: 72–74 °C. Chemical formula: C_32_H_23_N_5_O. [α]_D_^20^ + 128.4 (*c* = 0.0009, CH_2_Cl_2_). ^1^H NMR (400 MHz, Chloroform-*d*) δ 7.74 (*d*, *J* = 7.0 Hz, 1H), 7.67 (*d*, *J* = 8.4 Hz, 2H), 7.56–7.51 (*m*, 2H), 7.34–7.07 (*m*, 10H), 6.98 (*d*, *J* = 2.8 Hz, 2H), 6.94–6.89 (*m*, 2H), 6.60 (*t*, *J* = 6.8 Hz, 1H), 5.59 (*t*, *J* = 7.7 Hz, 1H), 4.13 (*dd*, *J* = 15.8, 7.3 Hz, 1H), 3.74 (*dd*, *J* = 15.8, 8.3 Hz, 1H). ^13^C{^1^H} NMR (100 MHz, CDCl_3_) δ 187.8, 145.3, 142.2, 142.0, 139.4, 139.2, 137.8, 132.1, 129.7, 129.5, 129.2, 129.0, 128.9, 127.4, 127.3, 126.9, 125.6, 125.1, 124.9, 121.9, 119.0, 117.8, 112.6, 111.2, 41.4, 35.8. Enantiomeric ratio established by HPLC analysis using a Chiralpak IG-3 column, er > 99:1 (HPLC: IG-3 column, λ 278 nm, *n*-hexane/ethanol = 70:30, flow rate 1.0 mL min^–1^, 25 °C, tr (peak 1) = 17.387 min and tr (peak 2) = 29.569 min. HRMS *m*/*z* calculated for C_32_H_24_N_5_O [M + H]^+^ = 494.1981. Found: 494.1981.

#### (*R*)-3-(2-(4-Methoxyphenyl)imidazo[1,2-*a*]pyridin-3-yl)-3-phenyl-1-(1-phenyl-1*H*-imidazol-2-yl)propan-1-one **(*R*)-3ae**

Yellow solid obtained by column chromatography on silica gel using hexane and ethyl acetate (40:60–20:80) as eluent with 94% yield (116.9 mg). Melting point: 93–95 °C. Chemical formula: C_32_H_26_N_4_O_2_. [α]_D_^20^ + 103.7 (*c* = 0.0028, CH_2_Cl_2_). ^1^H NMR (400 MHz, Chloroform-*d*) δ 7.68 (*d*, *J* = 6.8 Hz, 1H), 7.47 (*dd*, *J* = 16.2, 8.8 Hz, 3H), 7.17 (*ddd*, *J* = 27.8, 15.5, 6.8 Hz, 8H), 7.03–6.87 (*m*, 5H), 6.77 (*d*, *J* = 8.6 Hz, 2H), 6.50 (*t*, *J* = 6.5 Hz, 1H), 5.55 (*t*, *J* = 7.7 Hz, 1H), 4.11 (*dd*, J = 15.8, 7.4 Hz, 1H), 3.77–3.62 (*m*, 4H). ^13^C{^1^H} NMR (100 MHz, CDCl_3_) δ 188.1, 159.3, 144.8, 144.0, 142.0, 139.8, 137.9, 130.1, 129.4, 128.9, 128.8, 128.6, 127.1, 127.0, 126.9, 126.9, 125.6, 124.5, 124.2, 120.1, 117.2, 113.8, 111.8, 55.2, 41.5, 35.8. Enantiomeric ratio established by HPLC analysis using a Chiralpak IG-3 column, er > 99:1 (HPLC: IG-3 column, λ 278 nm, *n*-hexane/ethanol = 70:30, flow rate 1.0 mL min^–1^, 25 °C, tr (peak 1) = 18.817 min and tr (peak 2) = 43.283 min. HRMS *m*/*z* calculated for C_32_H_27_N_4_O_2_ [M + H]^+^ = 499.2134. Found: 499.2136.

#### (*R*)-3-(2-(3,4-Dimethoxyphenyl)imidazo[1,2-*a*]pyridin-3-yl)-3-phenyl-1-(1-phenyl-1*H*-imidazol-2-yl)propan-1-one **(*R*)-3af**

Yellow oil obtained by column chromatography on silica gel using hexane and ethyl acetate (60:40–10:90) as eluent with 74% yield (97.6 mg). Melting point:. Chemical formula: C_33_H_28_N_4_O_3_. [α]_D_^20^ + 99.6 (*c* = 0.0010, CH_2_Cl_2_). ^1^H NMR (400 MHz, Chloroform-*d*) δ 7.75 (*d*, *J* = 6.9 Hz, 1H), 7.57–7.51 (*m*, 1H), 7.31–7.25 (*m*, 3H), 7.23–7.17 (*m*, 3H), 7.16–7.04 (*m*, 6H), 7.00–6.93 (*m*, 3H), 6.80–6.76 (*m*, 1H), 6.58 (*td*, *J* = 6.8, 1.2 Hz, 1H), 5.62 (*t*, *J* = 7.7 Hz, 1H), 4.15 (*dd*, J = 15.9, 7.3 Hz, 1H), 3.87–3.77 (*m*, 4H), 3.69 (*s*, 3H). ^13^C{^1^H} NMR (100 MHz, CDCl_3_) δ 188.4, 148.8, 144.9, 144.1, 142.3, 140.0, 138.0, 129.6, 129.0, 129.0, 128.8, 127.4, 127.3, 127.0, 127.0, 125.7, 124.7, 124.3, 121.5, 120.5, 117.4, 112.2, 112.0, 111.1, 55.9, 55.9, 41.3, 35.7. Enantiomeric ratio established by HPLC analysis using a Chiralpak IG-3 column, er > 99:1 (HPLC: IG-3 column, λ 278 nm, *n*-hexane/ethanol = 70:30, flow rate 1.0 mL min^–1^, 25 °C, tr (peak 1) = 26.166 min and tr (peak 2) = 55.967 min. HRMS *m*/*z* calculated for C_33_H_29_N_4_O_3_ [M + H]^+^ = 529.2239. Found: 529.2241.

#### (*R*)-3-(2-(2-Hydroxyphenyl)imidazo[1,2-*a*]pyridin-3-yl)-3-phenyl-1-(1-phenyl-1*H*-imidazol-2-yl)propan-1-one **(*R*)-3ag**

Yellow solid obtained by column chromatography on silica gel using hexane and ethyl acetate (70:30–60:40) as eluent with 43% yield (51.6 mg). Melting point: 86–92 °C. Chemical formula: C_31_H_24_N_4_O_2_. [α]_D_^20^ + 27.9 (*c* = 0.0003, CH_2_Cl_2_). ^1^H NMR (400 MHz, Chloroform-*d*) δ 7.75 (*d*, *J* = 7.0 Hz, 1H), 7.52–7.48 (*m*, 2H), 7.30–7.10 (*m*, 11H), 6.99 (*dd*, *J* = 6.4, 0.9 Hz, 2H), 6.97–6.92 (*m*, 2H), 6.74 (*td*, *J* = 7.6, 1.2 Hz, 1H), 6.62 (*td*, *J* = 6.9, 1.2 Hz, 1H), 5.90 (*t*, *J* = 7.6 Hz, 1H), 4.31 (*dd*, *J* = 15.3, 7.5 Hz, 1H), 3.70 (*dd*, *J* = 15.4, 7.8 Hz, 1H). ^13^C{^1^H} NMR (100 MHz, CDCl_3_) δ 188.1, 157.3, 143.6, 142.2, 141.8, 139.2, 138.0, 129.7, 129.7, 129.2, 129.0, 128.8, 127.7, 127.5, 127.2, 127.0, 125.8, 125.2, 125.0, 120.7, 119.4, 117.6, 117.5, 117.0, 112.7, 40.9, 36.1. Enantiomeric ratio established by HPLC analysis using a Chiralpak IG-3 column, er > 99:1 (HPLC: IG-3 column, λ 278 nm, *n*-hexane/ethanol = 70:30, flow rate 1.0 mL min^–1^, 25 °C, tr (peak 1) = 16.513 min and tr (peak 2) = 33.366 min. HRMS *m*/*z* calculated for C_31_H_25_N_4_O_2_ [M + H]^+^ = 485.1978. Found: 485.1978.

#### (*R*)-3-(6-Methyl-2-phenylimidazo[1,2-*a*]pyridin-3-yl)-3-phenyl-1-(1-phenyl-1*H*-imidazol-2-yl)propan-1-one **(*R*)-3ah**

Yellow oil obtained by column chromatography on silica gel using hexane and ethyl acetate (50:50–30:70) as eluent with 90% yield (108.9 mg). Chemical formula: C_32_H_26_N_4_O. [α]_D_^20^ + 108.1 (*c* = 0.0011, CH_2_Cl_2_). ^1^H NMR (400 MHz, Chloroform-*d*) δ 7.53–7.46 (*m*, 4H), 7.31–7.15 (*m*, 12H), 6.98–6.91 (*m*, 4H), 5.59 (*t*, *J* = 7.7 Hz, 1H), 4.14 (*dd*, *J* = 15.9, 7.3 Hz, 1H), 3.71 (*dd*, *J* = 15.9, 8.4 Hz, 1H), 2.13 (*s*, 3H). ^13^C{^1^H} NMR (100 MHz, CDCl_3_) δ 188.3, 144.1, 142.2, 140.1, 138.1, 134.7, 129.6, 129.0, 129.0, 128.9, 128.8, 128.4, 127.8, 127.2, 127.1, 127.0, 125.7, 122.4, 121.7, 120.5, 116.9, 41.9, 36.0, 18.7. Enantiomeric ratio established by HPLC analysis using a Chiralpak IG-3 column, er > 99:1 (HPLC: IG-3 column, λ 278 nm, *n*-hexane/ethanol = 70:30, flow rate 1.0 mL min^–1^, 25 °C, tr (peak 1) = 9.448 min and tr (peak 2) = 14.264 min. HRMS *m*/*z* calculated for C_32_H_27_N_4_O [M + H]^+^ = 483.2185. Found: 483.2189.

#### (*R*)-3-(2-(4-Methoxyphenyl)-7-methylimidazo[1,2-*a*]pyridin-3-yl)-3-phenyl-1-(1-phenyl-1*H*-imidazol-2-yl)propan-1-one **(*R*)-3ai**

Yellow solid obtained by column chromatography on silica gel using hexane and ethyl acetate (50:50–40:60) as eluent with 83% yield (107.0 mg). Melting point: 165–169 °C. Chemical formula: C_33_H_28_N_4_O_2_. [α]_D_^20^ + 132.2 (*c* = 0.0008, CH_2_Cl_2_). ^1^H NMR (400 MHz, Chloroform-*d*) δ 7.57 (*d*, *J* = 7.0 Hz, 1H), 7.47–7.42 (*m*, 2H), 7.31–7.26 (*m*, 4H), 7.24–7.13 (*m*, 5H), 6.99–6.91 (*m*, 4H), 6.81–6.76 (*m*, 2H), 6.39 (*dd*, *J* = 7.0, 1.6 Hz, 1H), 5.55 (*t*, *J* = 7.8 Hz, 1H), 4.12 (*dd*, *J* = 15.7, 7.4 Hz, 1H), 3.80–3.65 (*m*, 4H), 2.27 (*s*, 3H). ^13^C{^1^H} NMR (100 MHz, CDCl_3_) δ 188.3, 159.3, 145.5, 143.8, 142.2, 140.2, 138.1, 135.2, 130.1, 129.6, 129.0, 128.8, 127.3, 127.3, 127.2, 127.1, 127.0, 125.7, 123.9, 119.5, 115.8, 114.6, 113.9, 55.4, 41.8, 36.0, 21.3. Enantiomeric ratio established by HPLC analysis using a Chiralpak IG-3 column, er > 99:1 (HPLC: IG-3 column, λ 278 nm, *n*-hexane/ethanol = 60:40, flow rate 1.0 mL min^–1^, 25 °C, tr (peak 1) = 14.290 min and tr (peak 2) = 35.354 min. HRMS *m*/*z* calculated for C_33_H_29_N_4_O_2_ [M + H]^+^ = 513.2291. Found: 513.2291.

#### (*R*)-3-Phenyl-1-(1-phenyl-1*H*-imidazol-2-yl)-3-(2-phenylimidazo[1,2-*a*]pyrimidin-3-yl)propan-1-one **(*R*)-3aj**

White solid obtained by column chromatography on silica gel using hexane and ethyl acetate (50:50–10:90) as eluent with 73% yield (85.8 mg). Melting point: 117–120 °C. Chemical formula: C_30_H_23_N_5_O. [α]_D_^20^ + 196.7 (*c* = 0.0008, CH_2_Cl_2_). ^1^H NMR (400 MHz, Chloroform-*d*) δ 8.43 (*dd*, *J* = 4.1, 1.9 Hz, 1H), 8.03 (*dd*, *J* = 6.9, 1.9 Hz, 1H), 7.63 (*d*, *J* = 6.9 Hz, 2H), 7.35–7.13 (*m*, 12H), 6.96 (*dd*, *J* = 8.2, 5.6 Hz, 3H), 6.64 (*dd*, *J* = 6.9, 4.1 Hz, 1H), 5.65 (*t*, *J* = 7.9 Hz, 1H), 4.11 (*dd*, *J* = 15.4, 7.4 Hz, 1H), 3.77 (*dd*, *J* = 15.4, 8.6 Hz, 1H). ^13^C{^1^H} NMR (100 MHz, CDCl_3_) δ 188.0, 149.6, 148.2, 145.9, 142.1, 139.1, 137.9, 133.9, 132.4, 129.7, 129.3, 129.1, 129.1, 128.9, 128.5, 128.3, 127.6, 127.4, 127.0, 125.8, 119.4, 108.2, 41.1, 36.0. Enantiomeric ratio established by HPLC analysis using a Chiralpak IG-3 column, er > 99:1 (HPLC: IG-3 column, λ 278 nm, *n*-hexane/ethanol = 70:30, flow rate 1.0 mL min^–1^, 25 °C, tr (peak 1) = 25.655 min and tr (peak 2) = 35.399 min. HRMS *m*/*z* calculated for C_30_H_24_N_5_O [M + H]^+^ = 470.1981. Found: 470.1981.

#### (*R*)-3-(2-(4-Chlorophenyl)imidazo[1,2-*a*]pyrimidin-3-yl)-3-phenyl-1-(1-phenyl-1*H*-imidazol-2-yl)propan-1-one **(*R*)-3ak**

Yellow solid obtained by column chromatography on silica gel using hexane and ethyl acetate (70:30–10:90) as eluent with 70% yield (55.3 mg). Melting point: 109–110 °C. Chemical formula: C_30_H_22_ClN_5_O. [α]_D_^20^ + 113.9 (*c* = 0.0019, CH_2_Cl_2_). ^1^H NMR (400 MHz, Chloroform-*d*) δ 8.42 (*dd*, *J* = 4.1, 1.9 Hz, 1H), 8.03 (*dd*, *J* = 6.9, 1.9 Hz, 1H), 7.56 (*d*, *J* = 8.5 Hz, 2H), 7.31–7.17 (*m*, 8H), 7.13 (*d*, *J* = 7.7 Hz, 2H), 7.00–6.97 (*m*, 2H), 6.94 (*dt*, *J* = 6.6, 1.4 Hz, 2H), 6.64 (*dd*, *J* = 6.9, 4.1 Hz, 1H), 5.58 (*t*, *J* = 8.0 Hz, 1H), 4.05 (*dd*, *J* = 15.4, 7.2 Hz, 1H), 3.80 (*dd*, *J* = 15.4, 8.9 Hz, 1H). ^13^C{^1^H} NMR (100 MHz, CDCl_3_) δ 187.8, 149.8, 148.1, 147.2, 144.6, 142.0, 138.9, 137.8, 134.3, 132.5, 132.4, 130.3, 129.7, 129.3, 129.0, 129.0, 128.7, 127.6, 127.5, 126.9, 125.7, 119.6, 108.4, 41.0, 35.9. Enantiomeric ratio established by HPLC analysis using a Chiralpak IG-3 column, er > 99:1 (HPLC: IG-3 column, λ 278 nm, *n*-hexane/ethanol = 70:30, flow rate 1.0 mL min^–1^, 25 °C, tr (peak 1) = 27.488 min and tr (peak 2) = 34.367 min. HRMS *m*/*z* calculated for C_30_H_23_ClN_5_O [M + H]^+^ = 504.1591. Found: 504.1591.

#### (*R*)-3-(2-Methyl-6-phenylimidazo[2,1-β]thiazol-5-yl)-3-phenyl-1-(1-phenyl-1*H*-imidazol-2-yl)propan-1-one **(*R*)-3al**

White solid obtained by column chromatography on silica gel using hexane and ethyl acetate (70:30–50:50) as eluent with 65% yield (79.0 mg). Melting point: 156–160 °C. Chemical formula: C_30_H_24_N_4_OS. [α]_D_^20^ + 140.3 (*c* = 0.0003, CH_2_Cl_2_). ^1^H NMR (400 MHz, Chloroform-*d*) δ 7.52–7.48 (*m*, 2H), 7.33–7.15 (*m*, 11H), 7.02 (*d*, *J* = 1.0 Hz, 1H), 6.99 (*d*, *J* = 1.0 Hz, 1H), 6.97–6.92 (*m*, 2H), 6.79 (*d*, *J* = 1.4 Hz, 1H), 5.48 (*t*, *J* = 7.9 Hz, 1H), 4.14 (*dd*, *J* = 15.4, 7.9 Hz, 1H), 3.53 (*dd*, *J* = 15.4, 8.0 Hz, 1H), 2.25 (*d*, *J* = 1.4 Hz, 3H). ^13^C{^1^H} NMR (100 MHz, CDCl_3_) δ 188.3, 148.9, 143.5, 142.4, 140.6, 138.0, 134.7, 129.6, 129.1, 128.9, 128.8, 128.5, 128.1, 127.3, 127.2, 127.2, 127.0, 125.9, 125.7, 123.0, 115.0, 42.6, 36.6, 14.3. Enantiomeric ratio established by HPLC analysis using a Chiralpak IG-3 column, er > 99:1 (HPLC: IG-3 column, λ 278 nm, *n*-hexane/2-propanol = 80:20, flow rate 1.0 mL min^–1^, 25 °C, tr (peak 1) = 19.948 min and tr (peak 2) = 26.159 min. HRMS *m*/*z* calculated for C_30_H_25_N_4_OS [M + H]^+^ = 489.1749. Found: 489.1749.

### General Procedure for the Preparation of Compound **(*R*)-4aa**(22)

Product **(*****R*****)-3aa** (0.1 mmol) and methanol (0.4 mL) were placed in a 10 mL Schlenk tube equipped with a magnetic stir bar. Then, the flask was closed with a screw cap, and the reaction mixture was stirred for 24 h at 120 °C. After this period, the reaction mixture was cooled to room temperature and then concentrated under vacuum in a rotatory evaporator. The crude product was purified by silica gel column chromatography with a mixture of hexane/ethyl acetate.

#### Methyl-(*R*)-3-phenyl-3-(2-phenylimidazo[1,2-*a*]pyridin-3-yl)propanoate **(*R*)-4aa**

Yellow oil obtained by column chromatography on silica gel using hexane and ethyl acetate (70:30) as eluent with 62% yield (22.1 mg). Chemical formula: C_23_H_20_N_2_O_2_. [α]_D_^20^ + 96.7 (*c* = 0.0009, CH_2_Cl_2_). ^1^H NMR (400 MHz, Chloroform-*d*) δ 7.69 (*ddt*, *J* = 19.9, 10.0, 1.3 Hz, 4H), 7.48–7.42 (*m*, 2H), 7, 41–7.35 (*m*, 1H), 7.35–7.29 (*m*, 2H), 7.29–7.20 (*m*, 3H), 7.16 (*ddd*, *J* = 9.0, 6.7, 1.2 Hz, 1H), 6.65 (*td*, *J* = 6.8, 1.2 Hz, 1H), 5.40 (*t*, *J* = 7.7 Hz, 1H), 3.47 (*s*, 3H), 3.28 (*dd*, *J* = 15.6, 7.4 Hz, 1H), 3.07 (*dd*, *J* = 15.6, 8.0 Hz, 1H). ^13^C{^1^H} NMR (100 MHz, CDCl_3_) δ 171.8, 145.0, 144.6, 139.5, 134.9, 129.2, 128.5, 128.1, 127.3, 126.9, 124.4, 124.1, 120.4, 117.9, 112.3, 77.2, 52.0, 37.0, 36.7. Enantiomeric ratio established by HPLC analysis using a Chiralpak IG-3 column, er 96:4 (HPLC: IG-3 column, λ 278 nm, *n*-hexane/ethanol = 80:20, flow rate 1.0 mL min^–1^, 25 °C, tr (peak 1) = 7.469 min and tr (peak 2) = 8.180 min. HRMS *m*/*z* calculated for C_23_H_21_N_2_O_2_ [M + H]^+^ = 357.1603. Found: 357.1605.

### General Procedure for Crystal Growth of Product **(*S*)-3ia**

To a flask containing product **(*****S*****)-3ia** (50.0 mg) was added 1 mL of ethanol. The flask was sealed with parafilm perforated with a needle to allow slow evaporation. When the solvent was almost evaporated (about 5 days at room temperature), we could obtain a white single crystal collected for X-ray analysis.

### TEMPO Trapping Experiment

2-Acylimidazol **1a** (0.25 mmol), imidazo[1,2-*a*]pyridine **2a** (0.25 mmol), rhodium chiral catalyst Λ-RhS (0.05 mol %, 1.07 mg), TEMPO (2 equiv, 0.50 mmol, 78.12 mg), and ethanol (1 mL) were placed in a test tube equipped with a magnetic stir bar at room temperature during 4 h. Then, the reaction mixture was concentrated under vacuum in a rotatory evaporator. The crude product was purified by silica gel column chromatography with a mixture of ethyl acetate/hexane (30:90–50:50) to afford corresponding product **(*****R*****)-3aa** in 95% yield (111.6 mg) and er > 99:1.

### Inert Atmosphere Experiment

2-Acylimidazol **1a** (0.25 mmol), imidazo[1,2-*a*]pyridine **2a** (0.25 mmol), rhodium chiral catalyst Λ-RhS (0.05 mol %, 1.07 mg), and ethanol (1 mL) were placed in a dry Schlenk tube equipped with a magnetic stir bar, followed by degasification via freeze–pump–thaw for three cycles. After the mixture was thoroughly degassed, the tube was sealed and the reaction was stirred at room temperature during 4 h. Then, the reaction mixture was concentrated under vacuum in a rotatory evaporator. The crude product was purified by silica gel column chromatography with a mixture of ethyl acetate/hexane (30:90–50:50) to afford correspondent product **(*****R*****)-3aa** in 90% yield (89.8 mg) and er > 99:1.

## Data Availability

The data underlying this study are available in the published article and its online Supporting Information.

## Supporting Information Available

Experimental procedures, optimization details and analytical data (NMR, HPLC and HRMS) (PDF)
FAIR data, including the primary NMR FID files, for compounds **1d**, **1h**, **3aa**–**3ia**, and **3ab–al** (ZIP)
