# Supplementary material for: Highly Enantioselective Lewis Acid Catalyzed Conjugate Addition of Imidazo[1,2-a]pyridines to α,β-Unsaturated 2-Acylimidazoles under Mild Conditions
Source: J Org Chem. 2024 Jun 6;89(12):8500–12. doi: 10.1021/acs.joc.4c00445 (PMC11197092; doi:10.1021/acs.joc.4c00445)
Supplement: Supplementary file 1 — jo4c00445_si_001.pdf [file jo4c00445_si_001.pdf]

Supporting Information

Highly enantioselective Lewis Acid-Catalyzed Conjugate Addition of Imidazo[1,2-*a*]pyridines to α,β-Unsaturated 2-Acylimidazoles Under Mild Conditions

Maria Eduarda C. Thedy,[1] Vanessa Pereira,[1] Caio Rodrigo dos Santos,[1] Luiz Paulo A. Belli,[1] Marcelo S. Franco,[2] Adailton J. Bortoluzzi,[1] Louis P. Sandjo,[1] Antonio L. Braga,[1]* Francisco F. de Assis[1]*

[1]Department of Chemistry, Universidade Federal de Santa Catarina, Florianópolis, Santa Catarina, 88040-900, Brazil.
[2]Institute of Chemistry, University of Campinas (UNICAMP), Campinas, São Paulo, 13083-970, Brazil.

*e-mail: braga.antonio@ufsc.br
*e-mail: assis.francisco@ufsc.br

Contents:

1. ^{1}H NMR and $^{13}C\{^{1}H\}$ NMR Spectra of compounds ········· S2

2. Chiral HPLC analysis ········· S25

3. Single Crystal X-Ray Diffraction of **(*S*)-3ia** ········· S46

4. References ········· S52

1. ^{1}H NMR and ^{13}C{^{1}H} NMR Spectra of compounds

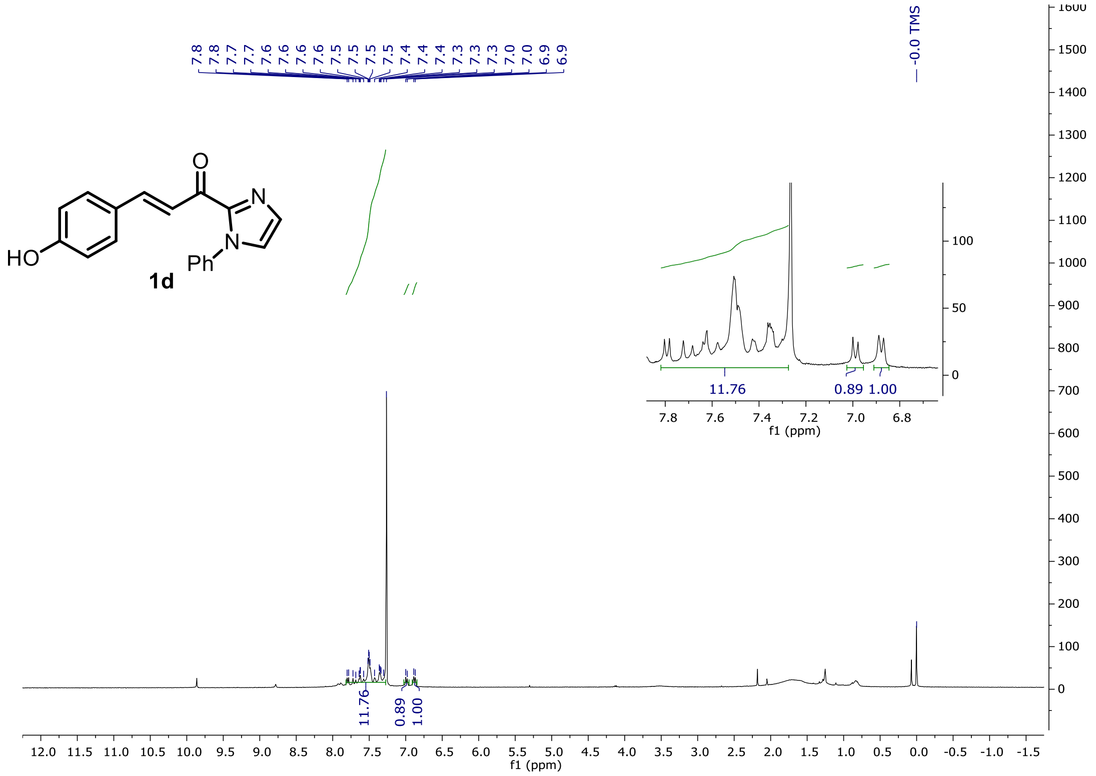

Figure S1. ^{1}H NMR (400 MHz) in $CDCl_3$ of (*E*)-3-(4-hydroxyphenyl)-1-(1-phenyl-1*H*-imidazol-2-yl)prop-2-en-1-one (**1d**).

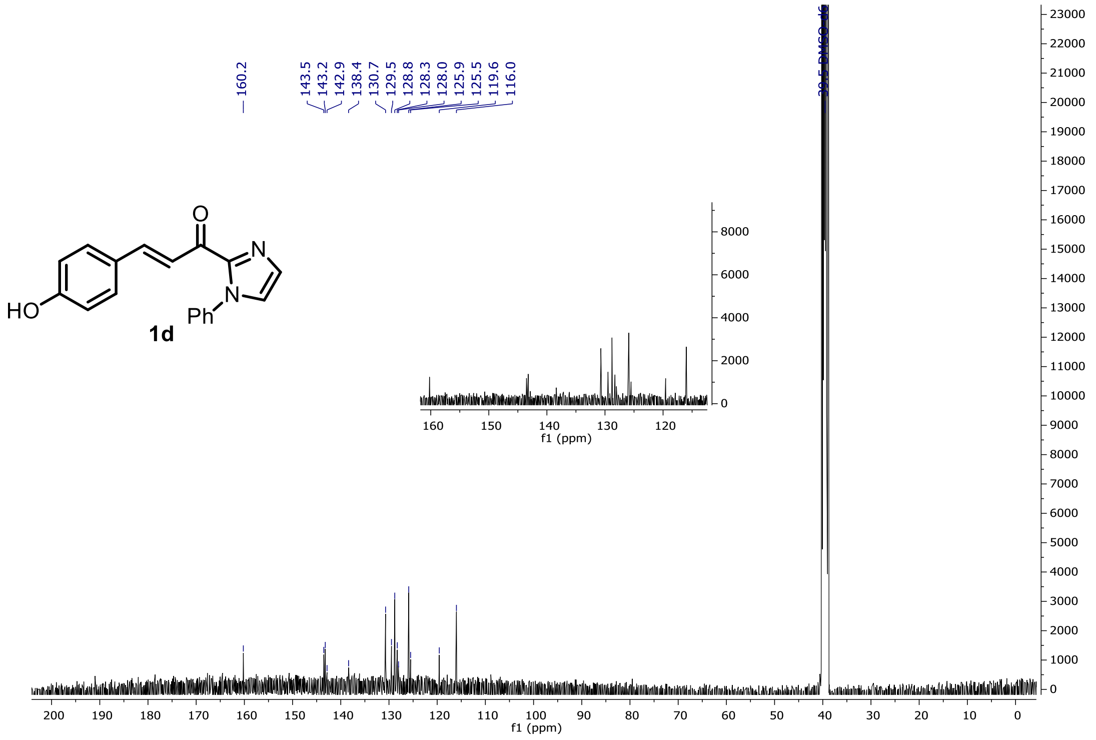

Figure S2. ^{13}C{^{1}H} NMR (100 MHz) in DMSO-d_6 of (*E*)-3-(4-hydroxyphenyl)-1-(1-phenyl-1*H*-imidazol-2-yl)prop-2-en-1-one (**1d**).

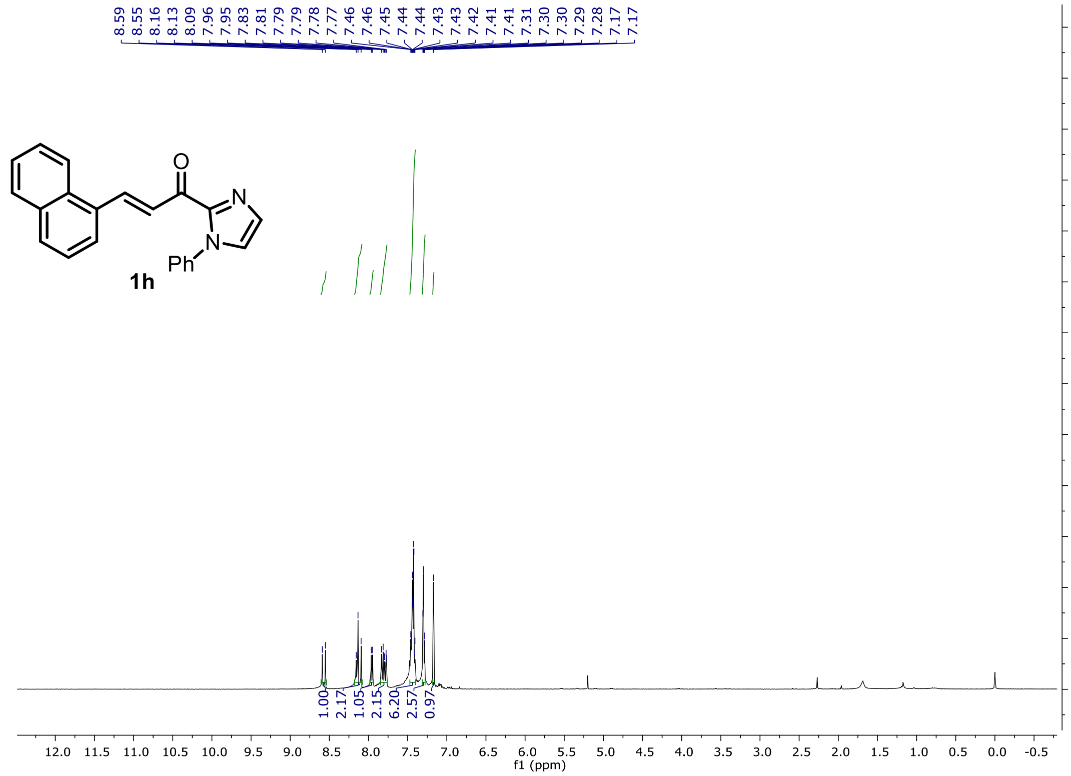

Figure S3. ^{1}H NMR (400 MHz) in $CDCl_3$ of (*E*)-3-(naphthalen-1-yl)-1-(1-phenyl-1*H*-imidazol-2-yl)prop-2-en-1-one (**1h**).

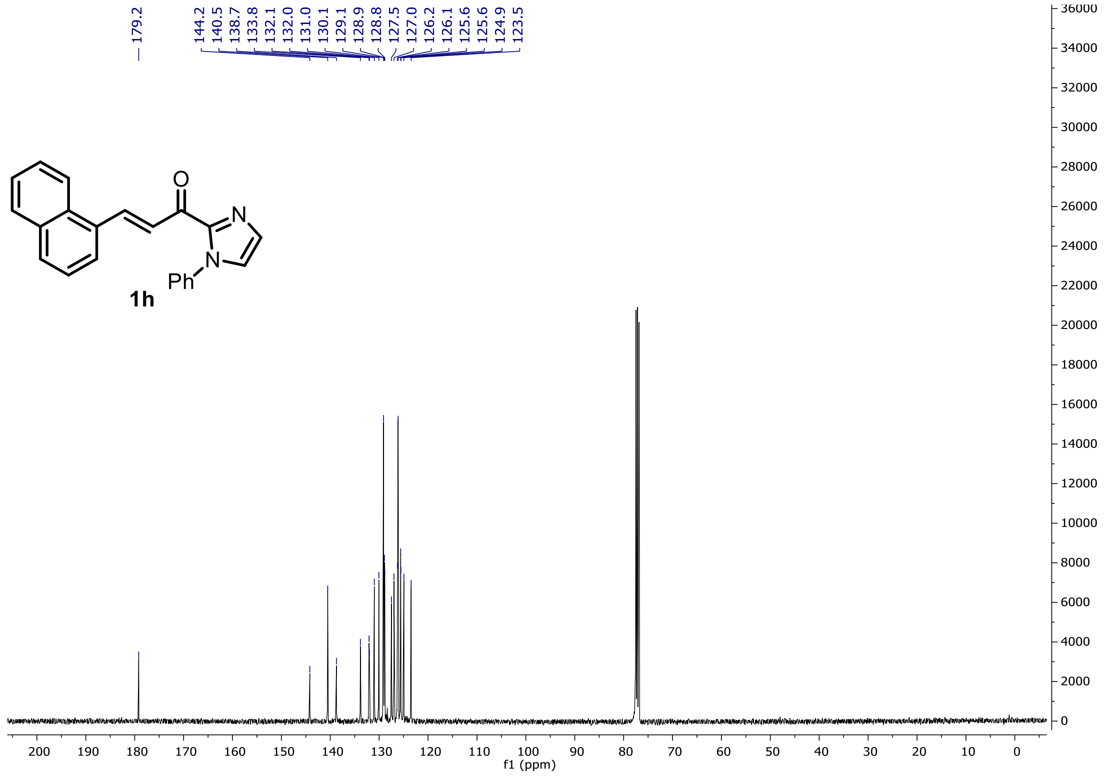

Figure S4. $^{13}C\{^{1}H\}$ NMR (100 MHz) in $CDCl_3$ of (*E*)-3-(naphthalen-1-yl)-1-(1-phenyl-1*H*-imidazol-2-yl)prop-2-en-1-one (**1h**).

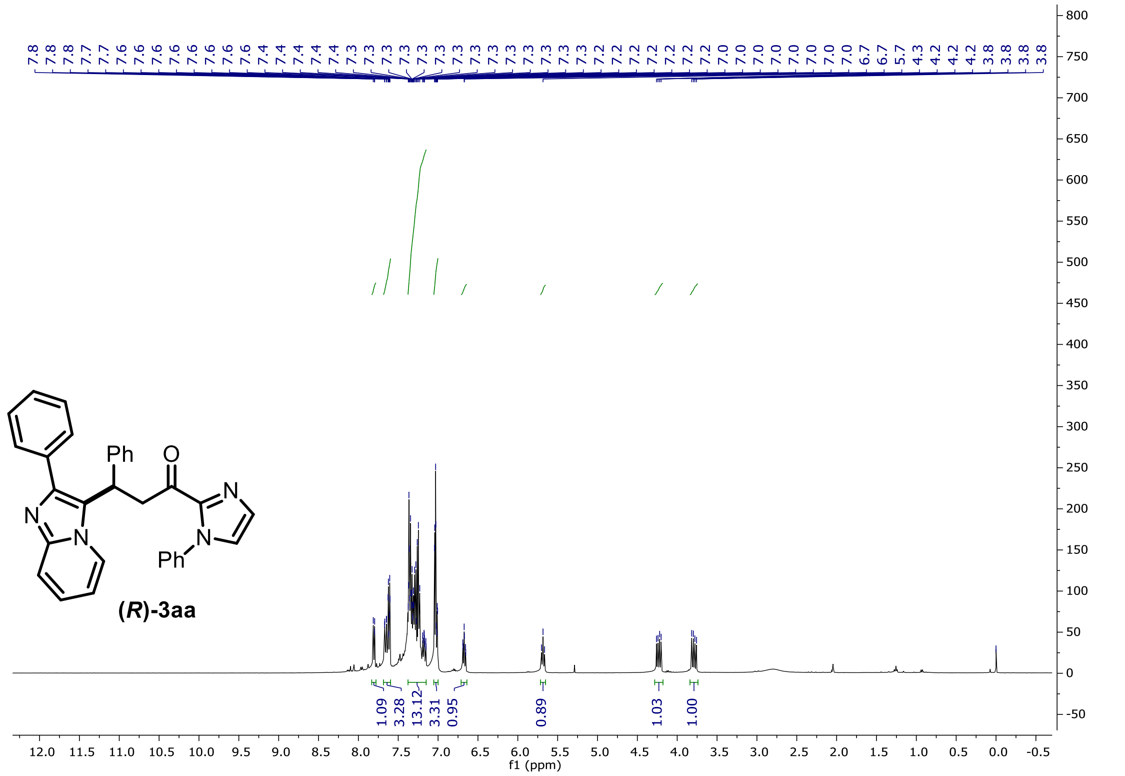

Figure S5. ^{1}H NMR (400 MHz) in $CDCl_3$ of (*R*)-3-phenyl-1-(1-phenyl-1*H*-imidazol-2-yl)-3-(2-phenylimidazo[1,2-*α*]pyridin-3-yl)propan-1-one **(*R*)-3aa**.

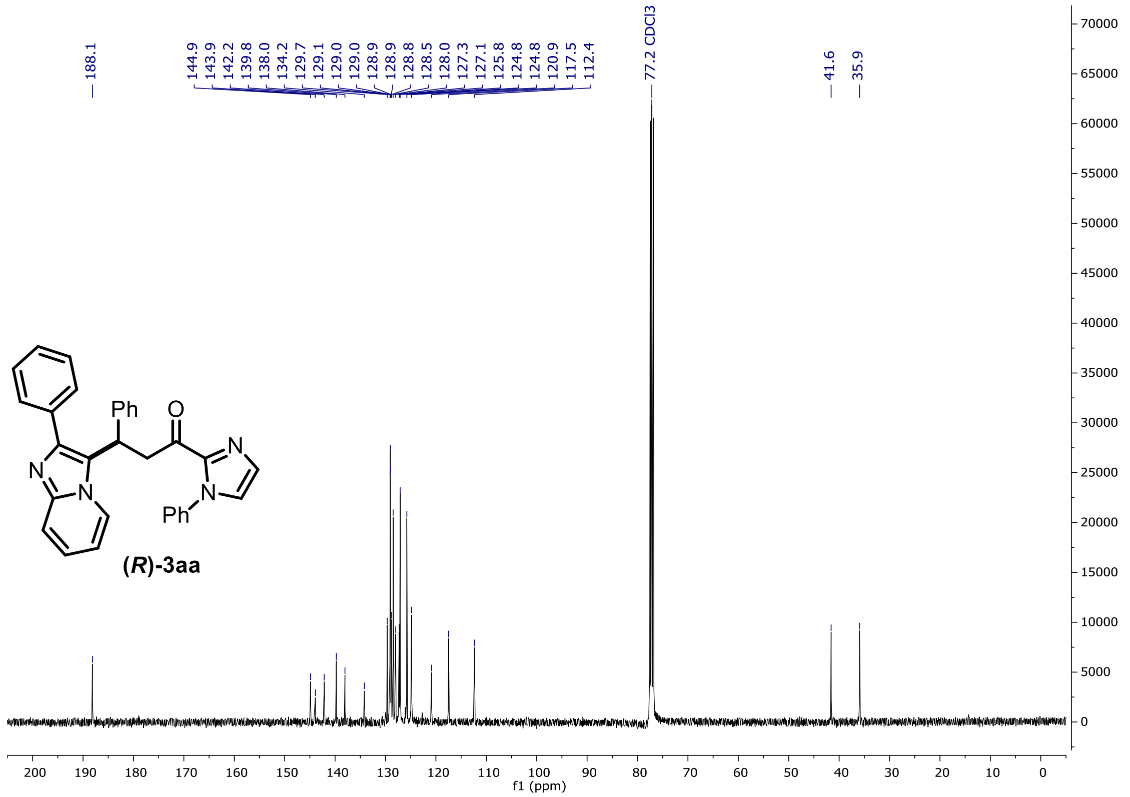

Figure S6. $^{13}C\{^{1}H\}$ NMR (100 MHz) in $CDCl_3$ of (*R*)-3-phenyl-1-(1-phenyl-1*H*-imidazol-2-yl)-3-(2-phenylimidazo[1,2-*α*]pyridin-3-yl)propan-1-one **(*R*)-3aa**.

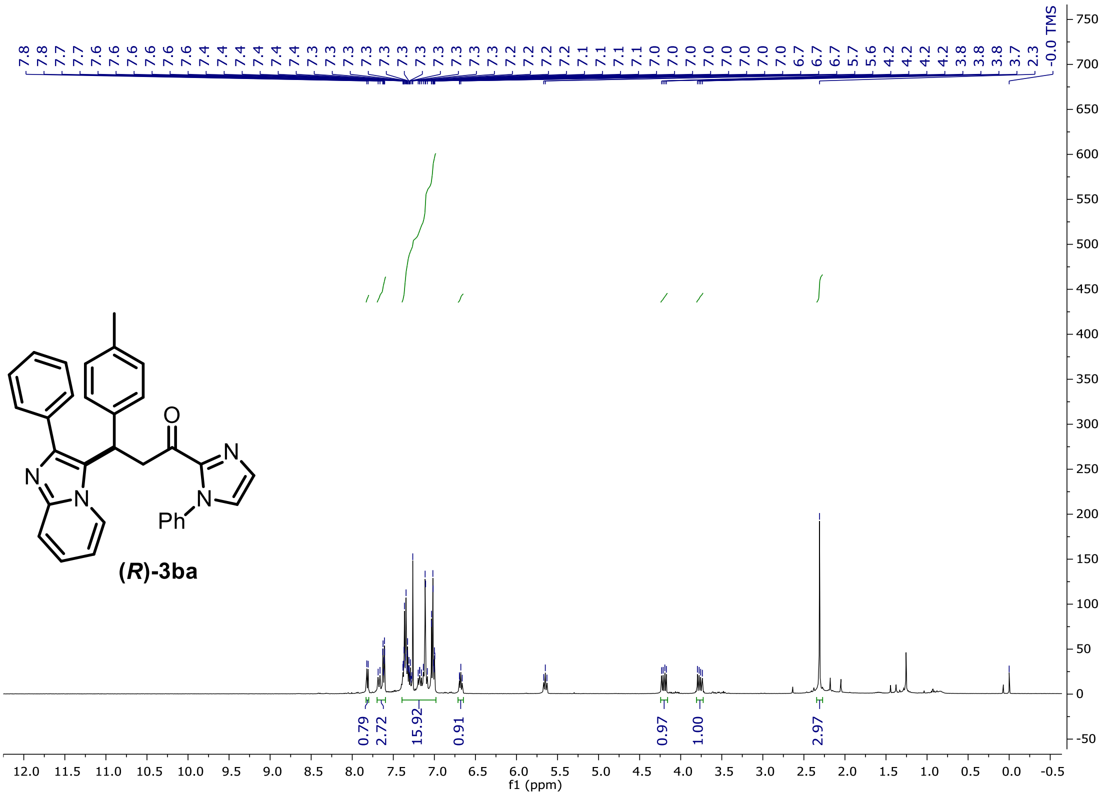

Figure S7. ^{1}H NMR (400 MHz) in $CDCl_3$ of (*R*)-1-(1-phenyl-1*H*-imidazol-2-yl)-3-(2-phenylimidazo[1,2-*α*]pyridin-3-yl)-3-(*p*-tolyl)propan-1-one **(*R*)-3ba**.

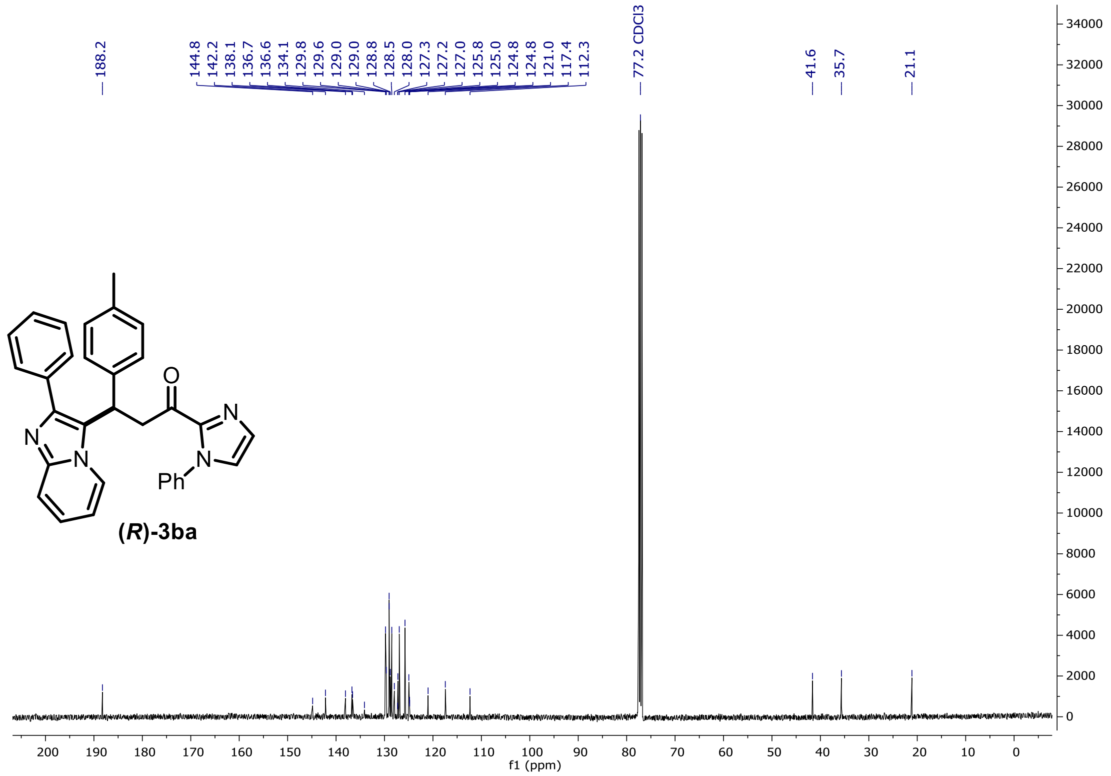

Figure S8. $^{13}C\{^{1}H\}$ NMR (100 MHz) in $CDCl_3$ of (*R*)-1-(1-phenyl-1*H*-imidazol-2-yl)-3-(2-phenylimidazo[1,2-*α*]pyridin-3-yl)-3-(*p*-tolyl)propan-1-one **(*R*)-3ba**.

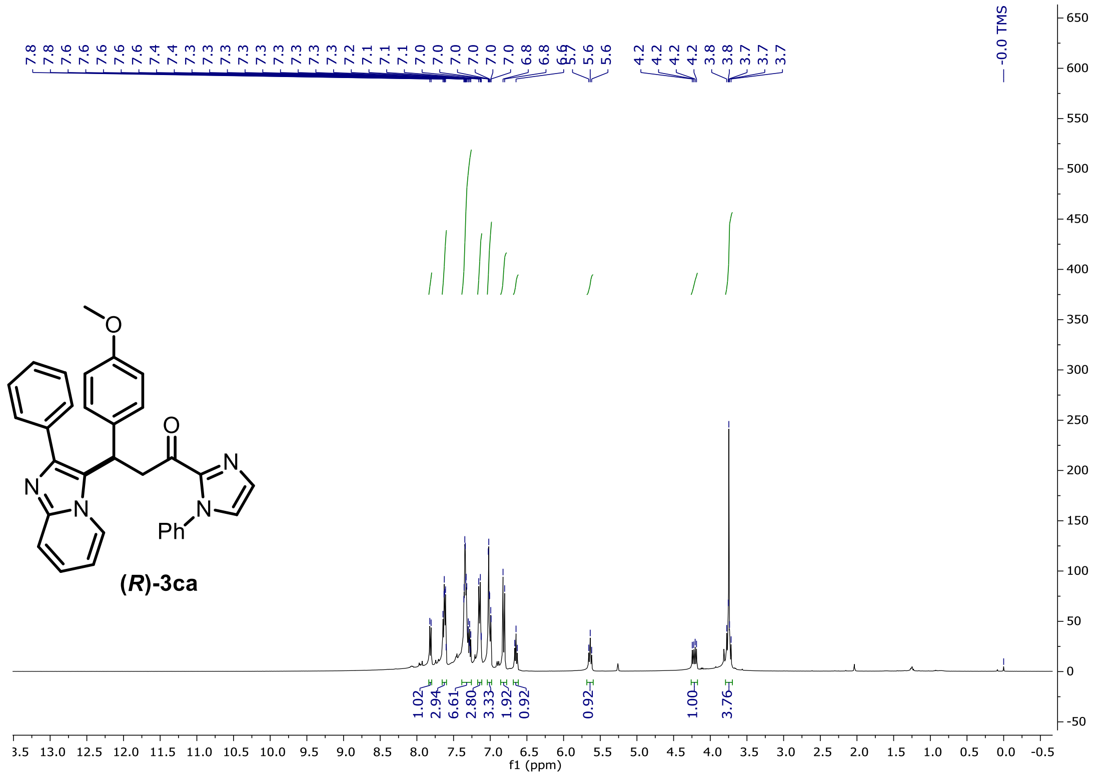

Figure S9. ^{1}H NMR (400 MHz) in $CDCl_3$ of (*R*)-3-(4-methoxyphenyl)-1-(1-phenyl-1*H*-imidazol-2-yl)-3-(2-phenylimidazo[1,2-*α*]pyridin-3-yl)propan-1-one **(*R*)-3ca**.

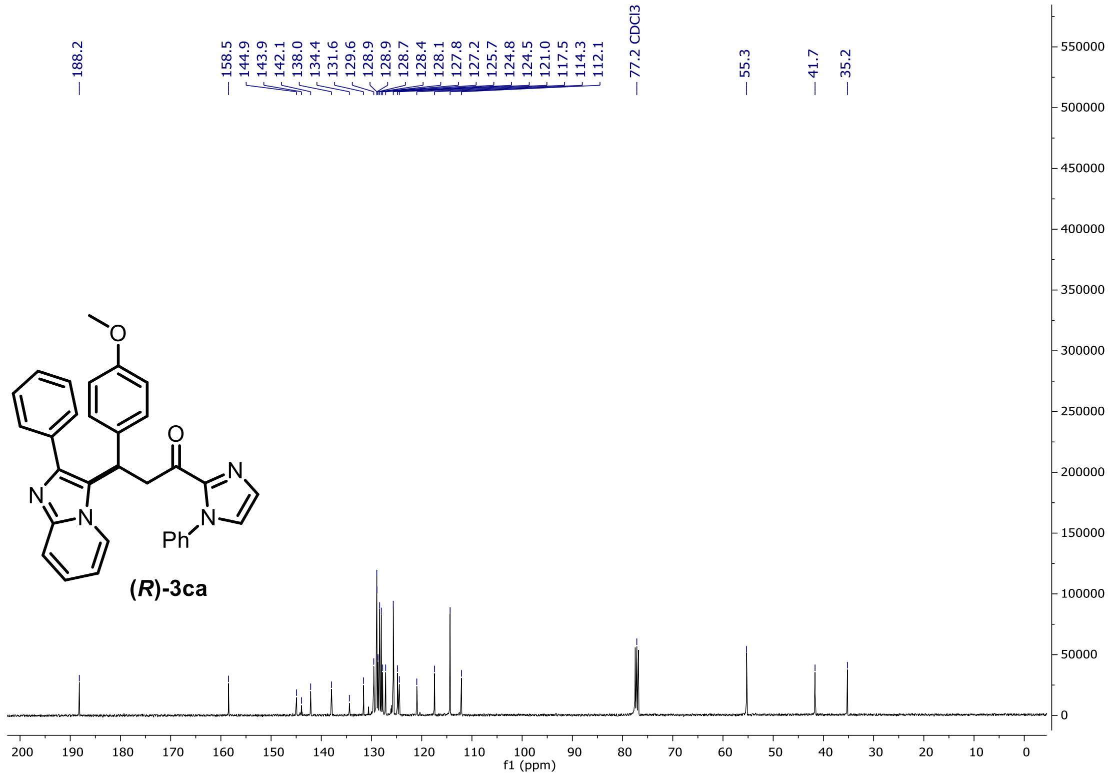

Figure S10. $^{13}C\{^{1}H\}$ NMR (100 MHz) in $CDCl_3$ of (*R*)-3-(4-methoxyphenyl)-1-(1-phenyl-1*H*-imidazol-2-yl)-3-(2-phenylimidazo[1,2-*α*]pyridin-3-yl)propan-1-one **(*R*)-3ca**.

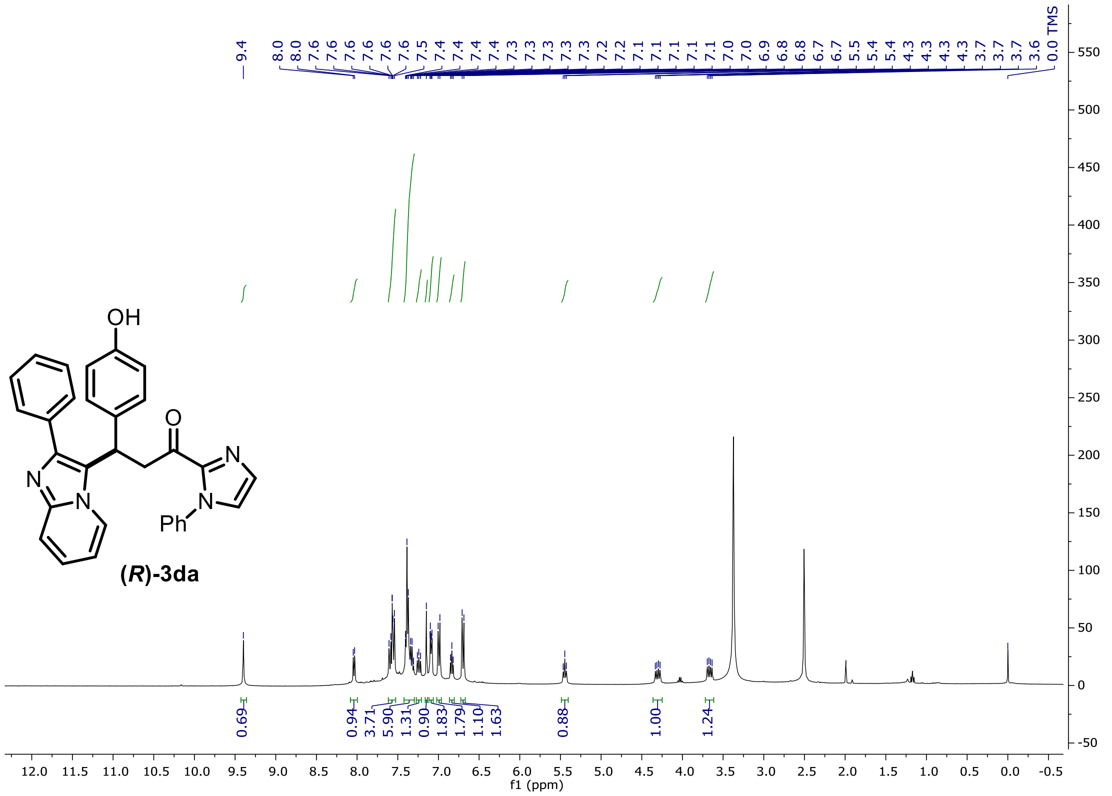

Figure S11. ^{1}H NMR (400 MHz) in DMSO-d_6 of (*R*)-3-(4-hydroxyphenyl)-1-(1-phenyl-1*H*-imidazol-2-yl)-3-(2-phenylimidazo[1,2-*α*]pyridin-3-yl)propan-1-one **(*R*)-3da**.

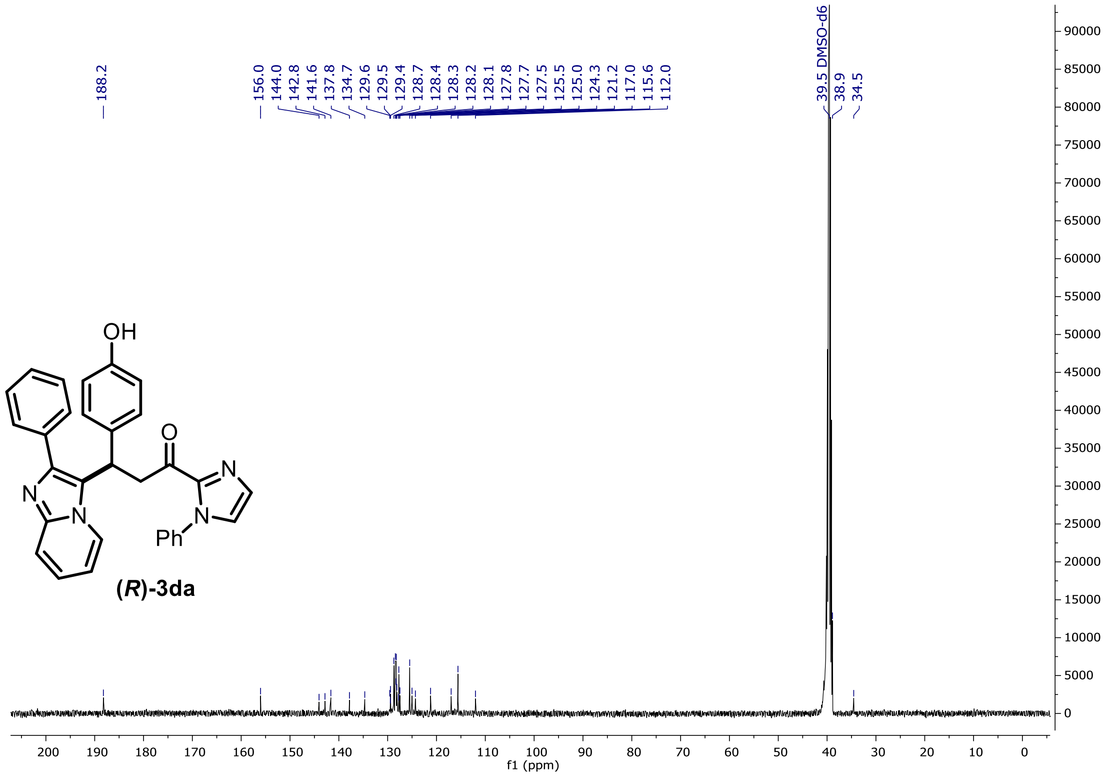

Figure S12. ^{13}C{^{1}H} NMR (100 MHz) in DMSO-d_6 of of (*R*)-3-(4-hydroxyphenyl)-1-(1-phenyl-1*H*-imidazol-2-yl)-3-(2-phenylimidazo[1,2-*α*]pyridin-3-yl)propan-1-one **(*R*)-3da**.

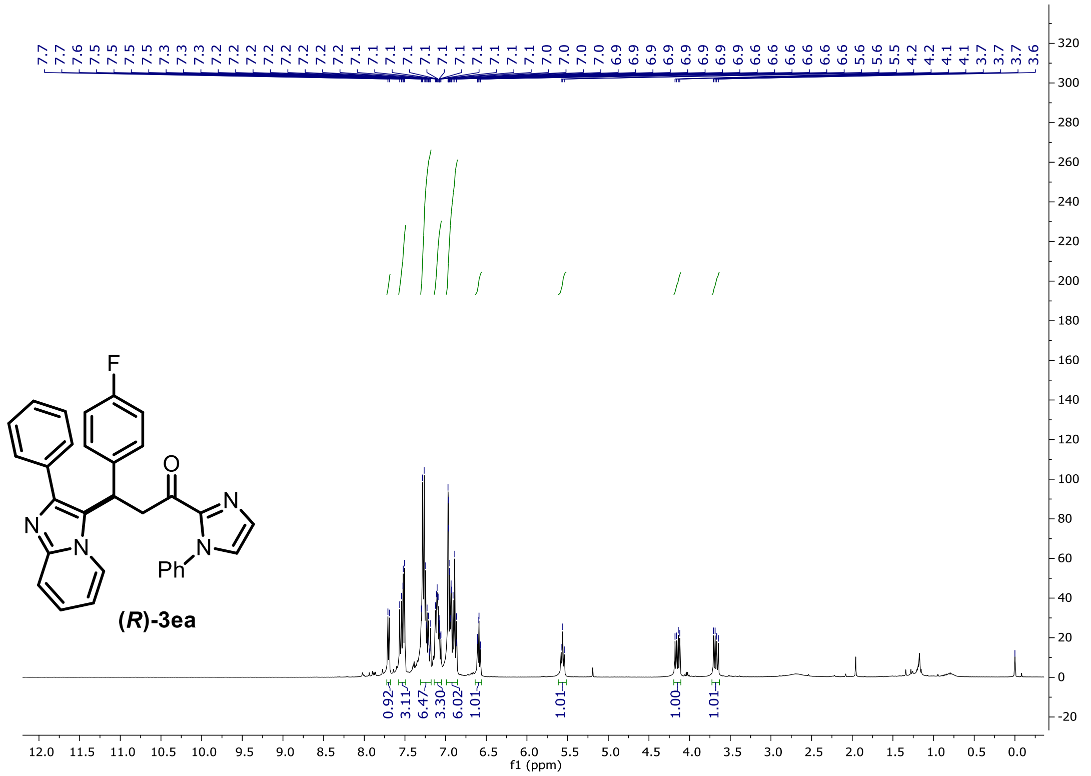

Figure S13. ^{1}H NMR (400 MHz) in $CDCl_3$ of (*R*)-3-(4-fluorophenyl)-1-(1-phenyl-1*H*-imidazol-2-yl)-3-(2-phenylimidazo[1,2-*α*]pyridin-3-yl)propan-1-one **(*R*)-3ea**.

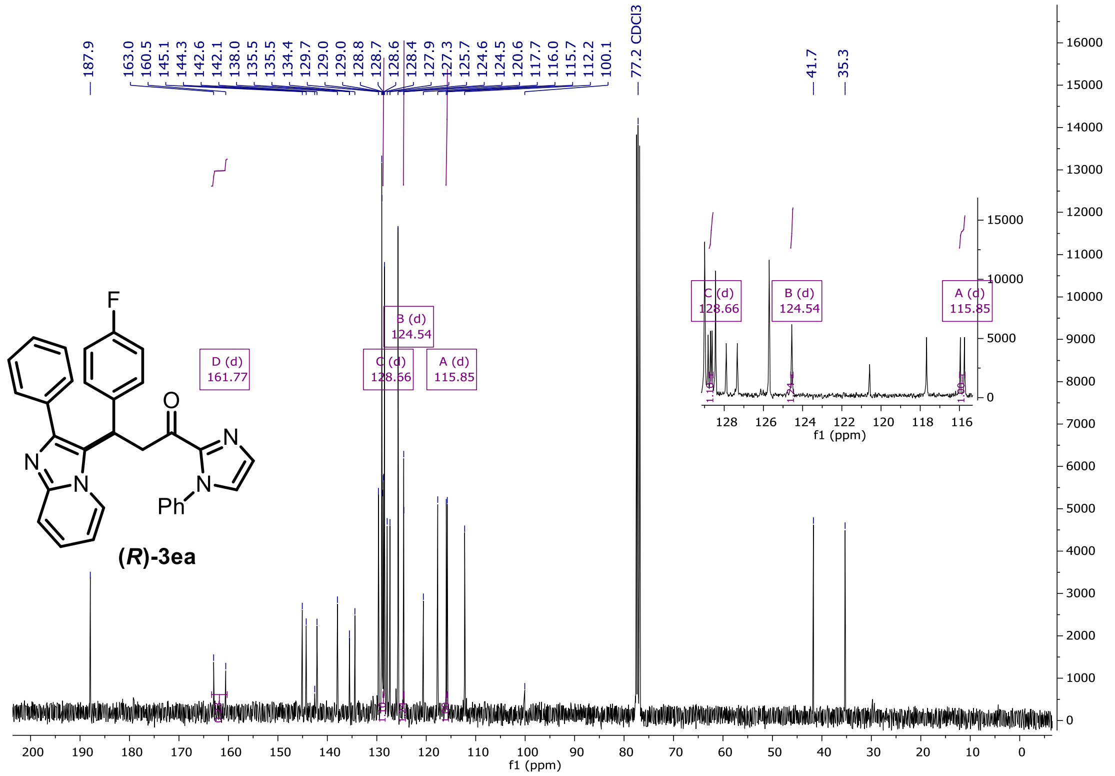

Figure S14. $^{13}C\{^{1}H\}$ NMR (100 MHz) in $CDCl_3$ of (*R*)-3-(4-fluorophenyl)-1-(1-phenyl-1*H*-imidazol-2-yl)-3-(2-phenylimidazo[1,2-*α*]pyridin-3-yl)propan-1-one **(*R*)-3ea**.

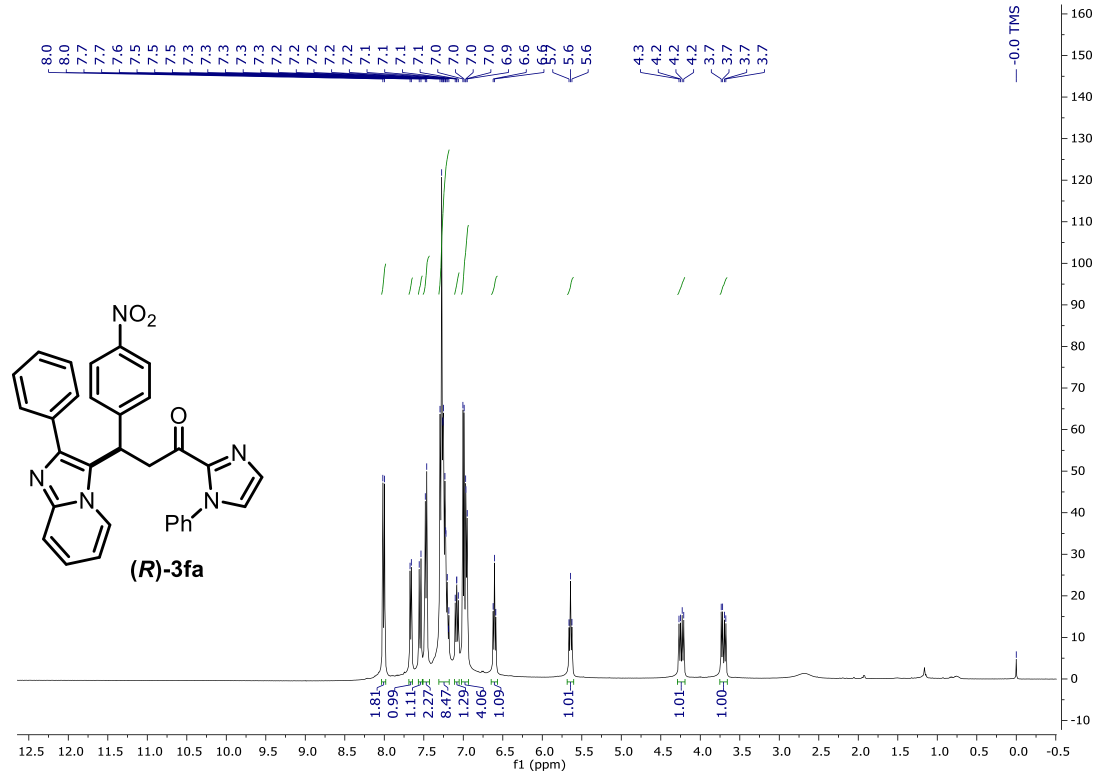

Figure S15. ^{1}H NMR (400 MHz) in $CDCl_3$ of (*R*)-3-(4-nitrophenyl)-1-(1-phenyl-1*H*-imidazol-2-yl)-3-(2-phenylimidazo[1,2-*α*]pyridin-3-yl)propan-1-one **(*R*)-3fa**.

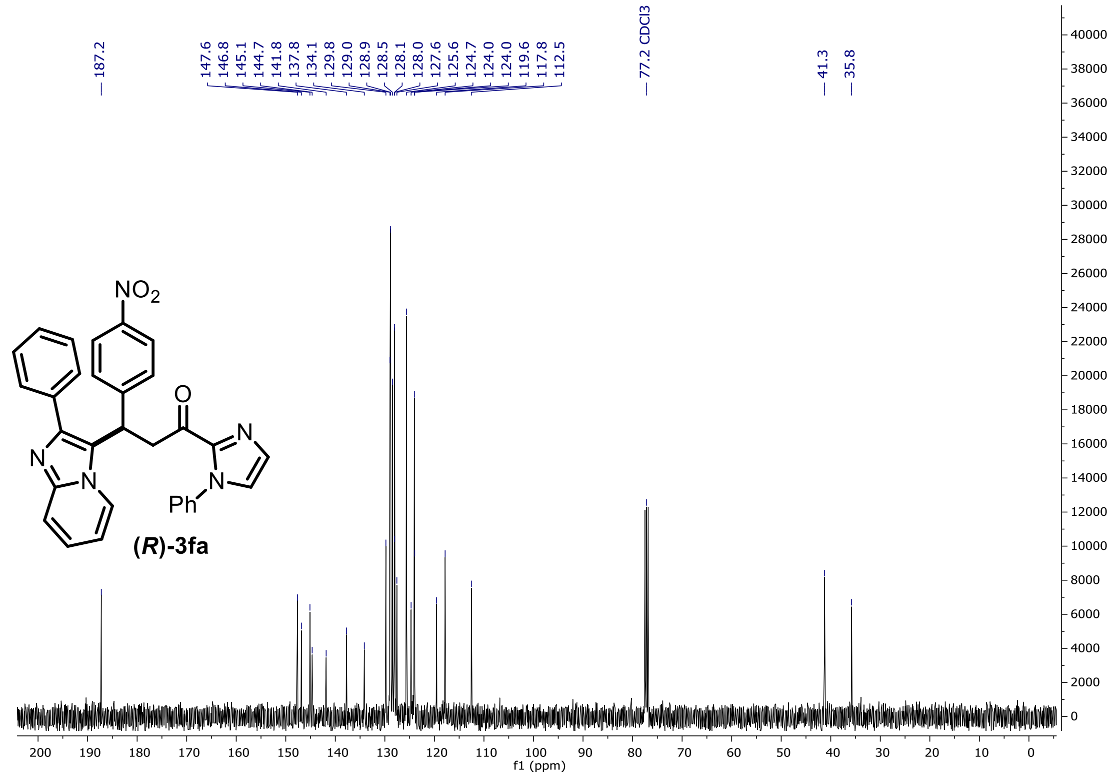

Figure S16. $^{13}C\{^{1}H\}$ NMR (100 MHz) in $CDCl_3$ of (*R*)-3-(4-nitrophenyl)-1-(1-phenyl-1*H*-imidazol-2-yl)-3-(2-phenylimidazo[1,2-*α*]pyridin-3-yl)propan-1-one **(*R*)-3fa**.

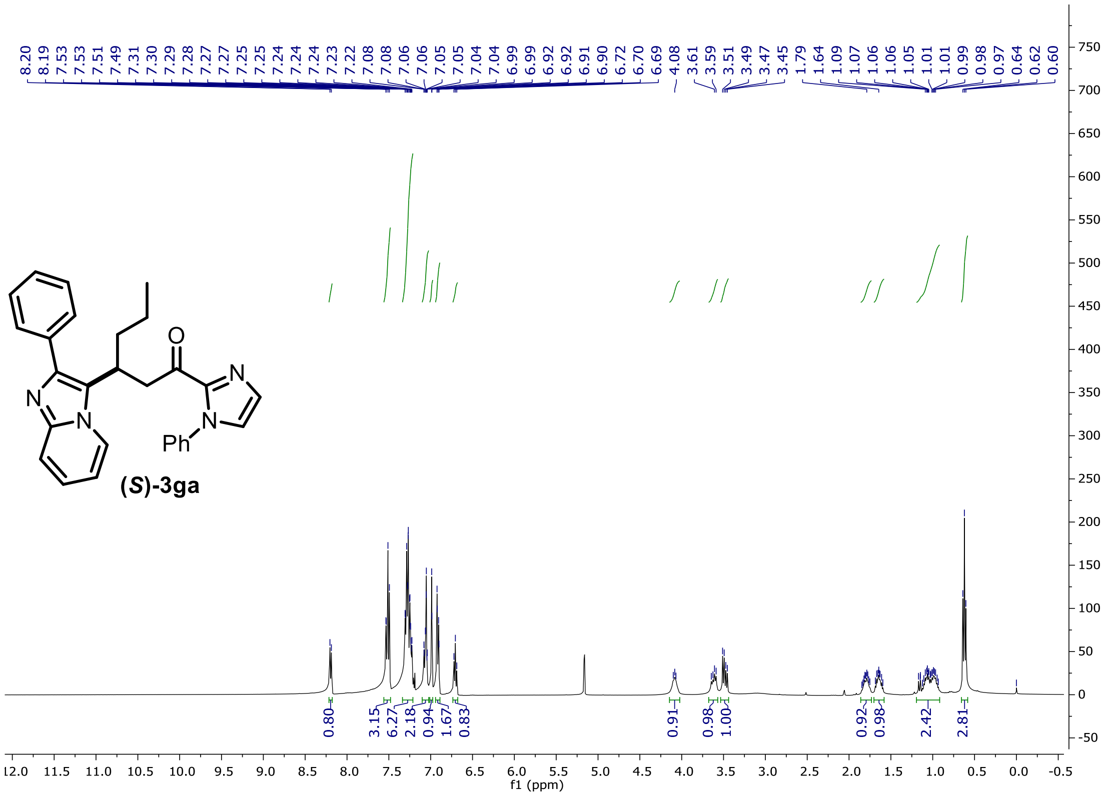

Figure S17. ^{1}H NMR (400 MHz) in $CDCl_3$ of (*S*)-1-(1-phenyl-1*H*-imidazol-2-yl)-3-(2-phenylimidazo[1,2-*α*]pyridin-3-yl)hexan-1-one **(*S*)-3ga**.

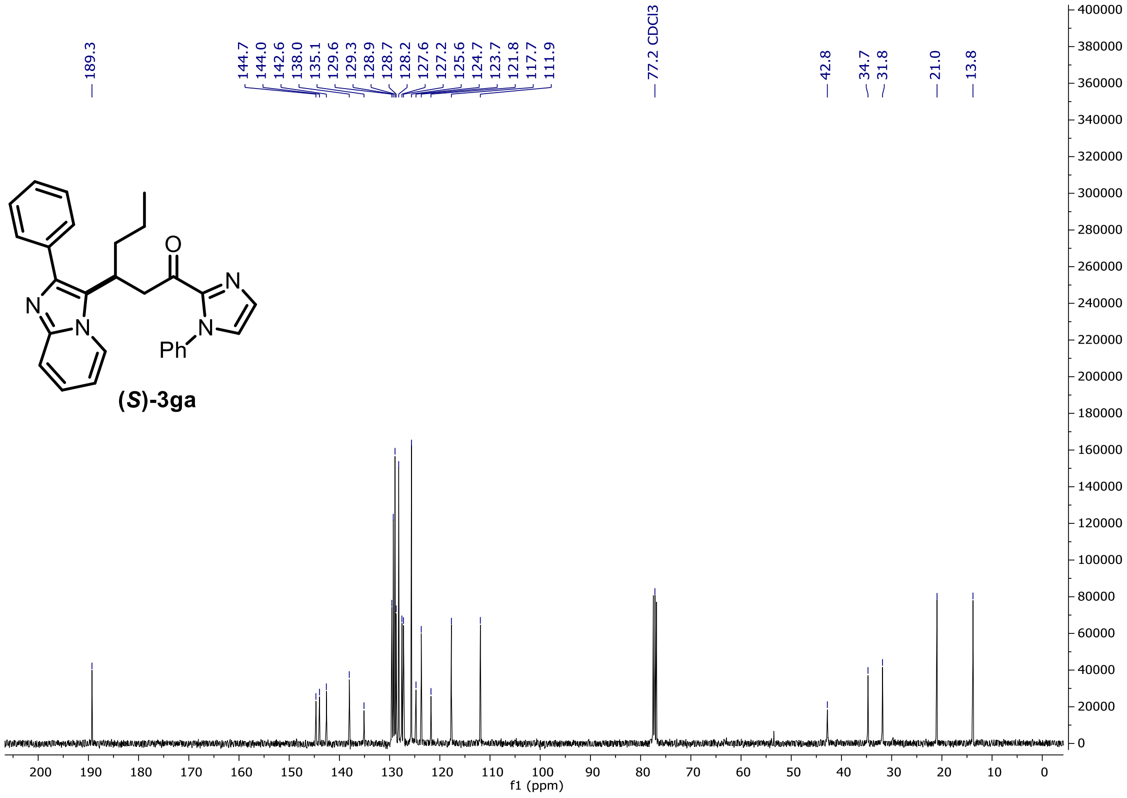

Figure S18. $^{13}C\{^{1}H\}$ NMR (100 MHz) in $CDCl_3$ of (*S*)-1-(1-phenyl-1*H*-imidazol-2-yl)-3-(2-phenylimidazo[1,2-*α*]pyridin-3-yl)hexan-1-one **(*S*)-3ga**.

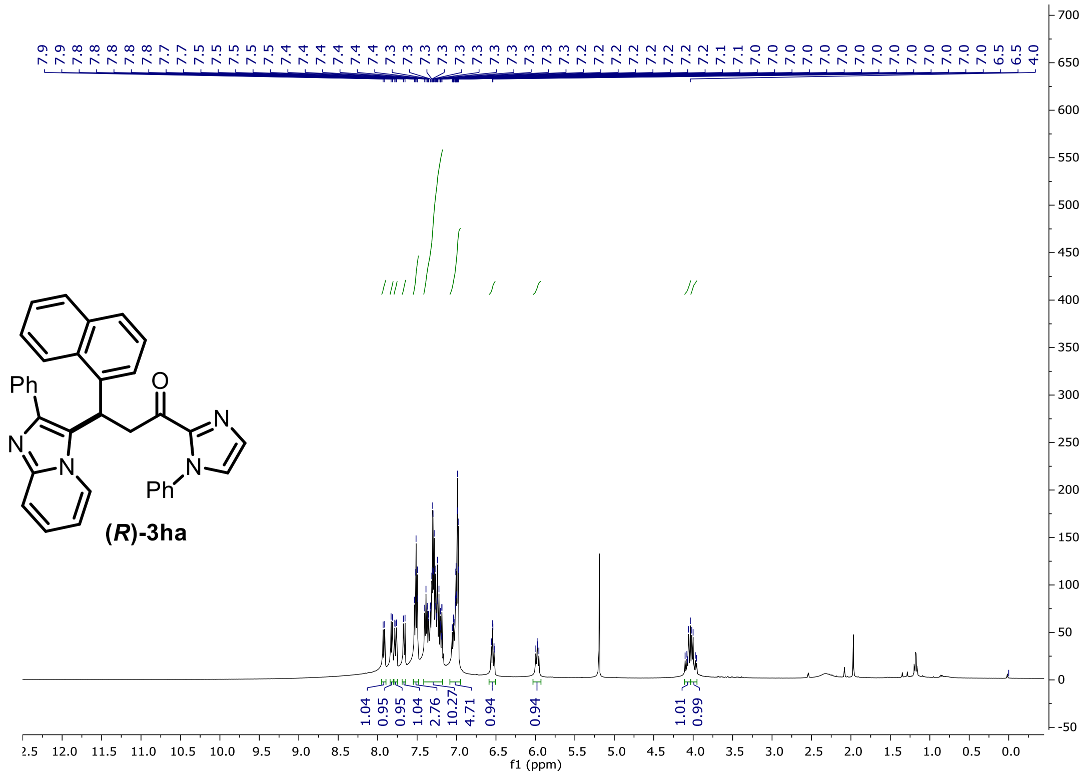

Figure S19. ^{1}H NMR (400 MHz) in $CDCl_3$ of (*R*)-3-(naphthalen-1-yl)-1-(1-phenyl-1*H*-imidazol-2-yl)-3-(2-phenylimidazo[1,2-*α*]pyridin-3-yl)propan-1-one **(*R*)-3ha**.

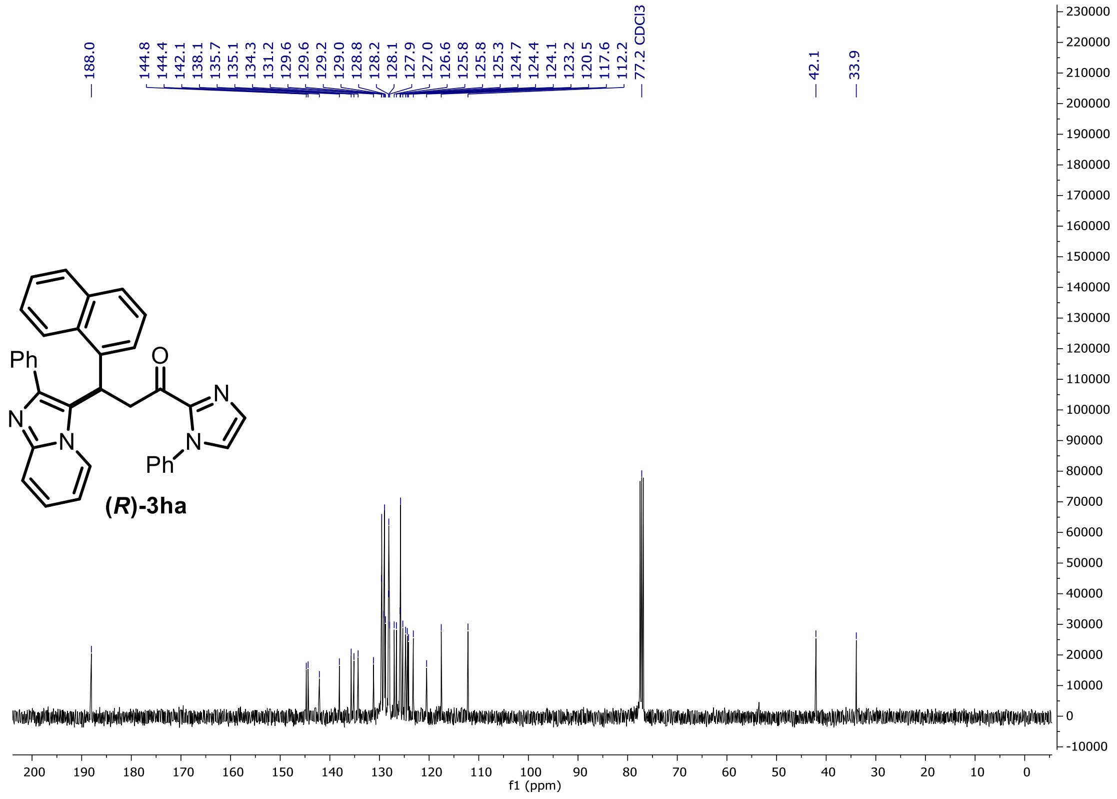

Figure S20. $^{13}C\{^{1}H\}$ NMR (100 MHz) in $CDCl_3$ of (*R*)-3-(naphthalen-1-yl)-1-(1-phenyl-1*H*-imidazol-2-yl)-3-(2-phenylimidazo[1,2-*α*]pyridin-3-yl)propan-1-one **(*R*)-3ha**.

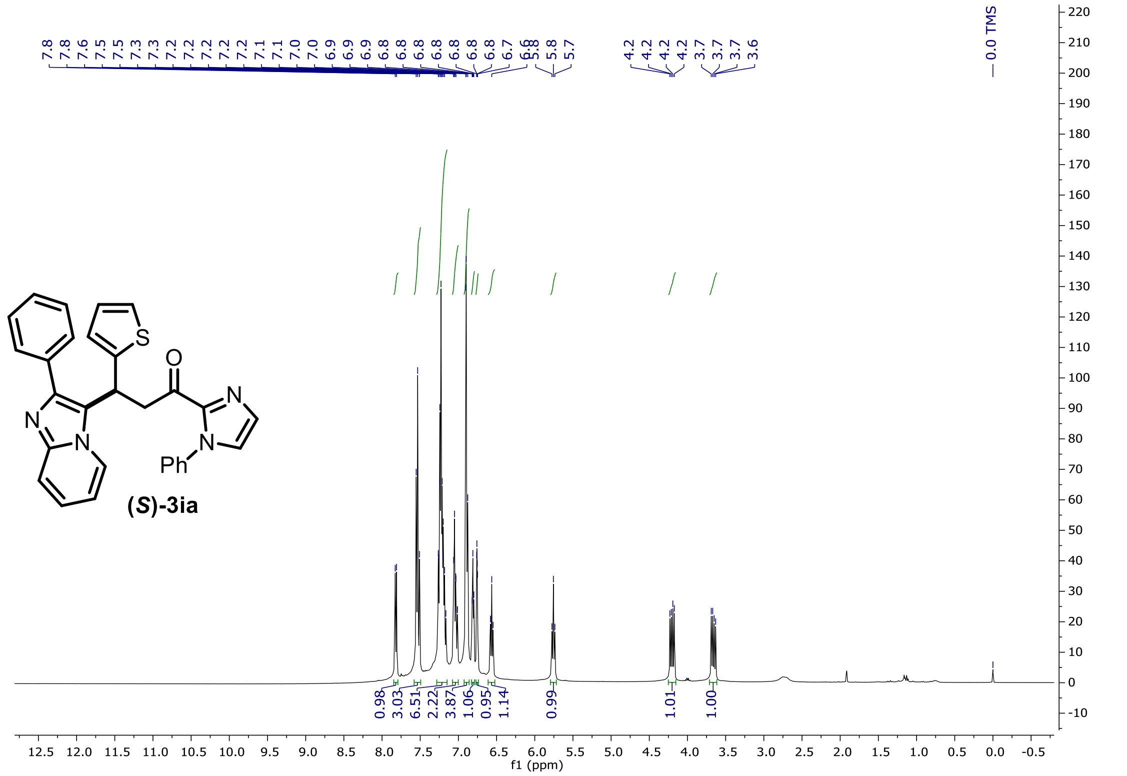

Figure S21. ^{1}H NMR (400 MHz) in $CDCl_3$ of (*S*)-1-(1-phenyl-1*H*-imidazol-2-yl)-3-(2-phenylimidazo[1,2-*α*]pyridin-3-yl)-3-(thiophen-2-yl)propan-1-one **(*S*)-3ia**.

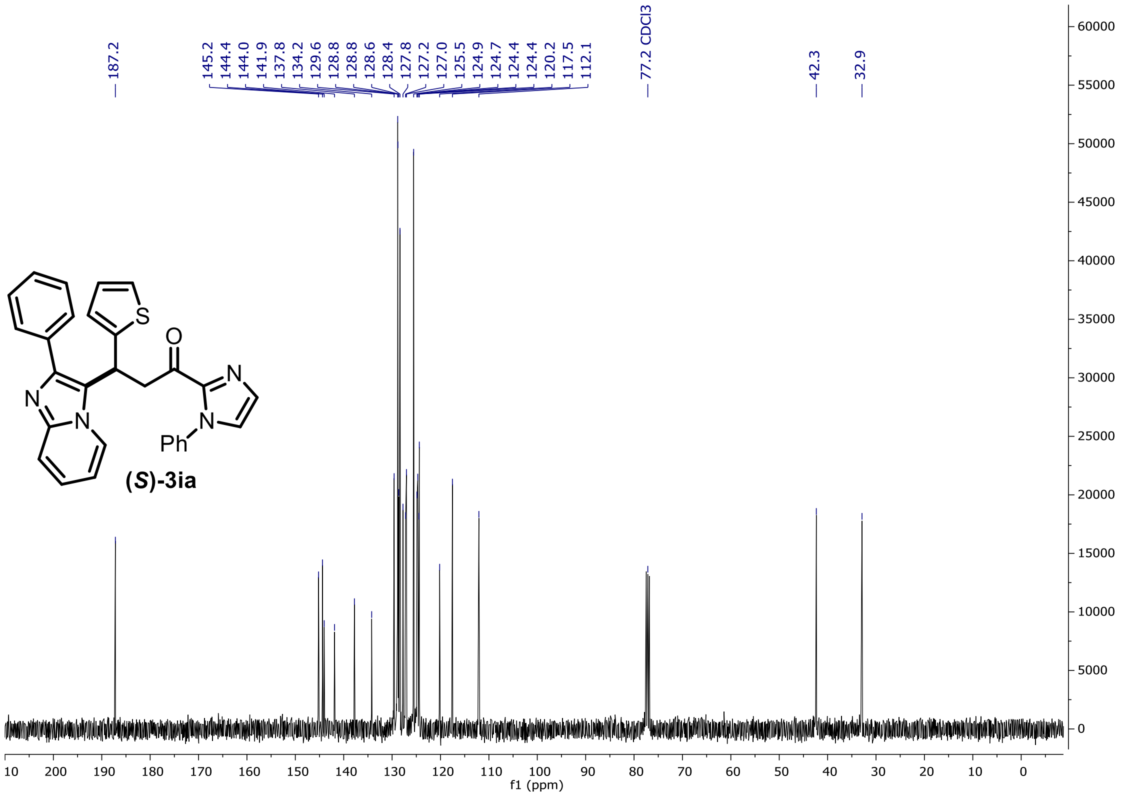

Figure S22. $^{13}C\{^{1}H\}$ NMR (100 MHz) in $CDCl_3$ of (*S*)-1-(1-phenyl-1*H*-imidazol-2-yl)-3-(2-phenylimidazo[1,2-*α*]pyridin-3-yl)-3-(thiophen-2-yl)propan-1-one **(*S*)-3ia**.

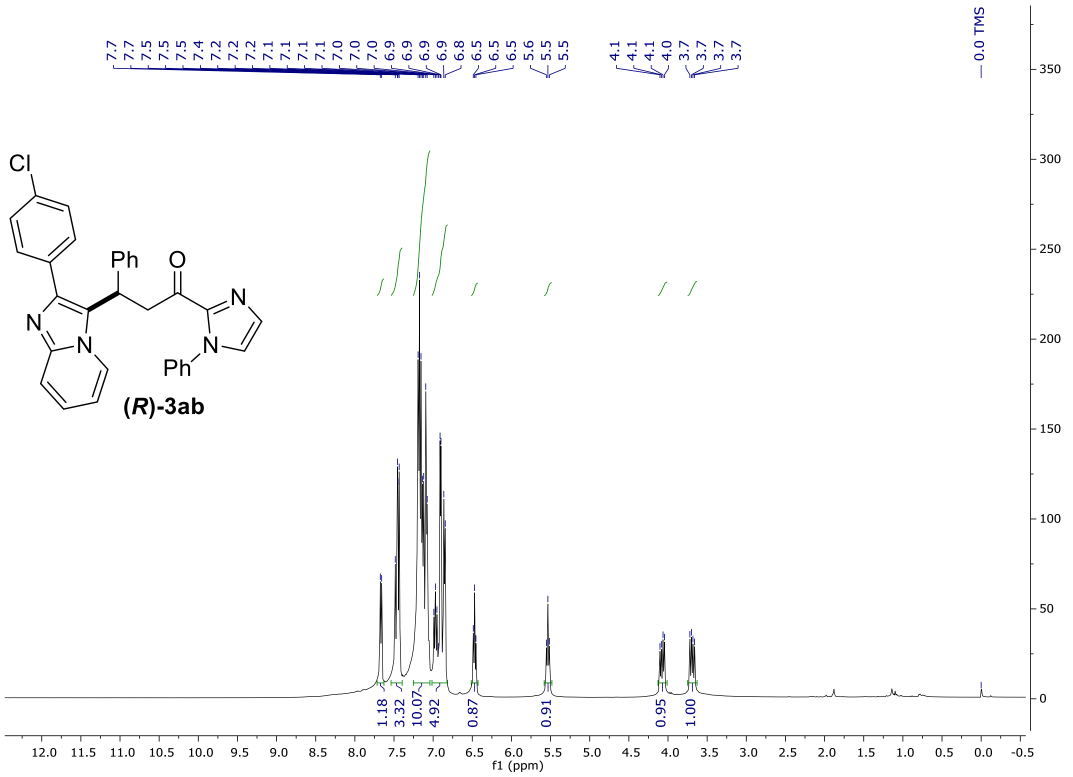

Figure S23. ^{1}H NMR (400 MHz) in $CDCl_3$ of (*R*)-3-(2-(4-chlorophenyl)imidazo[1,2-*α*]pyridin-3-yl)-3-phenyl-1-(1-phenyl-1*H*-imidazol-2-yl)propan-1-one **(*R*)-3ab**.

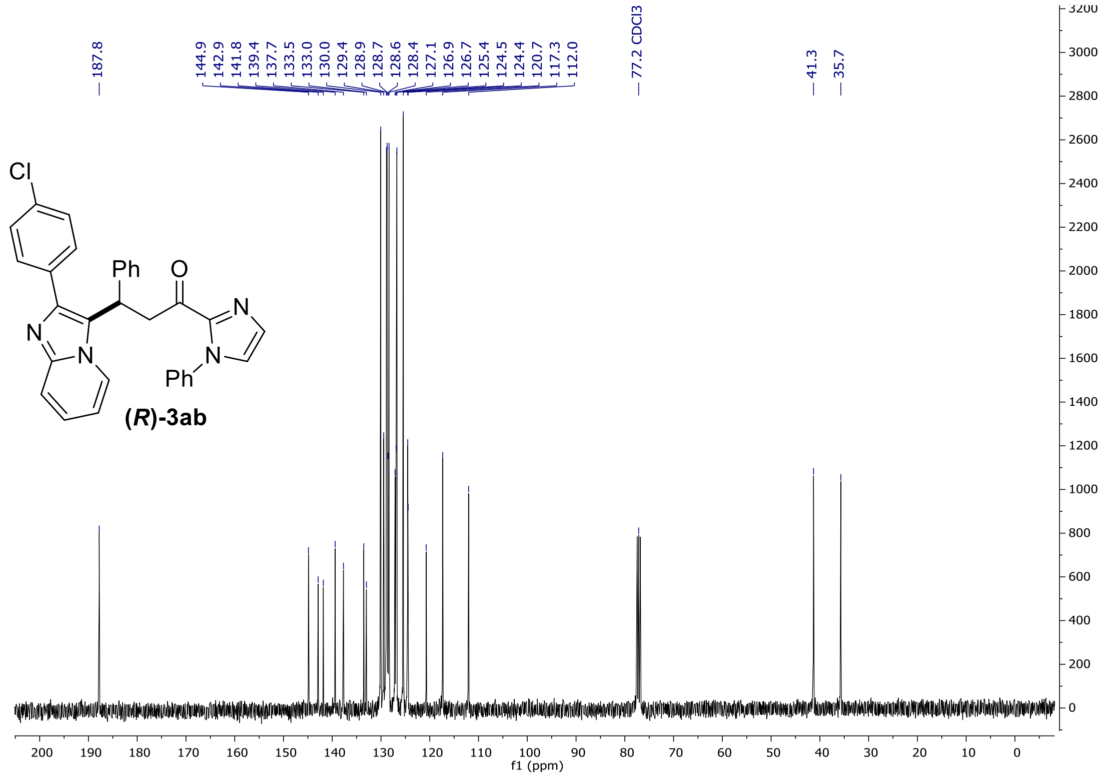

Figure S24. ^{13}C{^{1}H} NMR (100 MHz) in $CDCl_3$ of (*R*)-3-(2-(4-chlorophenyl)imidazo[1,2-*α*]pyridin-3-yl)-3-phenyl-1-(1-phenyl-1*H*-imidazol-2-yl)propan-1-one **(*R*)-3ab**.

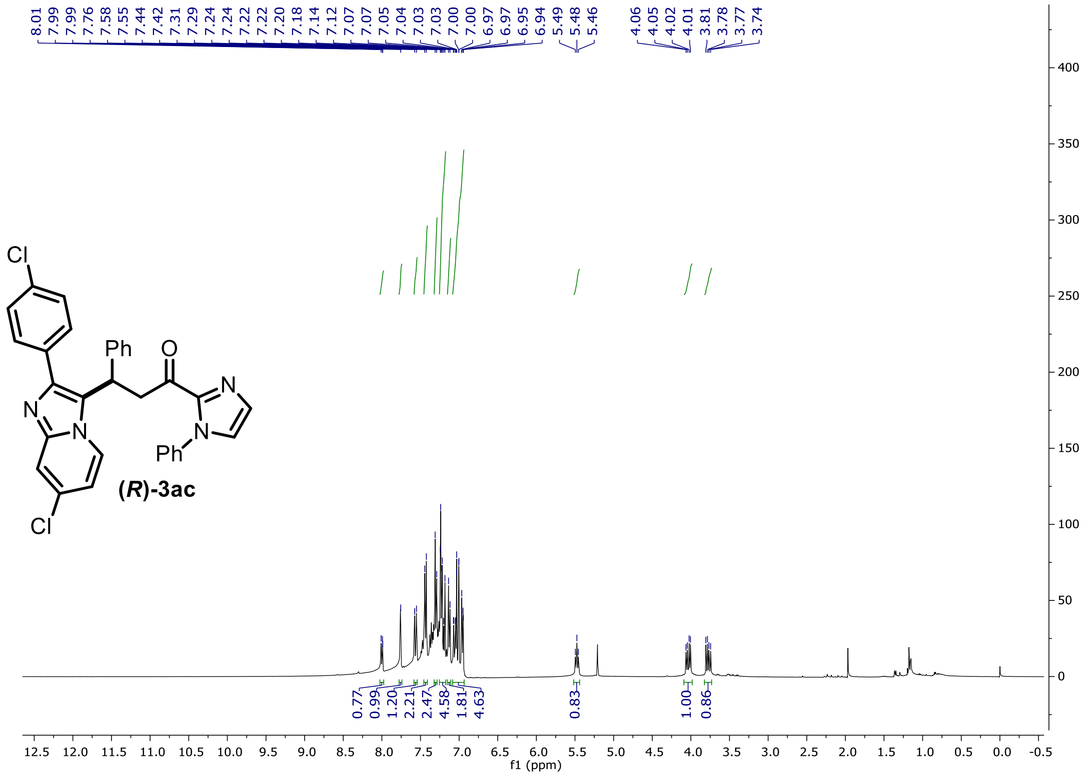

Figure S25. ^{1}H NMR (400 MHz) in $CDCl_3$ of (*R*)-3-(7-chloro-2-(4-chlorophenyl)imidazo[1,2-*α*]pyridin-3-yl)-3-phenyl-1-(1-phenyl-1*H*-imidazol-2-yl)propan-1-one **(*R*)-3ac**.

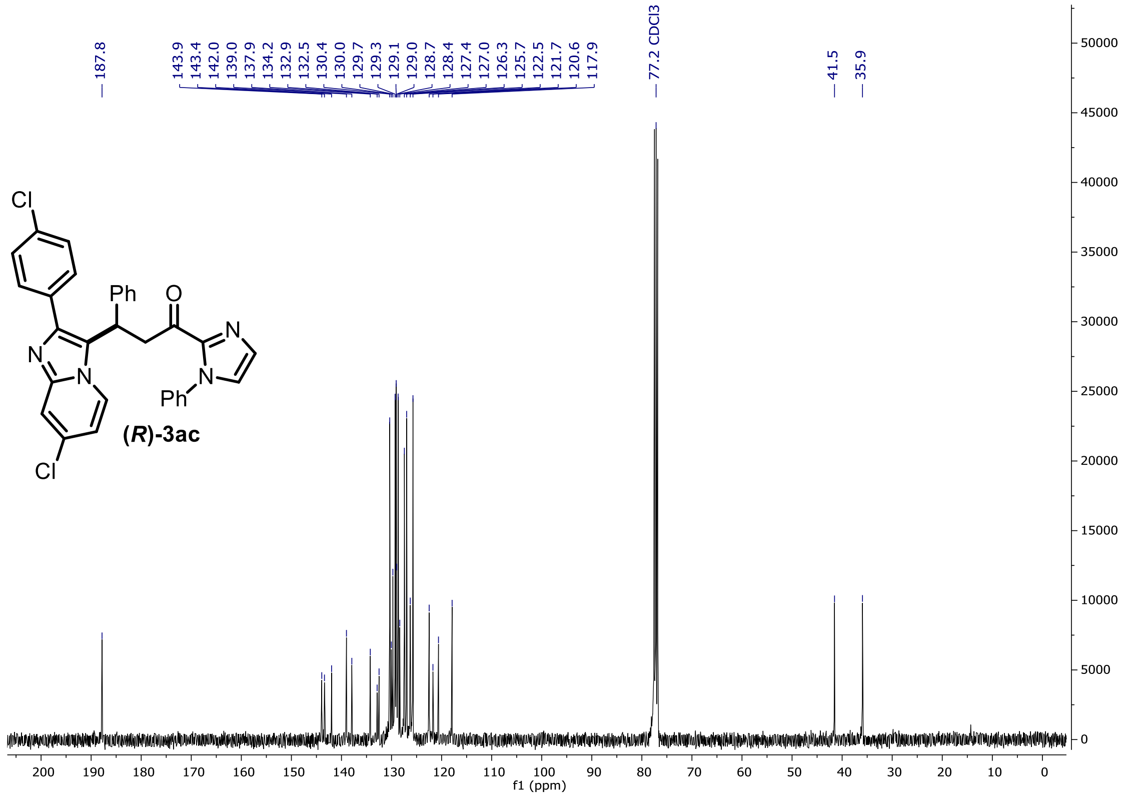

Figure S26. $^{13}C\{^{1}H\}$ NMR (100 MHz) in $CDCl_3$ of (*R*)-3-(7-chloro-2-(4-chlorophenyl)imidazo[1,2-*α*]pyridin-3-yl)-3-phenyl-1-(1-phenyl-1*H*-imidazol-2-yl)propan-1-one **(*R*)-3ac**.

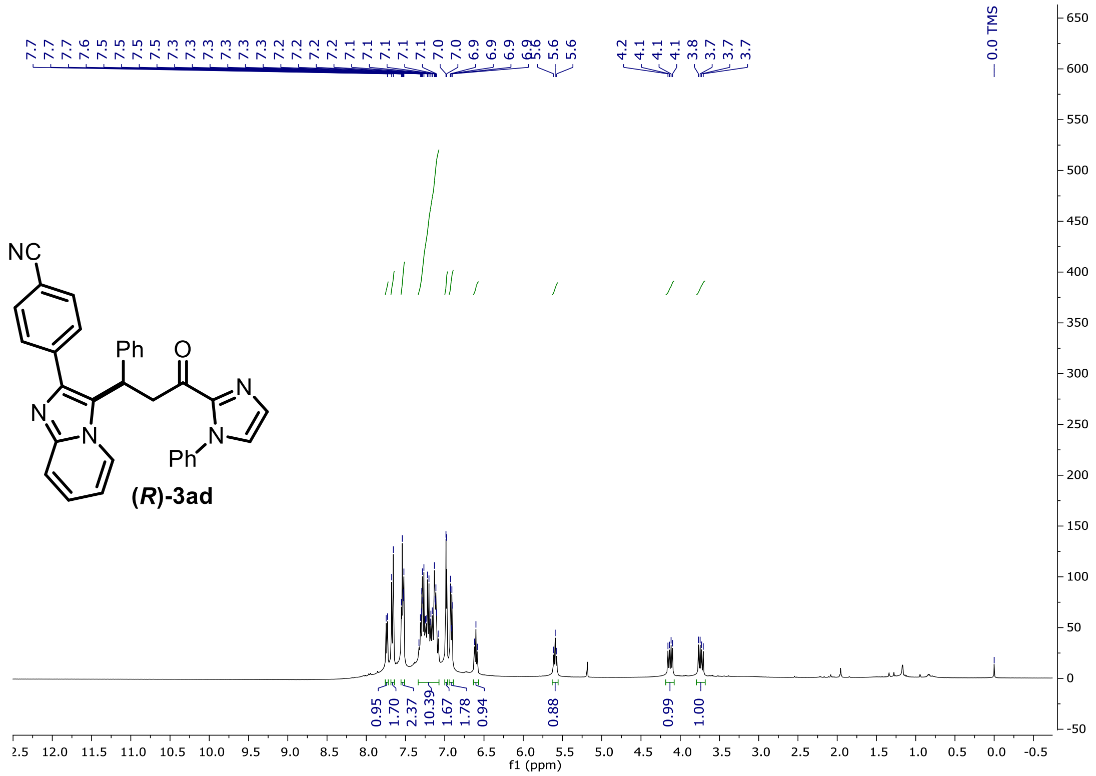

Figure S27. ^{1}H NMR (400 MHz) in $CDCl_3$ of (*R*)-4-(3-(3-oxo-1-phenyl-3-(1-phenyl-1*H*-imidazol-2-yl)propyl)imidazo[1,2-*α*]pyridin-2-yl)benzonitrile **(*R*)-3ad**.

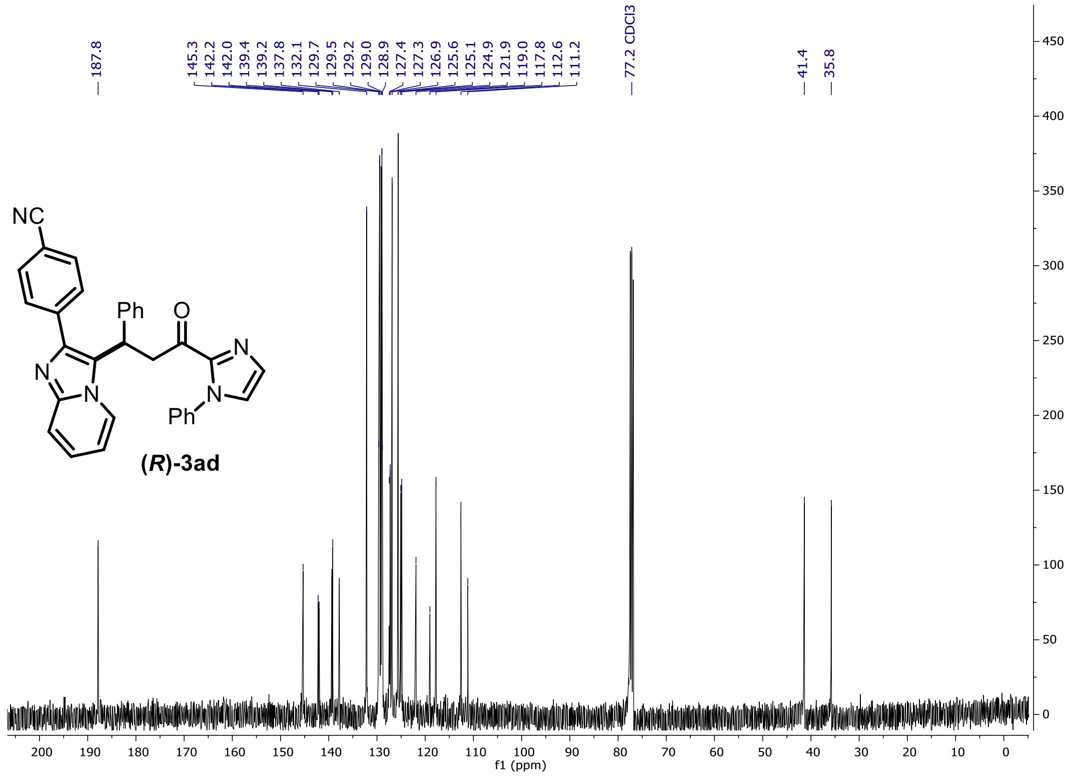

Figure S28. ^{13}C{^{1}H} NMR (100 MHz) in $CDCl_3$ of (*R*)-4-(3-(3-oxo-1-phenyl-3-(1-phenyl-1*H*-imidazol-2-yl)propyl)imidazo[1,2-*α*]pyridin-2-yl)benzonitrile **(*R*)-3ad**.

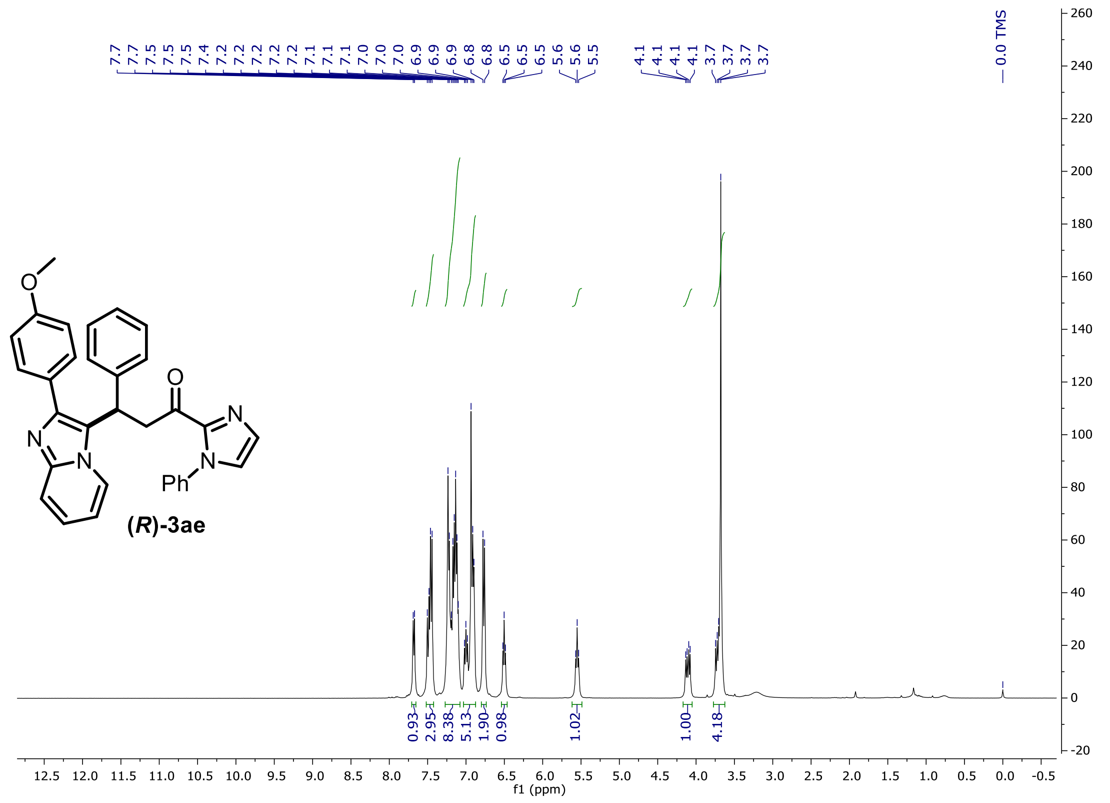

Figure S29. ^{1}H NMR (400 MHz) in $CDCl_3$ of (*R*)-3-(2-(4-methoxyphenyl)imidazo[1,2-*α*]pyridin-3-yl)-3-phenyl-1-(1-phenyl-1*H*-imidazol-2-yl)propan-1-one **(*R*)-3ae**.

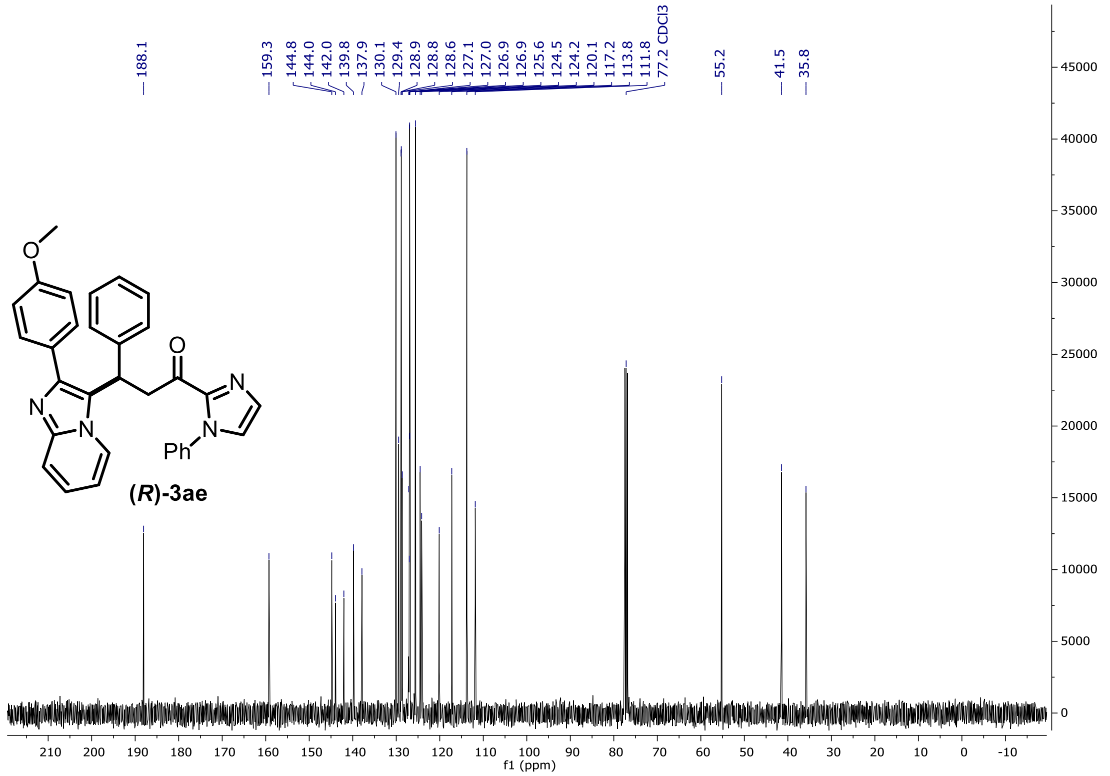

Figure S30. $^{13}C\{^{1}H\}$ NMR (100 MHz) in $CDCl_3$ of (*R*)-3-(2-(4-methoxyphenyl)imidazo[1,2-*α*]pyridin-3-yl)-3-phenyl-1-(1-phenyl-1*H*-imidazol-2-yl)propan-1-one **(*R*)-3ae**.

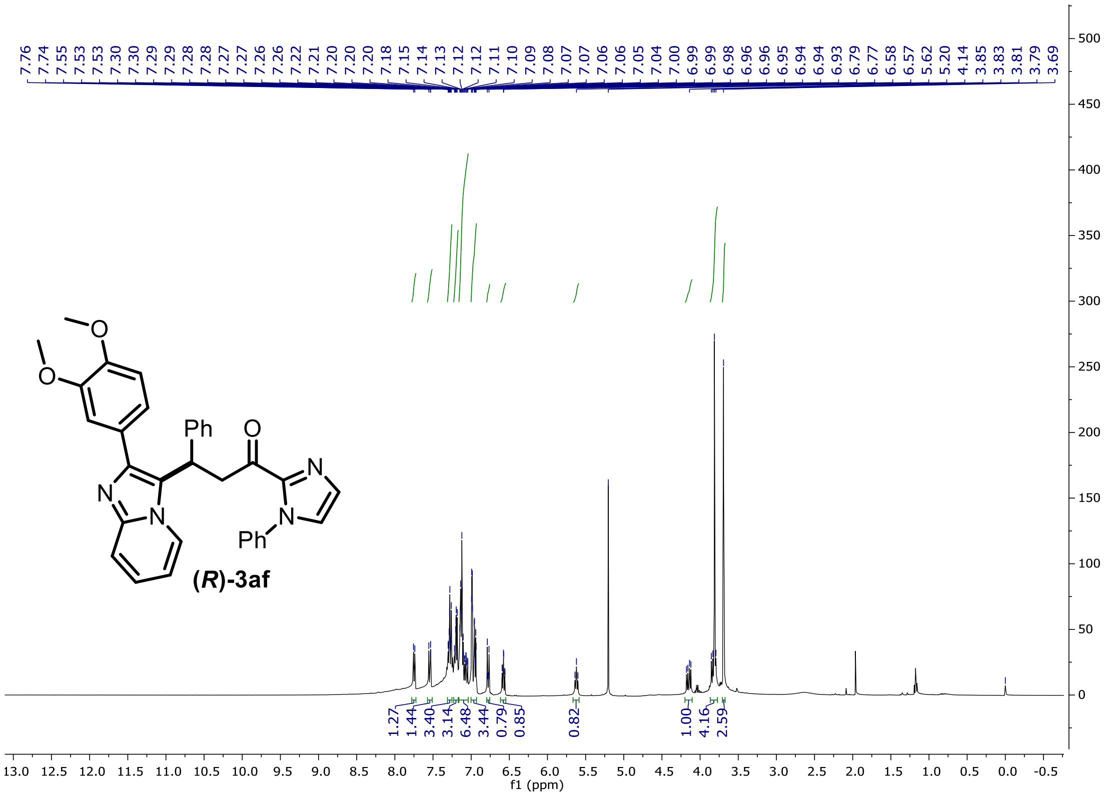

Figure S31. ^{1}H NMR (400 MHz) in $CDCl_3$ of (*R*)-3-(2-(3,4-dimethoxyphenyl)imidazo[1,2-*α*]pyridin-3-yl)-3-phenyl-1-(1-phenyl-1*H*-imidazol-2-yl)propan-1-one **(*R*)-3af**.

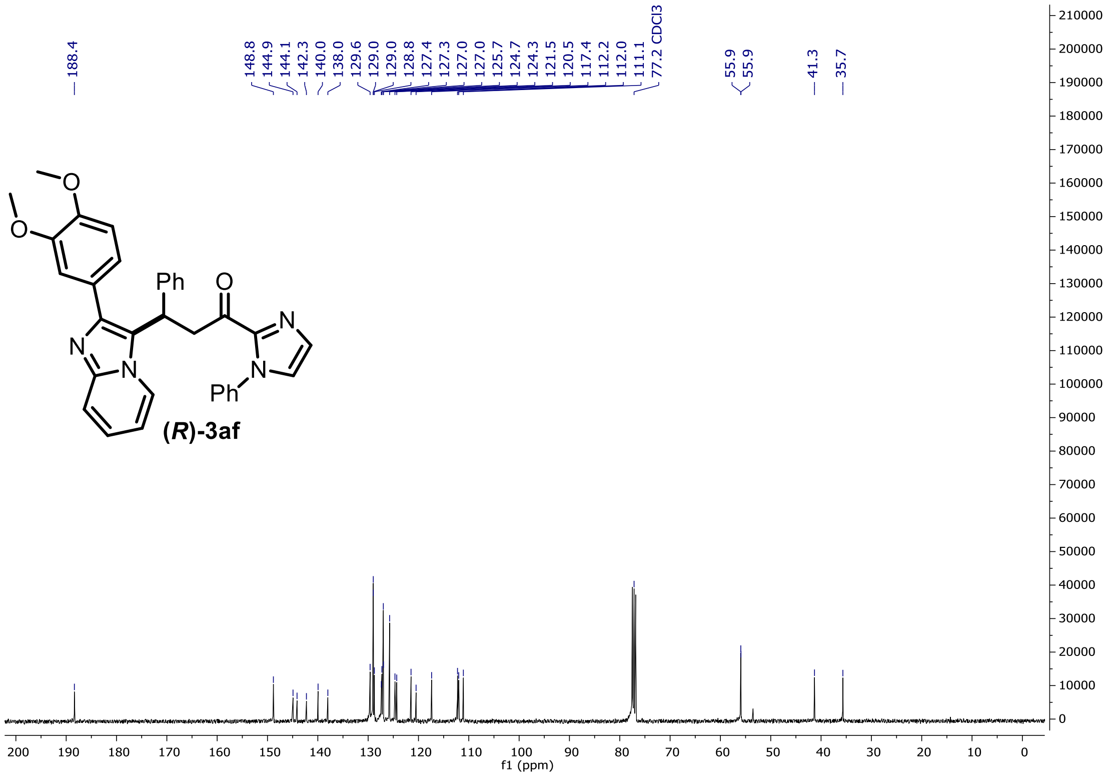

Figure S32. $^{13}C\{^{1}H\}$ NMR (100 MHz) in $CDCl_3$ of (*R*)-3-(2-(3,4-dimethoxyphenyl)imidazo[1,2-*α*]pyridin-3-yl)-3-phenyl-1-(1-phenyl-1*H*-imidazol-2-yl)propan-1-one **(*R*)-3af**.

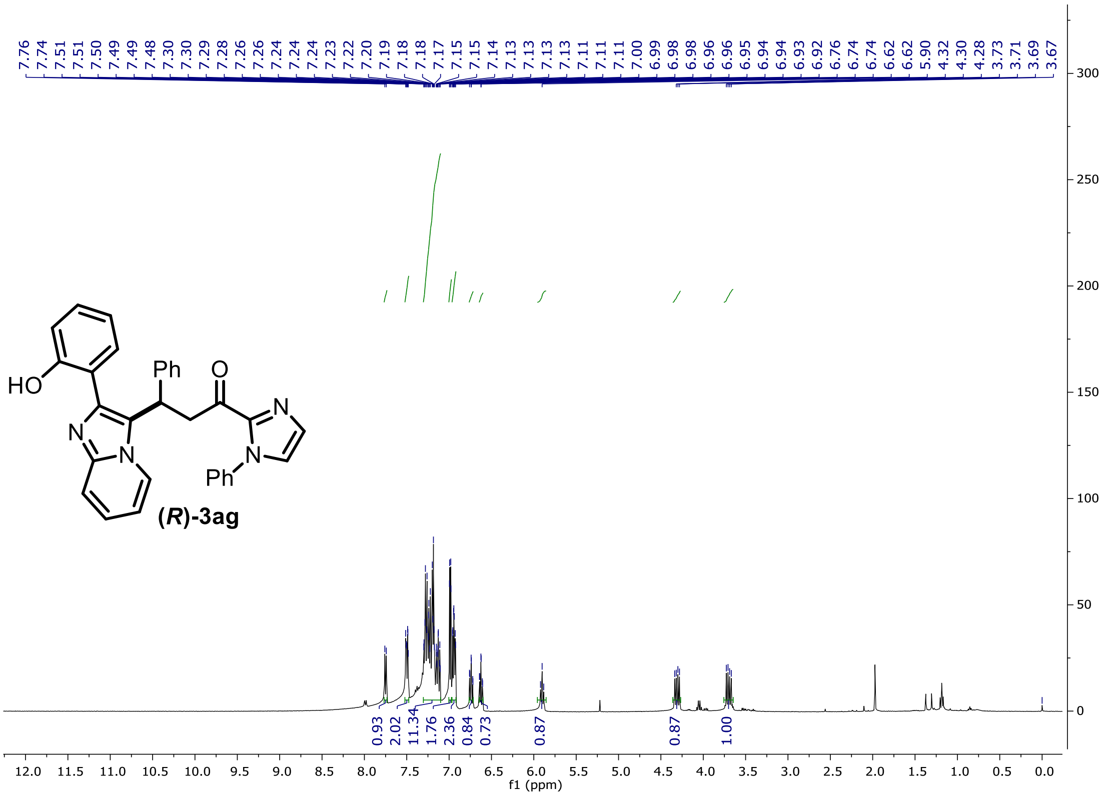

Figure S33. ^{1}H NMR (400 MHz) in $CDCl_3$ of (*R*)-3-(2-(2-hydroxyphenyl)imidazo[1,2-*α*]pyridin-3-yl)-3-phenyl-1-(1-phenyl-1*H*-imidazol-2-yl)propan-1-one **(*R*)-3ag**.

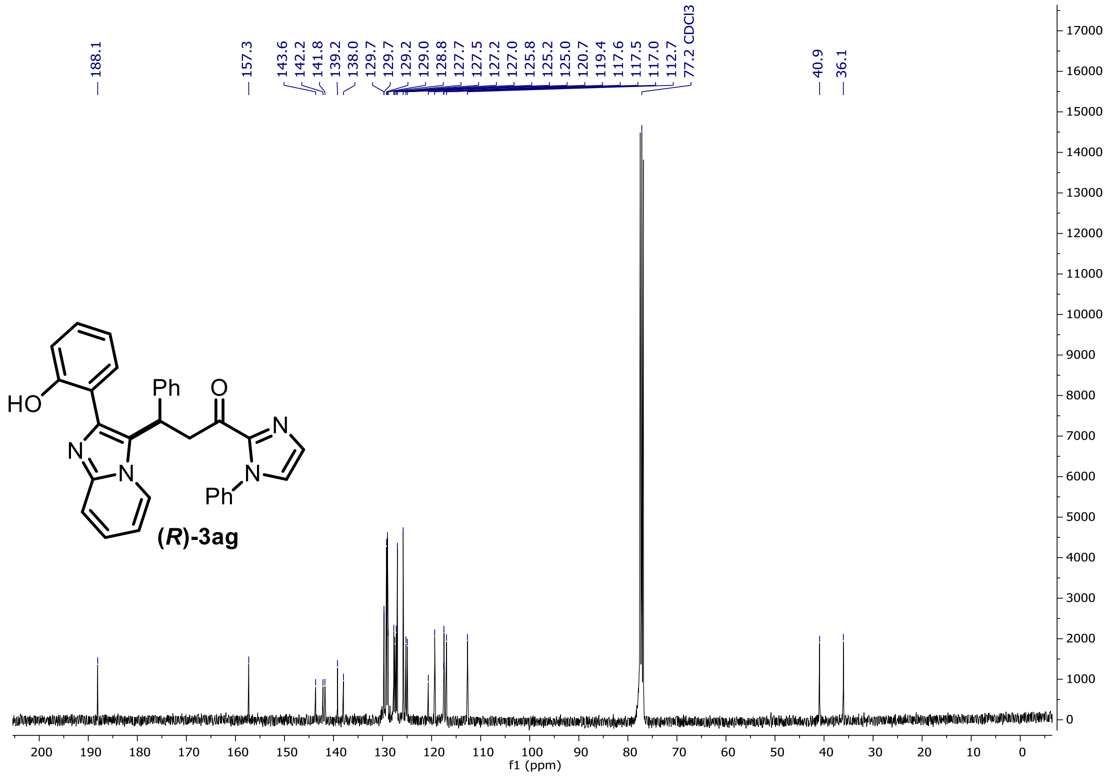

Figure S34. $^{13}C\{^{1}H\}$ NMR (100 MHz) in $CDCl_3$ of (*R*)-3-(2-(2-hydroxyphenyl)imidazo[1,2-*α*]pyridin-3-yl)-3-phenyl-1-(1-phenyl-1*H*-imidazol-2-yl)propan-1-one **(*R*)-3ag**.

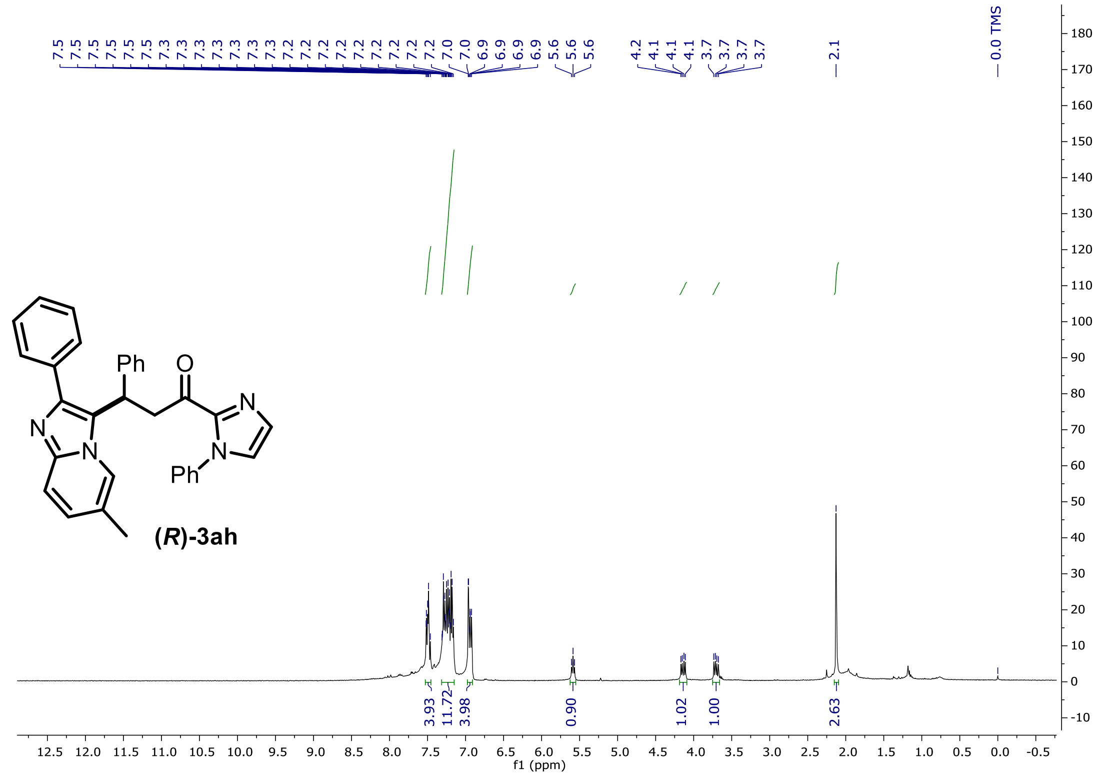

Figure S35. ^{1}H NMR (400 MHz) in $CDCl_3$ of (*R*)-3-(6-methyl-2-phenylimidazo[1,2-*α*]pyridin-3-yl)-3-phenyl-1-(1-phenyl-1*H*-imidazol-2-yl)propan-1-one **(*R*)-3ah**.

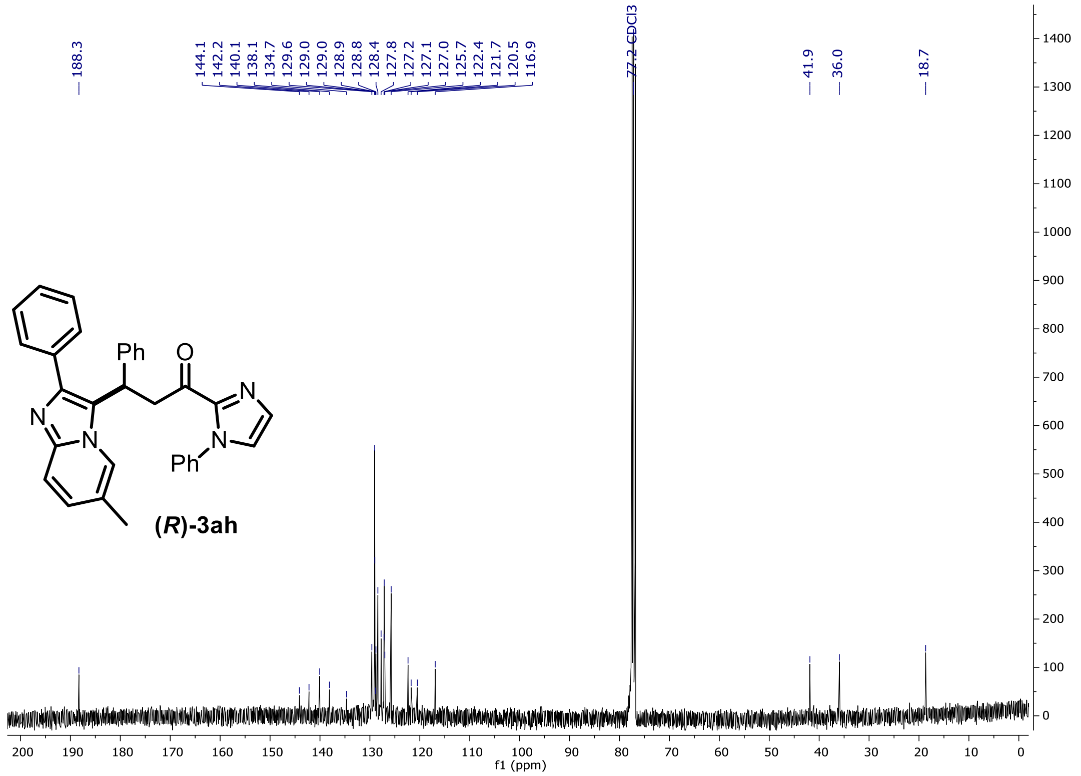

Figure S36. $^{13}C\{^{1}H\}$ NMR (100 MHz) in $CDCl_3$ of (*R*)-3-(6-methyl-2-phenylimidazo[1,2-*α*]pyridin-3-yl)-3-phenyl-1-(1-phenyl-1*H*-imidazol-2-yl)propan-1-one **(*R*)-3ah**.

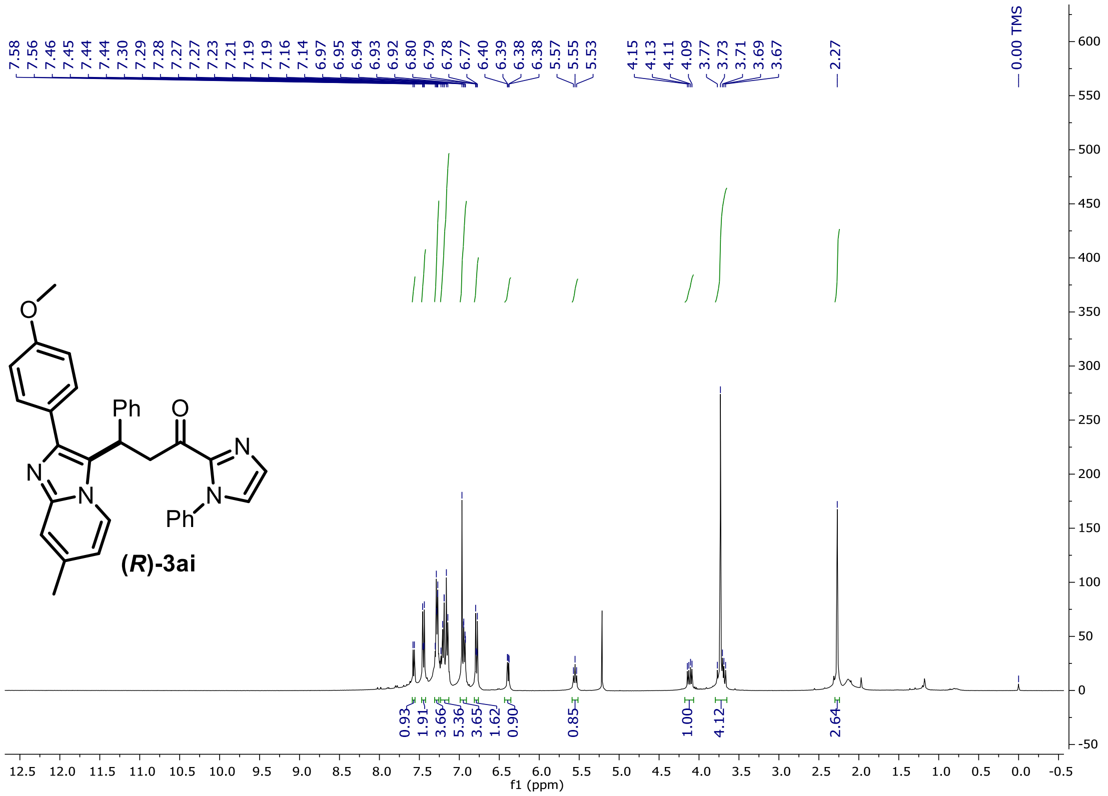

Figure S37. ^{1}H NMR (400 MHz) in $CDCl_3$ of (*R*)-3-(2-(4-methoxyphenyl)-7-methylimidazo[1,2-*α*]pyridin-3-yl)-3-phenyl-1-(1-phenyl-1*H*-imidazol-2-yl)propan-1-one **(*R*)-3ai**.

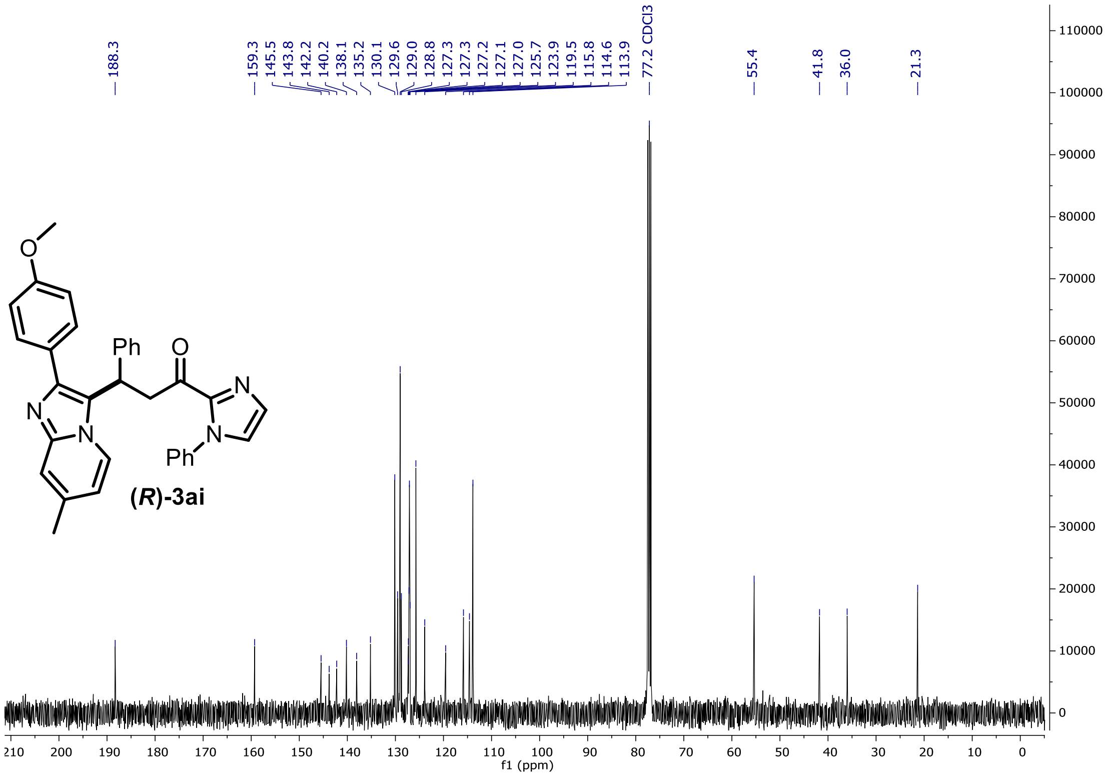

Figure S38. $^{13}C\{^{1}H\}$ NMR (100 MHz) in $CDCl_3$ of (*R*)-3-(2-(4-methoxyphenyl)-7-methylimidazo[1,2-*α*]pyridin-3-yl)-3-phenyl-1-(1-phenyl-1*H*-imidazol-2-yl)propan-1-one **(*R*)-3ai**.

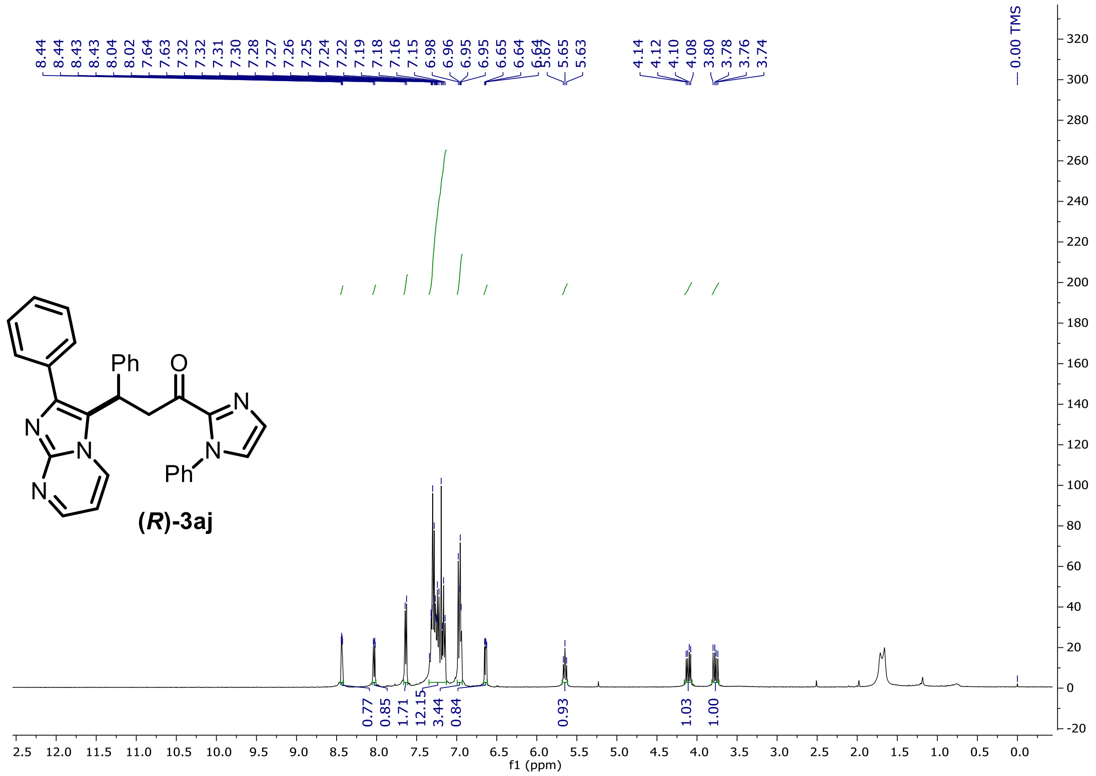

Figure S39. ^{1}H NMR (400 MHz) in $CDCl_3$ of (*R*)-3-phenyl-1-(1-phenyl-1*H*-imidazol-2-yl)-3-(2-phenylimidazo[1,2-*α*]pyrimidin-3-yl)propan-1-one **(*R*)-3aj**.

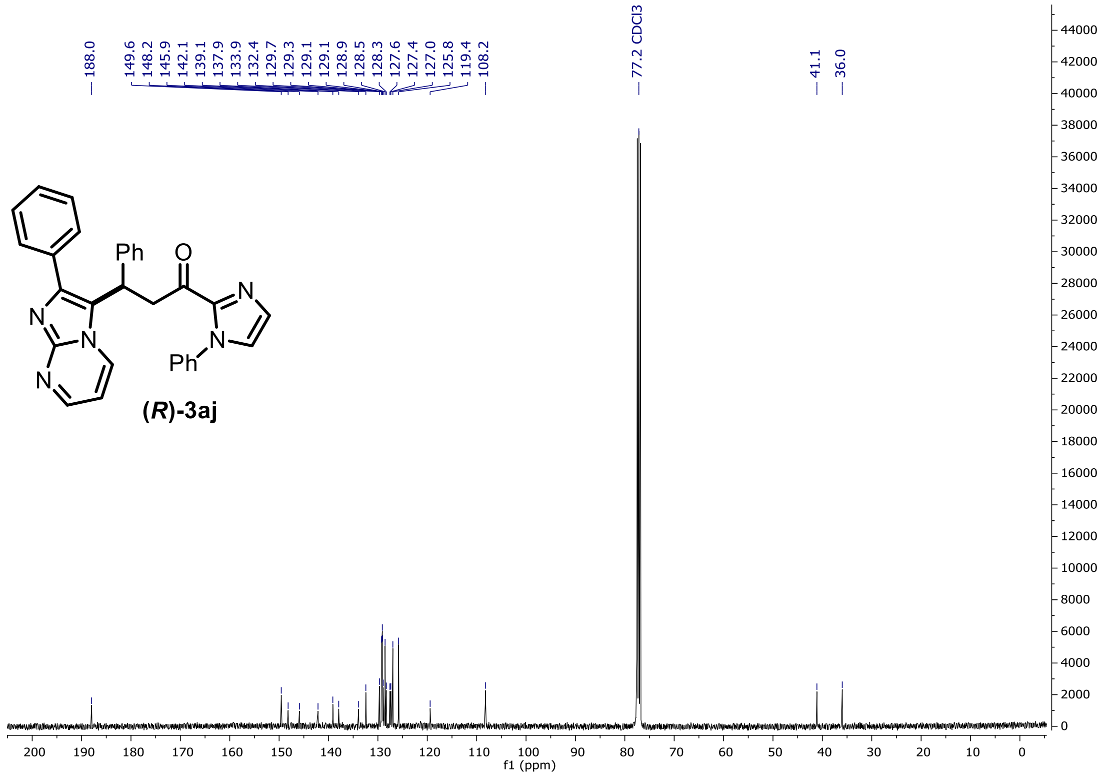

Figure S40. ^{13}C{^{1}H} NMR (100 MHz) in $CDCl_3$ of (*R*)-3-phenyl-1-(1-phenyl-1*H*-imidazol-2-yl)-3-(2-phenylimidazo[1,2-*α*]pyrimidin-3-yl)propan-1-one **(*R*)-3aj**.

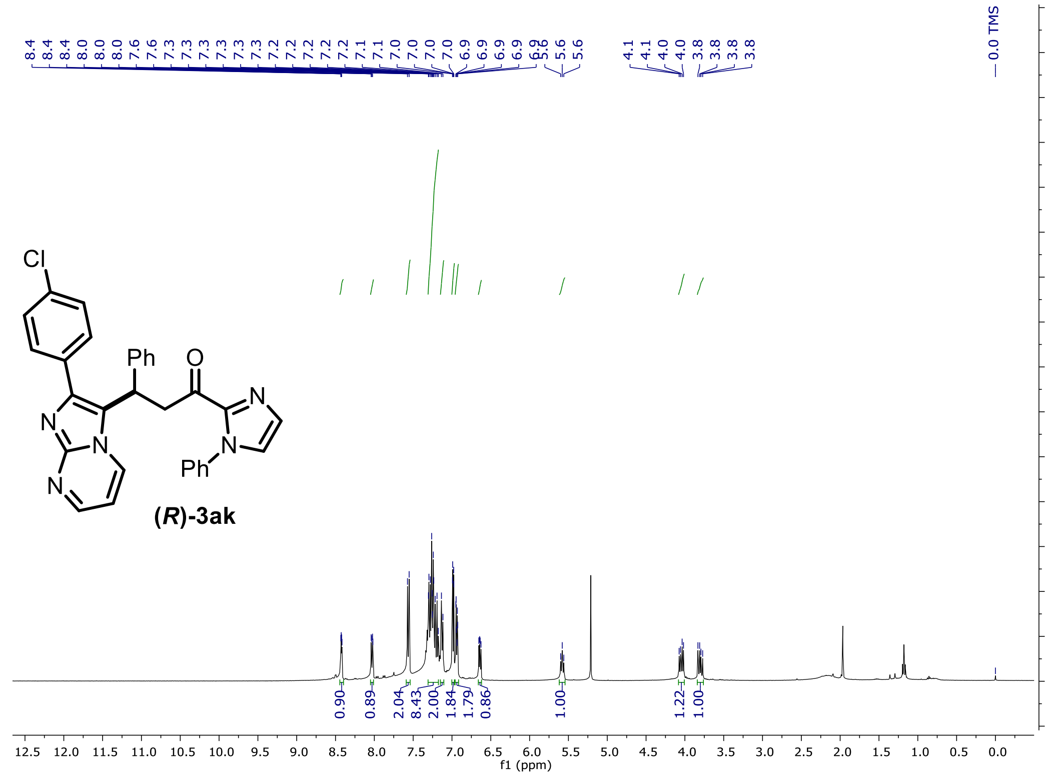

Figure S41. ^{1}H NMR (400 MHz) in $CDCl_3$ of (*R*)-3-(2-(4-chlorophenyl)imidazo[1,2-*α*]pyrimidin-3-yl)-3-phenyl-1-(1-phenyl-1*H*-imidazol-2-yl)propan-1-one **(*R*)-3ak**.

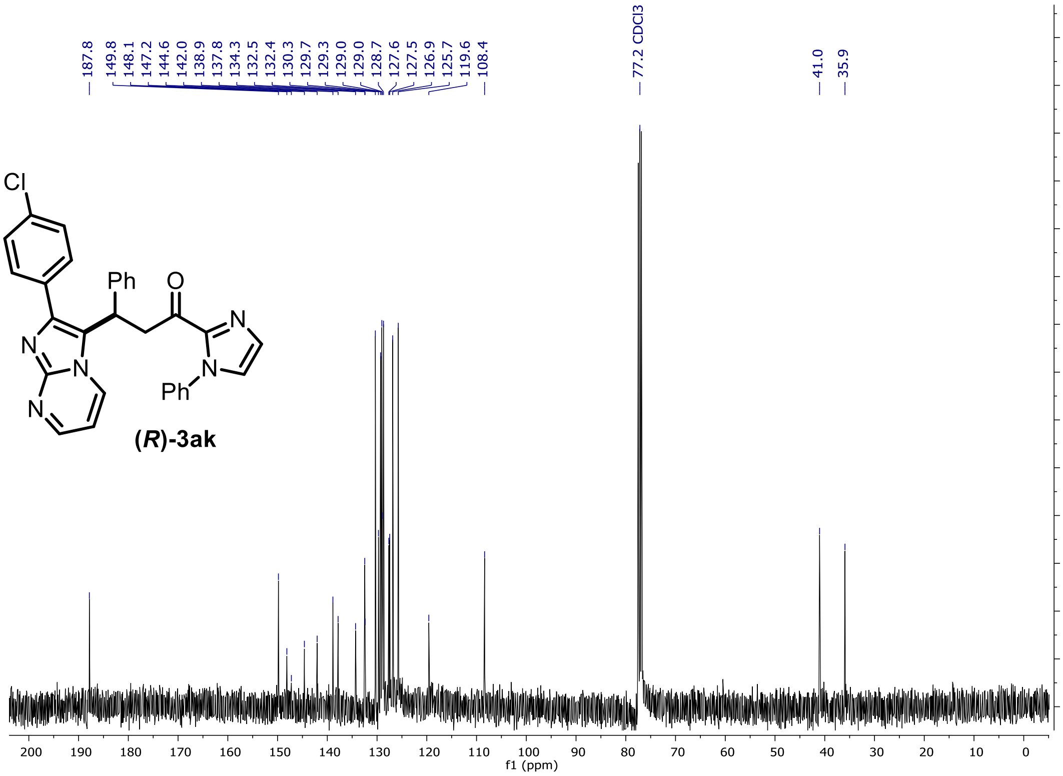

Figure S42. ^{13}C{^{1}H} NMR (100 MHz) in $CDCl_3$ of (*R*)-3-(2-(4-chlorophenyl)imidazo[1,2-*α*]pyrimidin-3-yl)-3-phenyl-1-(1-phenyl-1*H*-imidazol-2-yl)propan-1-one **(*R*)-3ak**.

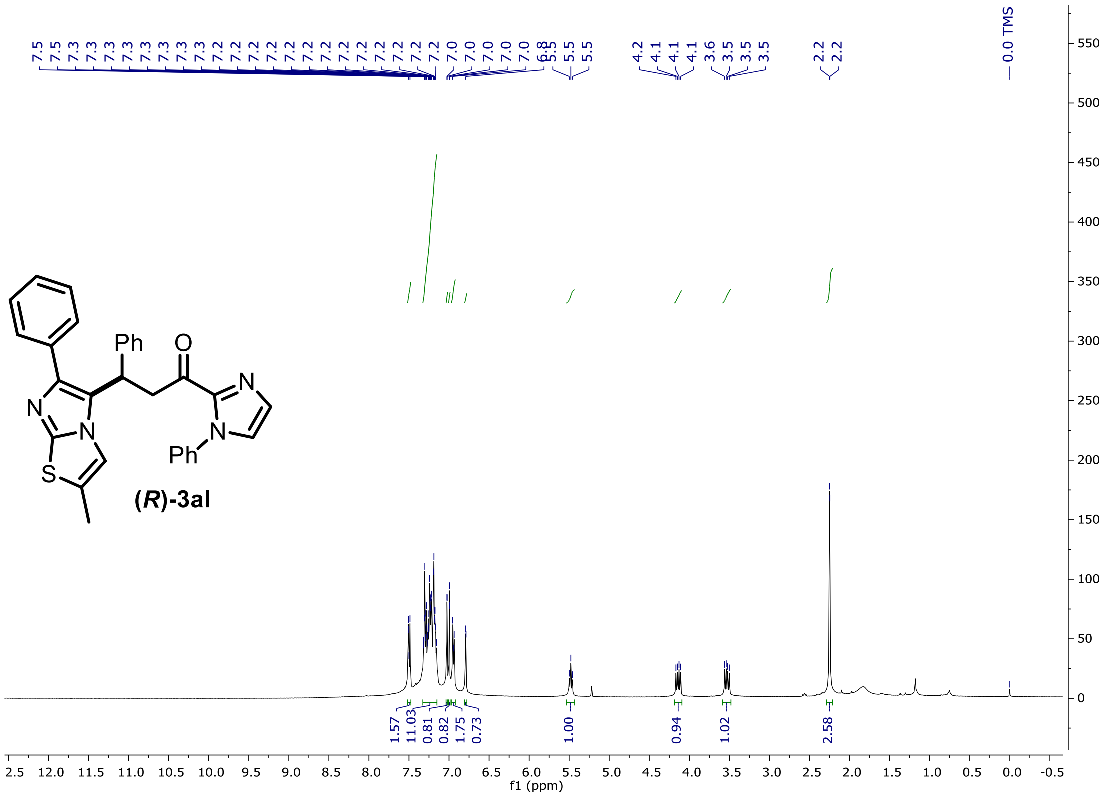

Figure S43. ^{1}H NMR (400 MHz) in $CDCl_3$ of (*R*)-3-(2-methyl-6-phenylimidazo[2,1-*b*]thiazol-5-yl)-3-phenyl-1-(1-phenyl-1*H*-imidazol-2-yl)propan-1-one **(*R*)-3al**.

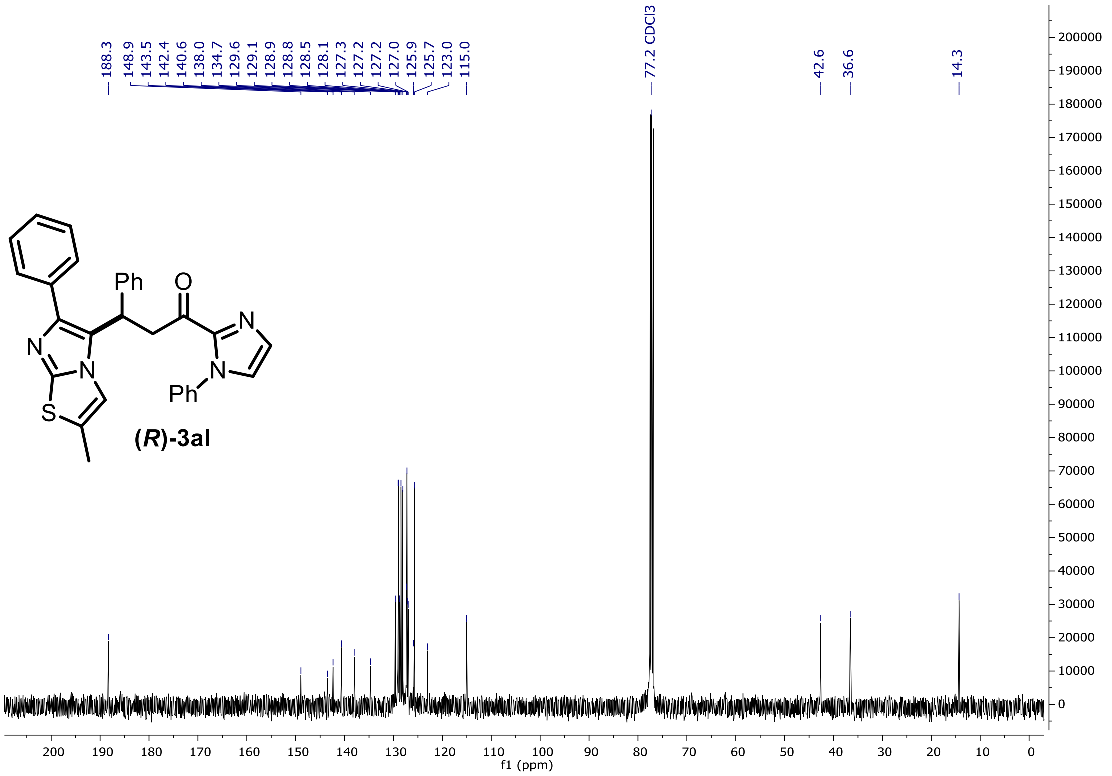

Figure S44. $^{13}C\{^{1}H\}$ NMR (100 MHz) in $CDCl_3$ of (*R*)-3-(2-methyl-6-phenylimidazo[2,1-*b*]thiazol-5-yl)-3-phenyl-1-(1-phenyl-1*H*-imidazol-2-yl)propan-1-one **(*R*)-3al**.

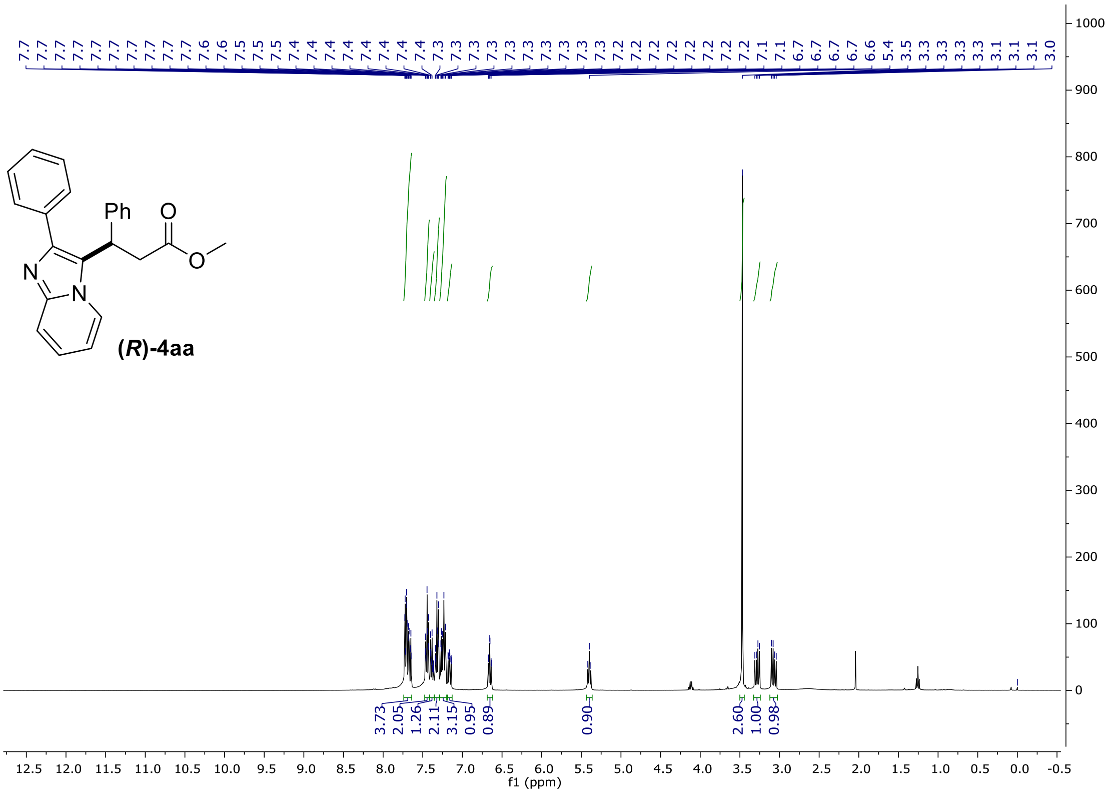

Figure S45. ^{1}H NMR (400 MHz) in $CDCl_3$ of methyl-(*R*)-3-phenyl-3-(2-phenylimidazo[1,2-*a*]pyridin-3-yl)propanoate **(*R*)-4aa**.

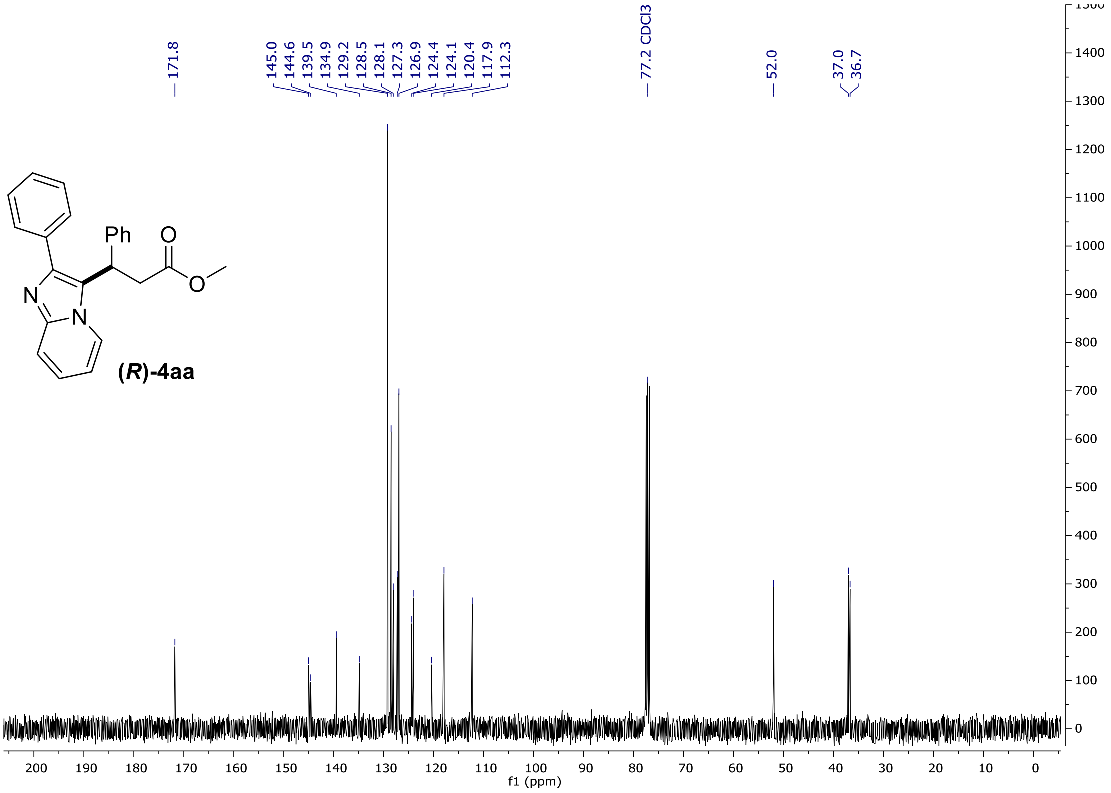

Figure S46. $^{13}C\{^{1}H\}$ NMR (100 MHz) in $CDCl_3$ of methyl-(*R*)-3-phenyl-3-(2-phenylimidazo[1,2-*a*]pyridin-3-yl)propanoate **(*R*)-4aa**.

2. Chiral HPLC analysis

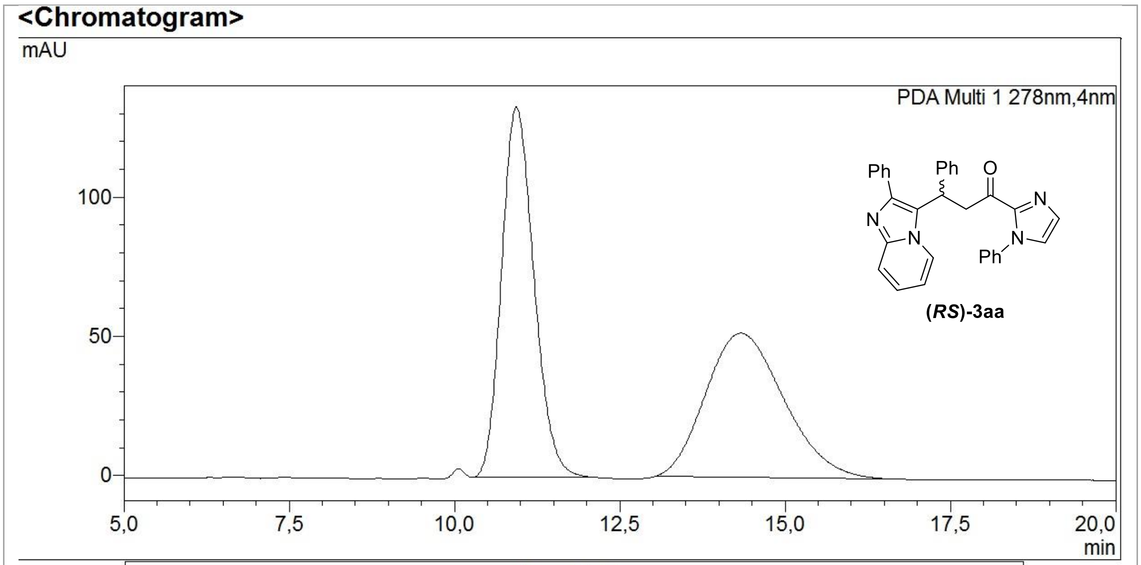

Peak Table

PDA Ch1 278nm

Peak#	Resolution(USP)	Ret. Time	Area	Area%
1	--	10,937	4531834	51,640
2	2,193	14,329	4243990	48,360
Total			8775823	100,000

Figure S47. Racemic chromatogram of the compound **(*RS*)-3aa**.

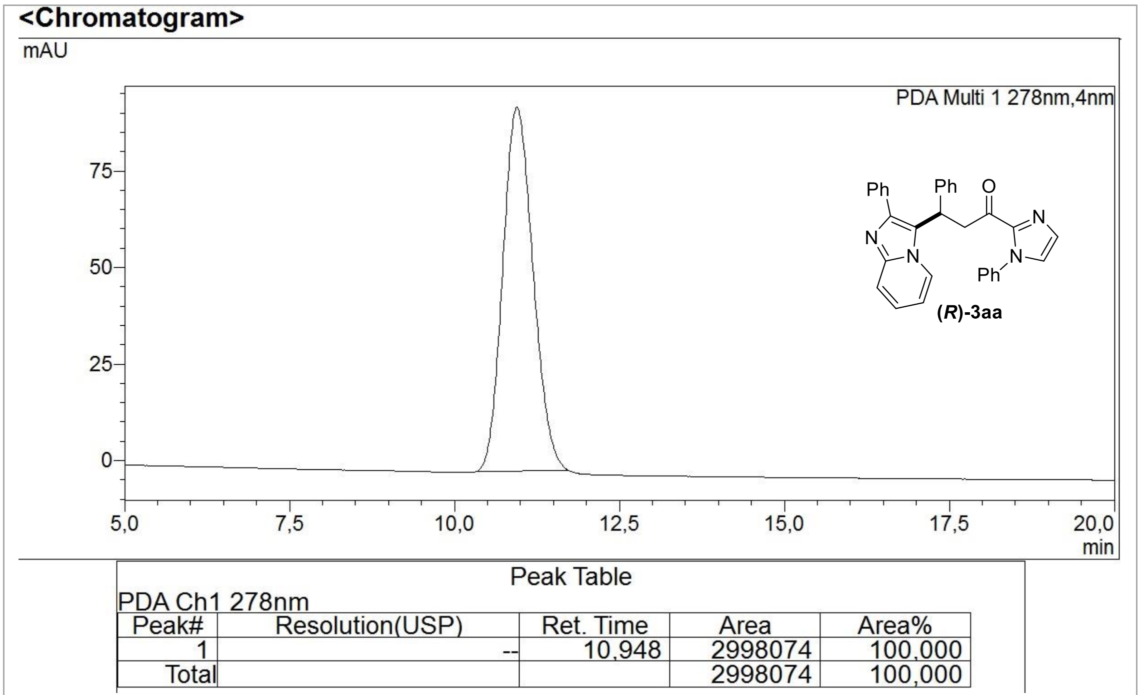

Peak Table

PDA Ch1 278nm

Peak#	Resolution(USP)	Ret. Time	Area	Area%
1	--	10,948	2998074	100,000
Total			2998074	100,000

Figure S48. Chiral chromatogram of the compound **(*R*)-3aa**.

<Chromatogram>

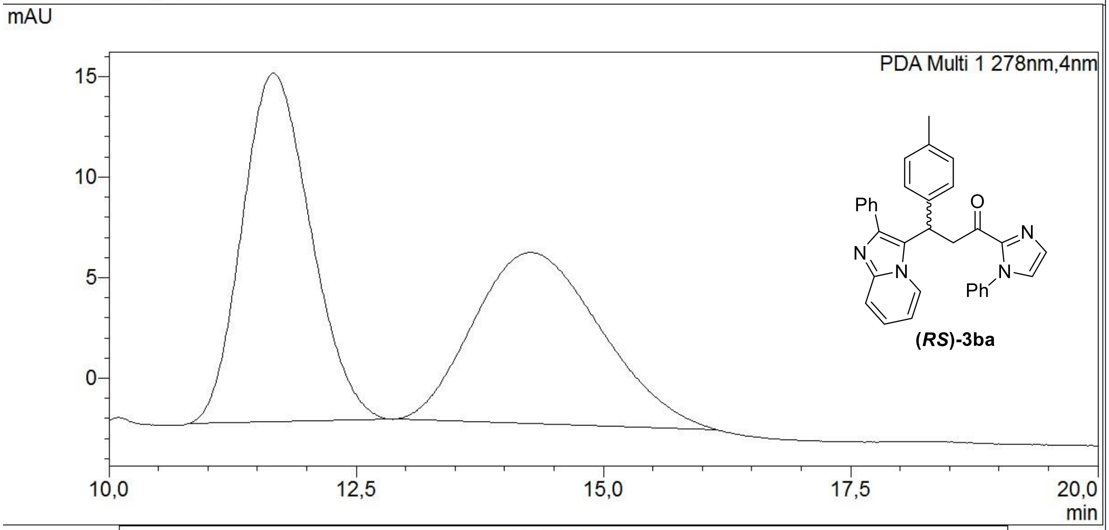

Peak Table

PDA Ch1 278nm

Peak#	Resolution(USP)	Ret. Time	Area	Area%
1	--	11,664	835581	52,270
2	1,404	14,277	763013	47,730
Total			1598594	100,000

Figure S49. Racemic chromatogram of the compound **(*RS*)-3ba**.

<Chromatogram>

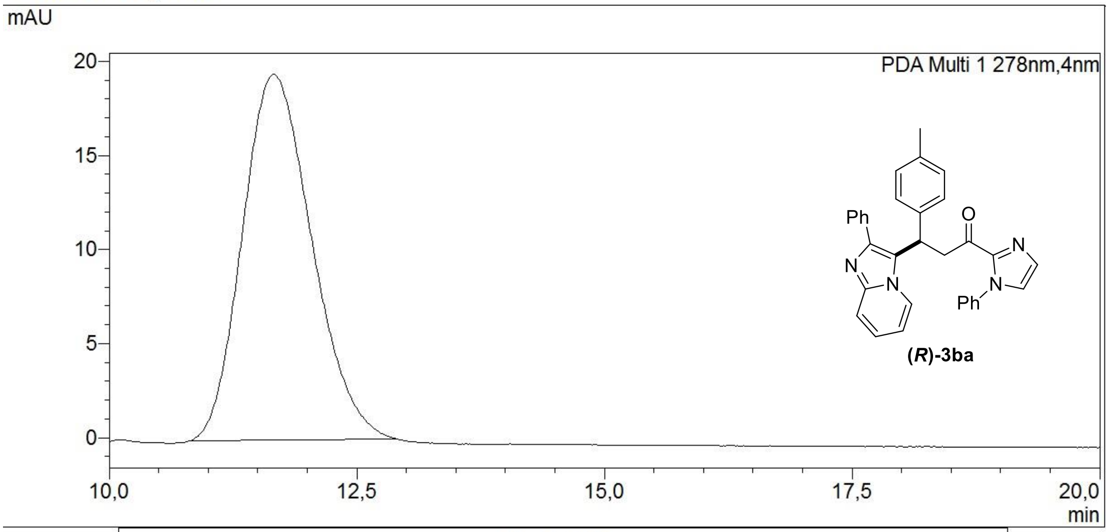

Peak Table

PDA Ch1 278nm

Peak#	Resolution(USP)	Ret. Time	Area	Area%
1	--	11,662	949075	100,000
Total			949075	100,000

Figure S50. Chiral chromatogram of the compound **(*R*)-3ba**.

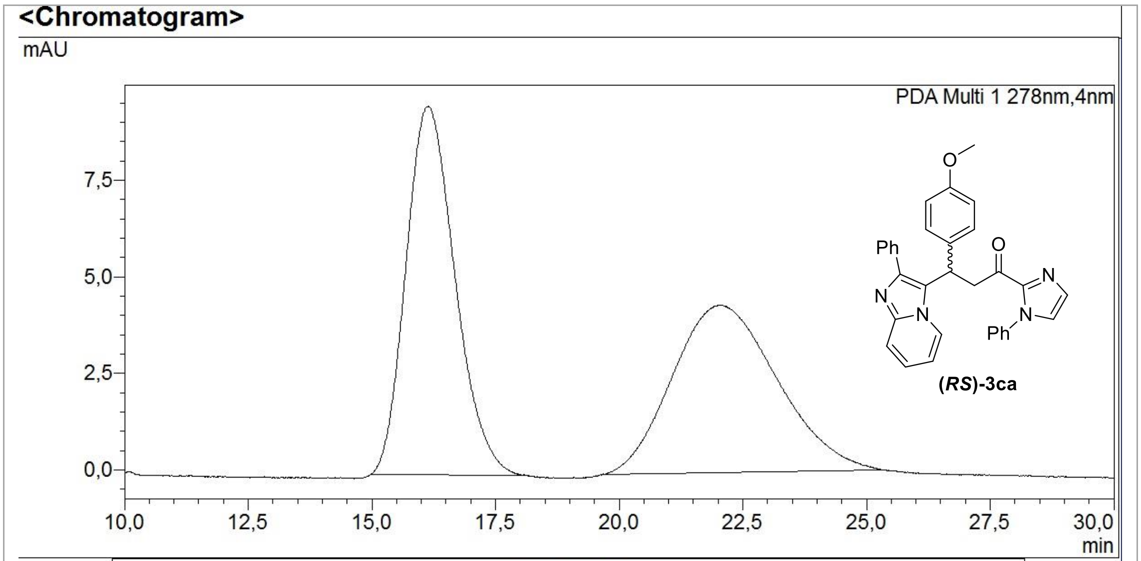

Peak Table

PDA Ch1 278nm

Peak#	Resolution(USP)	Ret. Time	Area	Area%
1	--	16,137	658179	50,863
2	2,077	22,070	635856	49,137
Total			1294035	100,000

Figure S51. Racemic chromatogram of the compound **(*RS*)-3ca**.

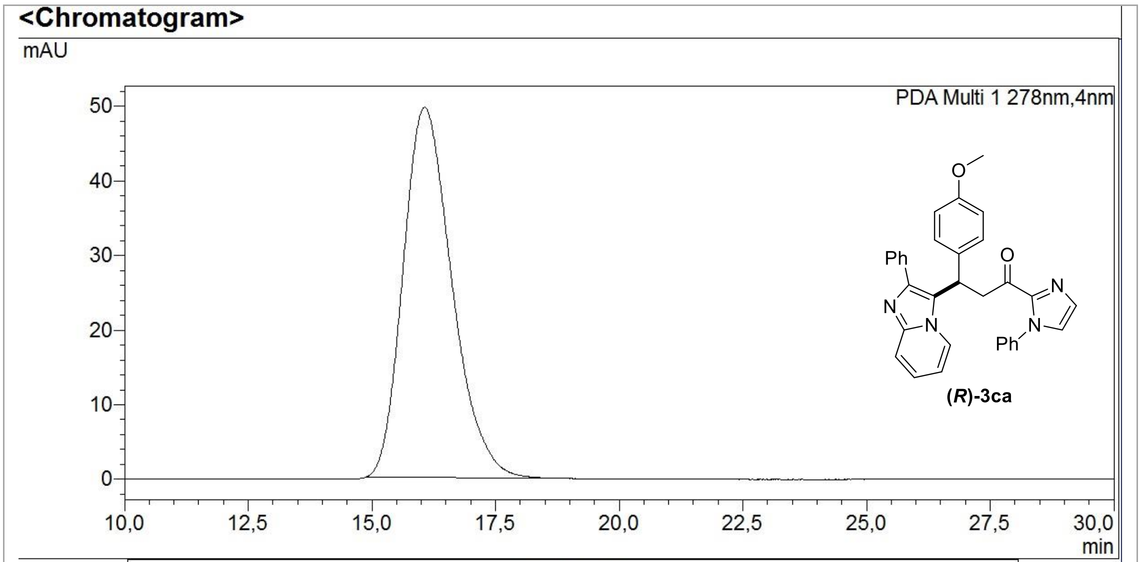

Peak Table

PDA Ch1 278nm

Peak#	Resolution(USP)	Ret. Time	Area	Area%
1	--	16,068	3406378	100,000
Total			3406378	100,000

Figure S52. Chiral chromatogram of the compound **(*R*)-3ca**.

<Chromatogram>

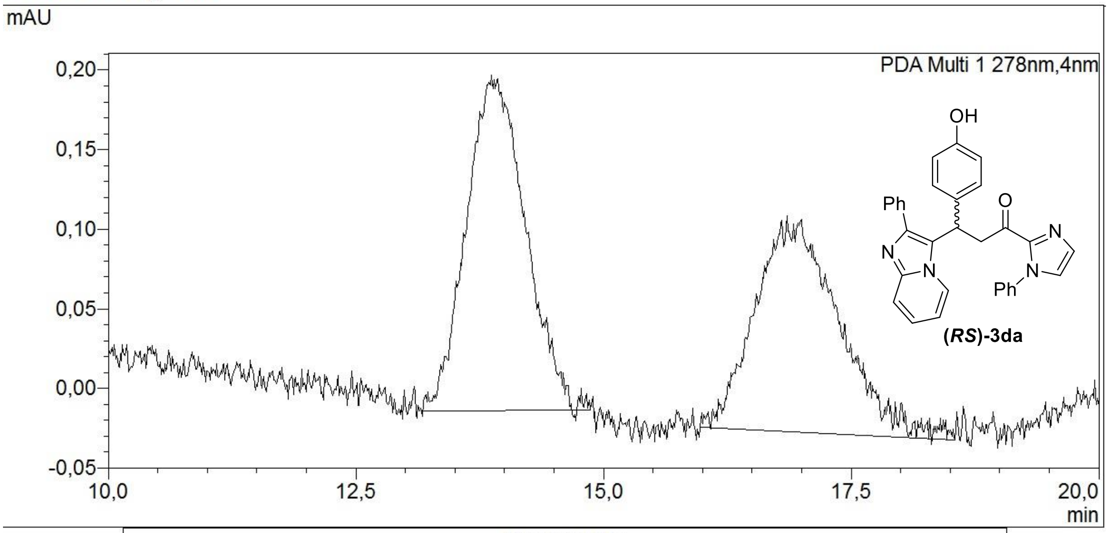

Peak Table

PDA Ch1 278nm

Peak#	Resolution(USP)	Ret. Time	Area	Area%
1	--	13,867	8726	52,467
2	2,211	16,852	7906	47,533
Total			16632	100,000

Figure S53. Racemic chromatogram of the compound **(*RS*)-3da**.

<Chromatogram>

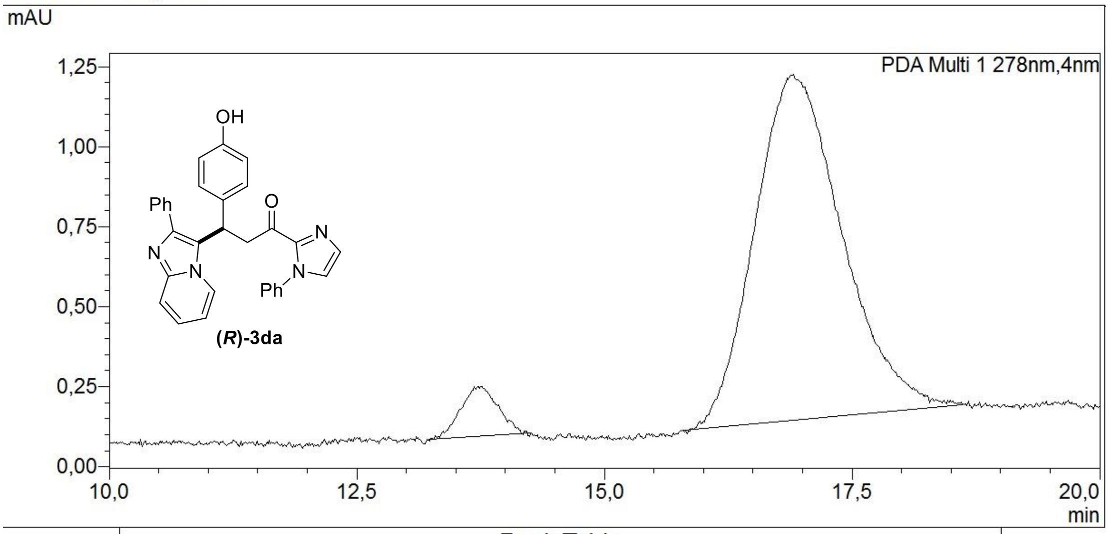

Peak Table

PDA Ch1 278nm

Peak#	Resolution(USP)	Ret. Time	Area	Area%
1	--	13,767	4116	5,841
2	2,687	16,905	66352	94,159
Total			70468	100,000

Figure S54. Chiral chromatogram of the compound **(*R*)-3da**.

<Chromatogram>

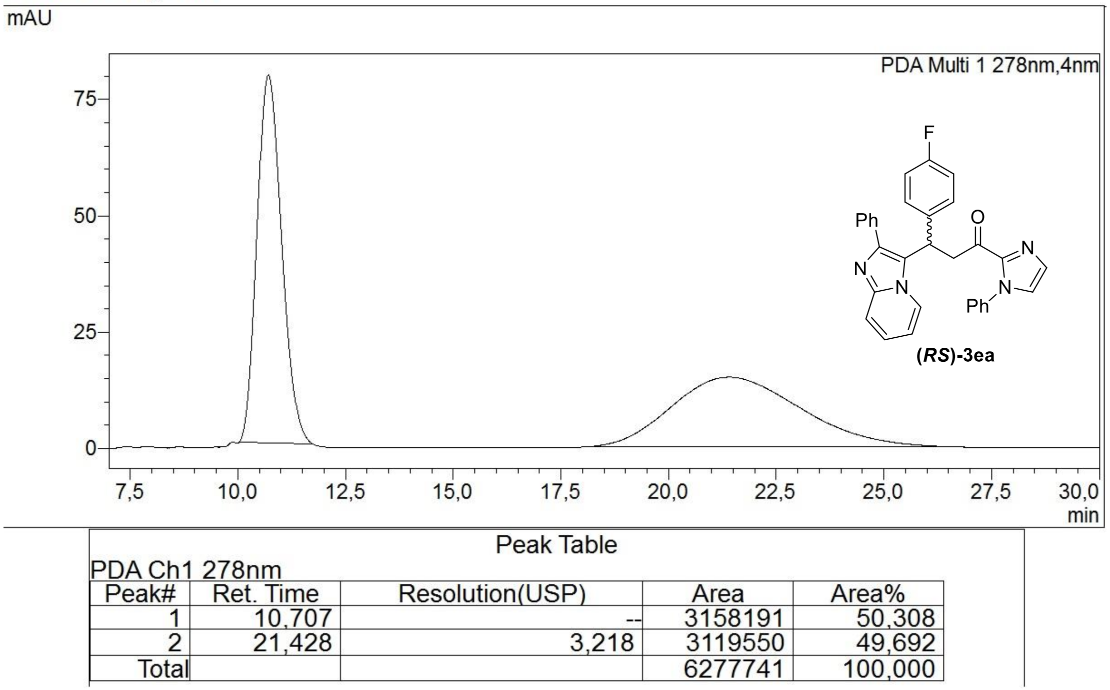

Peak Table

PDA Ch1 278nm

Peak#	Ret. Time	Resolution(USP)	Area	Area%
1	10,707	--	3158191	50,308
2	21,428	3,218	3119550	49,692
Total			6277741	100,000

Figure S55. Racemic chromatogram of the compound **(*RS*)-3ea**.

<Chromatogram>

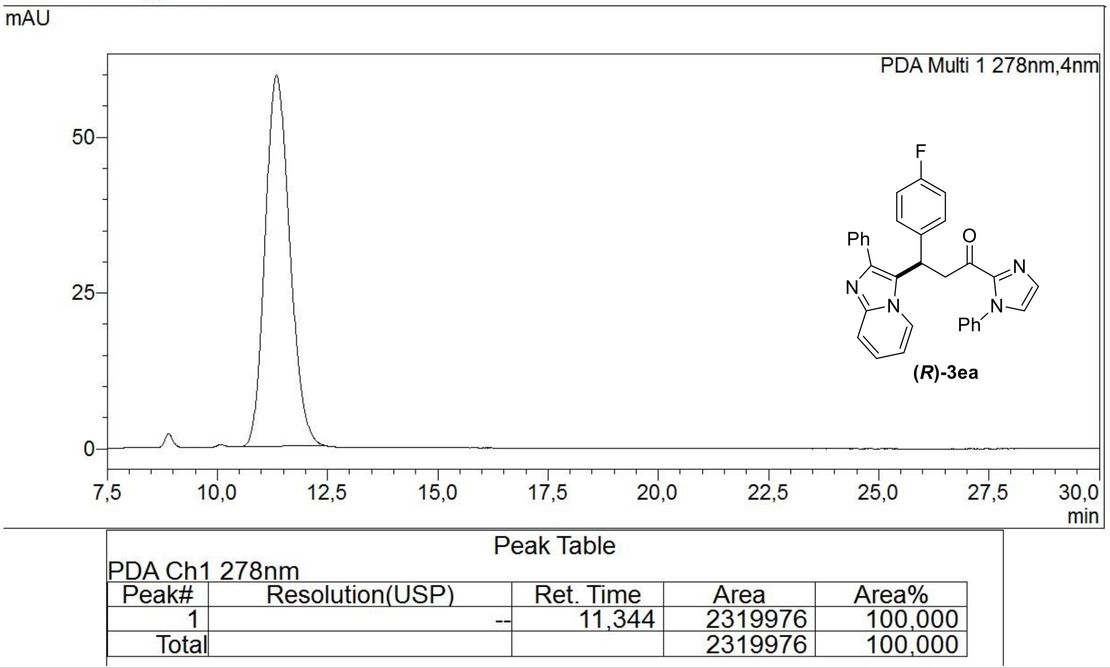

Peak Table

PDA Ch1 278nm

Peak#	Resolution(USP)	Ret. Time	Area	Area%
1	--	11,344	2319976	100,000
Total			2319976	100,000

Figure S56. Chiral chromatogram of the compound **(*R*)-3ea**.

<Chromatogram>

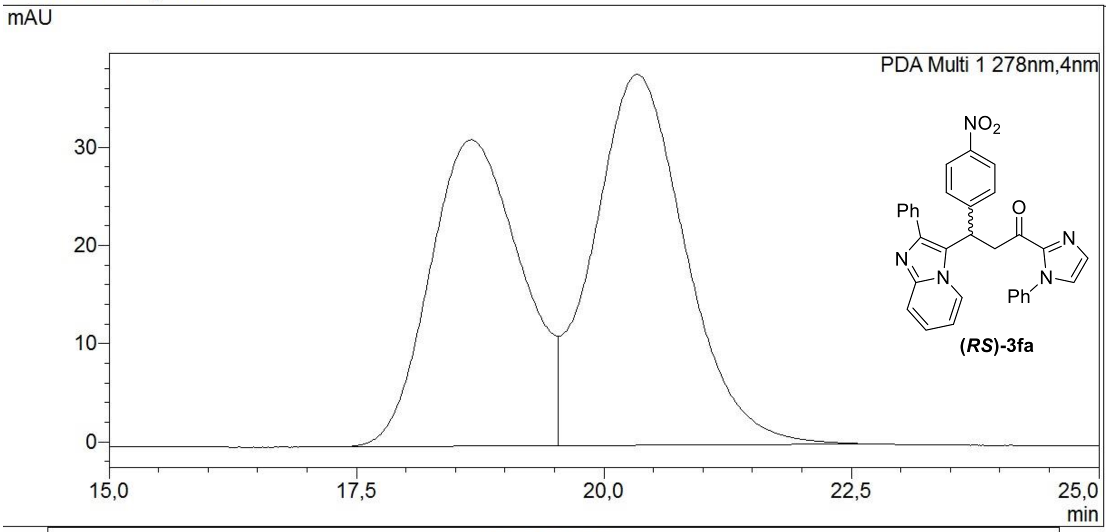

Peak Table

PDA Ch1 278nm

Peak#	Resolution(USP)	Ret. Time	Area	Area%
1	--	18,659	2003117	45,096
2	0,955	20,339	2438757	54,904
Total			4441874	100,000

Figure S57. Racemic chromatogram of the compound **(*RS*)-3fa**.

<Chromatogram>

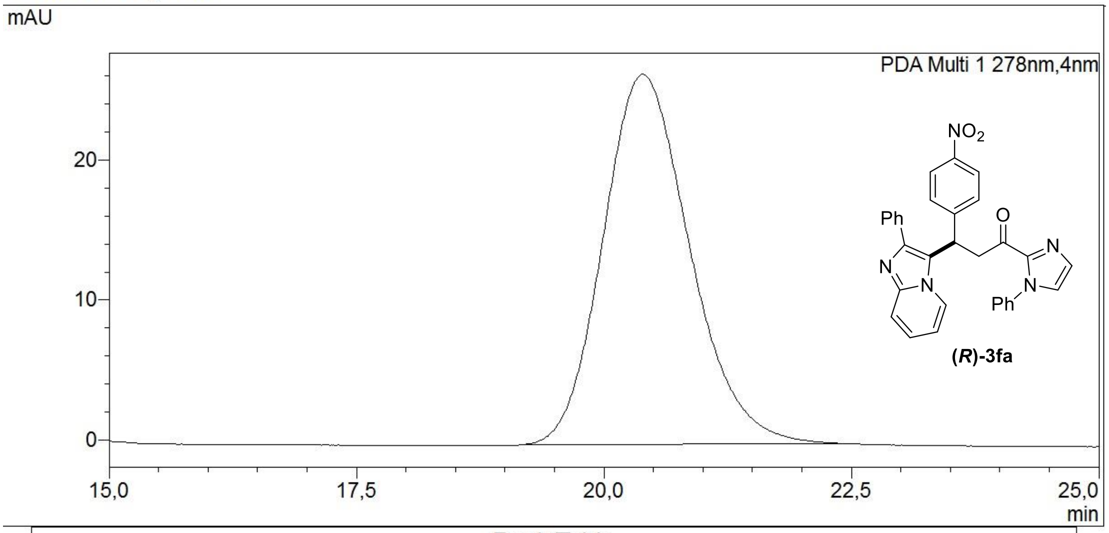

Peak Table

PDA Ch1 278nm

Peak#	Resolution(USP)	Ret. Time	Area	Area%
1	--	20,391	1608037	100,000
Total			1608037	100,000

Figure S58. Chiral chromatogram of the compound **(*R*)-3fa**.

<Chromatogram>

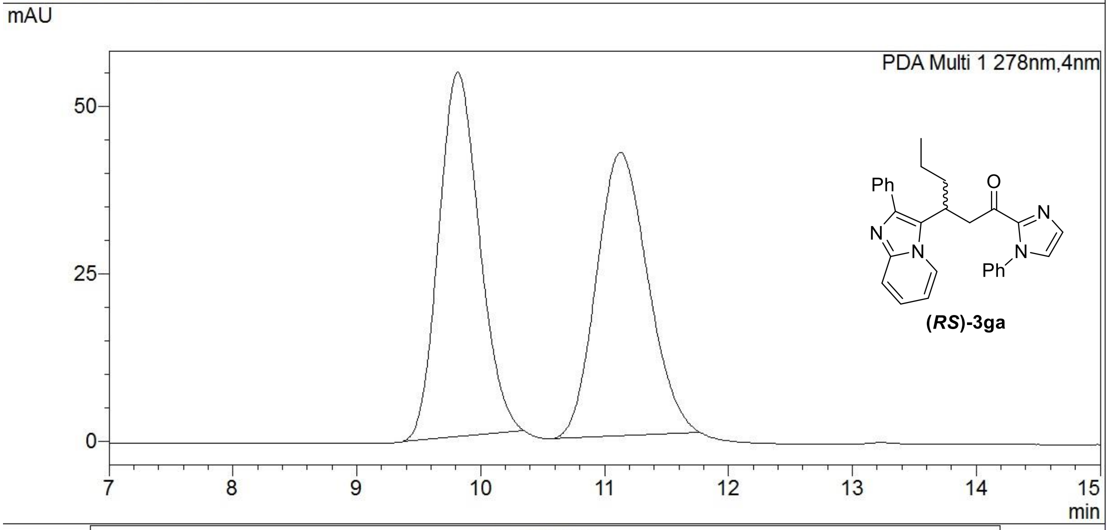

Peak Table

PDA Ch1 278nm

Peak#	Resolution(USP)	Ret. Time	Area	Area%
1	--	9,816	1237778	50,405
2	1,891	11,129	1217882	49,595
Total			2455660	100,000

Figure S59. Racemic chromatogram of the compound **(*RS*)-3ga**.

<Chromatogram>

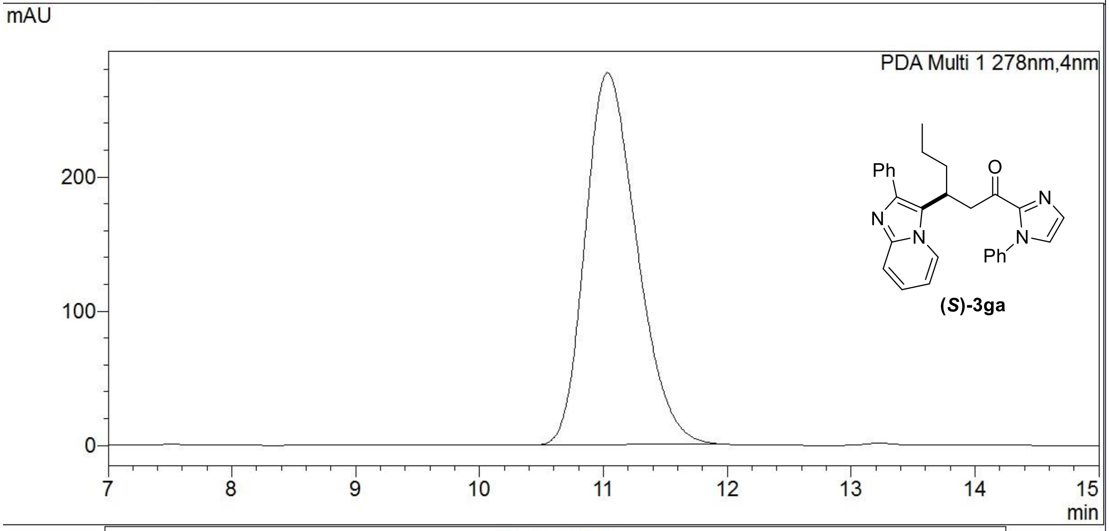

Peak Table

PDA Ch1 278nm

Peak#	Resolution(USP)	Ret. Time	Area	Area%
1	--	11,033	8015906	100,000
Total			8015906	100,000

Figure S60. Chiral chromatogram of the compound **(*S*)-3ga**.

<Chromatogram>

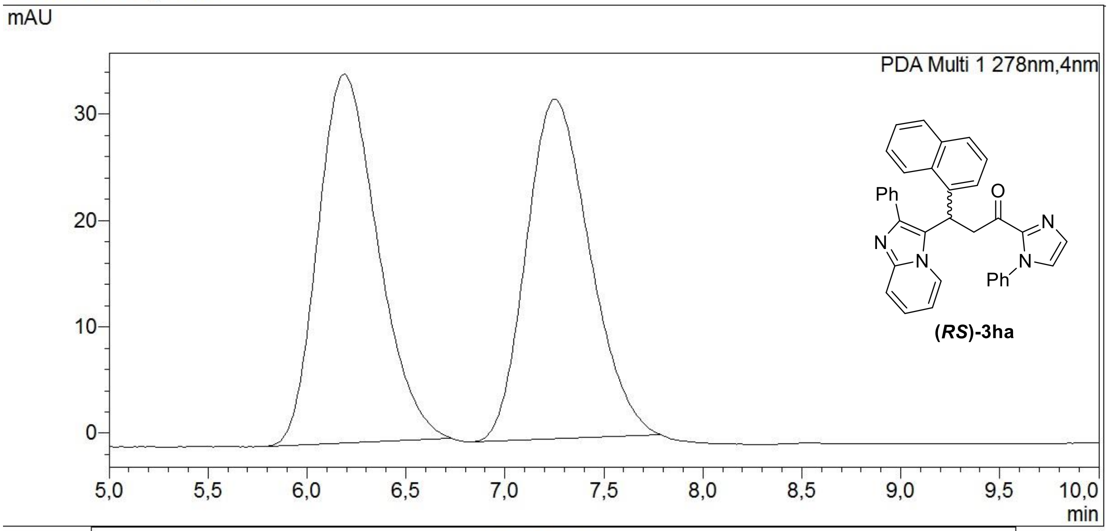

Peak Table

PDA Ch1 278nm

Peak#	Resolution(USP)	Ret. Time	Area	Area%
1	--	6,188	729130	50,518
2	1,825	7,252	714182	49,482
Total			1443312	100,000

Figure S61. Racemic chromatogram of the compound **(*RS*)-3ha**.

<Chromatogram>

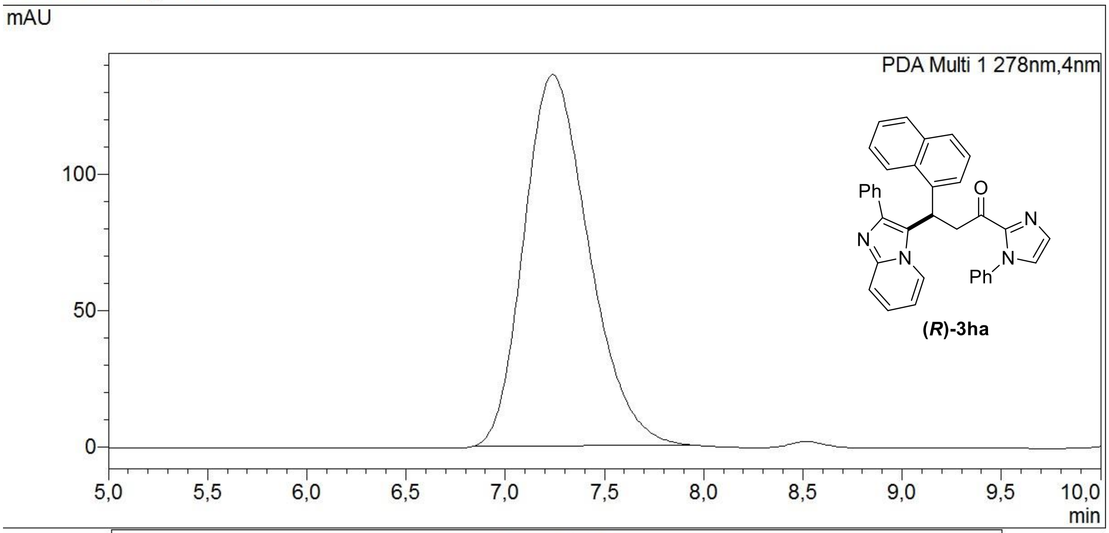

Peak Table

PDA Ch1 278nm

Peak#	Resolution(USP)	Ret. Time	Area	Area%
1	--	7,238	3094317	100,000
Total			3094317	100,000

Figure S62. Chiral chromatogram of the compound **(*R*)-3ha**.

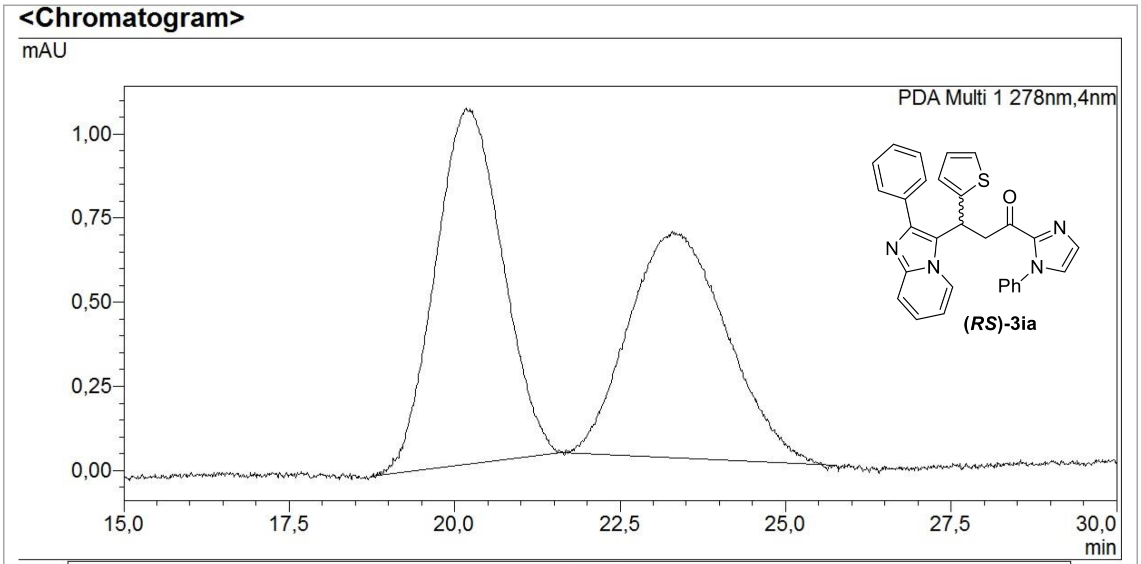

Peak Table

PDA Ch1 278nm

Peak#	Resolution(USP)	Ret. Time	Area	Area%
1	--	20,171	74397	51,770
2	1,335	23,282	69310	48,230
Total			143707	100,000

Figure S63. Racemic chromatogram of the compound **(*RS*)-3ia**.

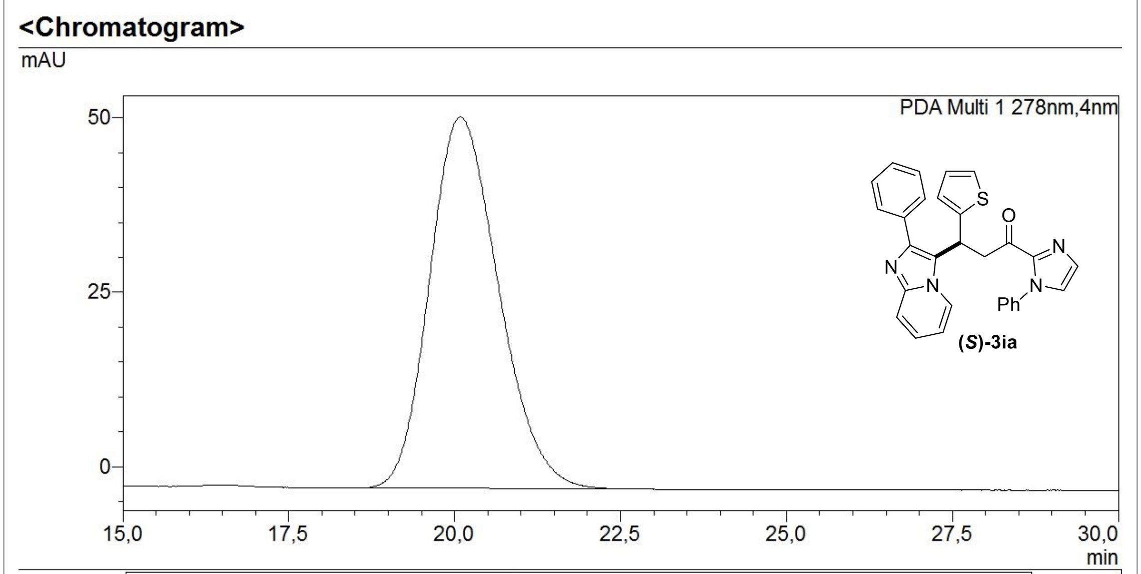

Peak Table

PDA Ch1 278nm

Peak#	Resolution(USP)	Ret. Time	Area	Area%
1	--	20,089	3844400	100,000
Total			3844400	100,000

Figure S64. Chiral chromatogram of the compound **(*S*)-3ia**.

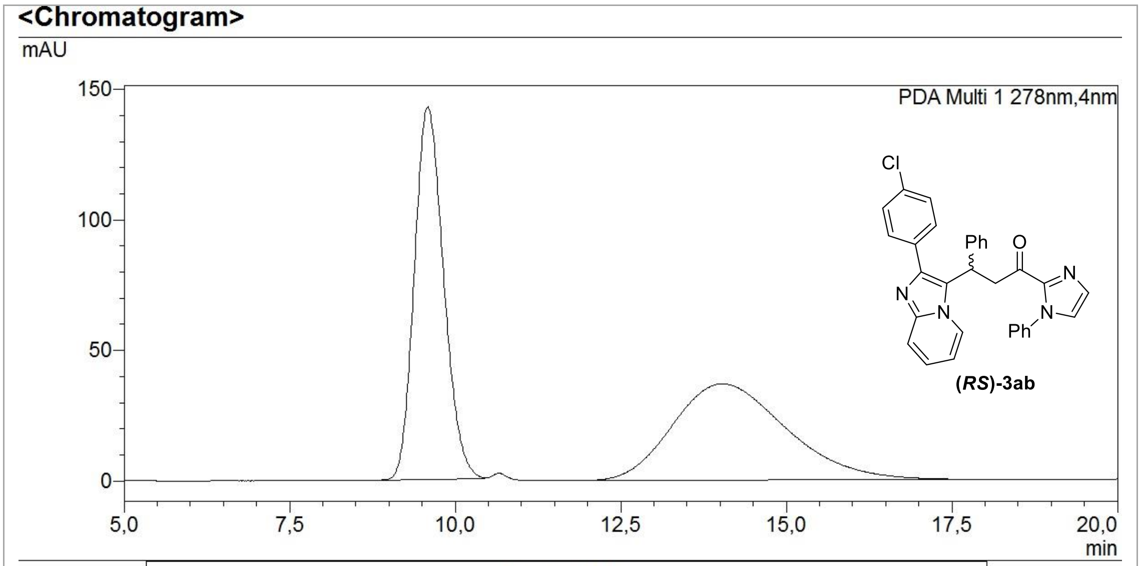

Peak Table

PDA Ch1 278nm

Peak#	Resolution(USP)	Ret. Time	Area
1	--	9,587	4405767
2	2,249	14,046	4382956
Total			8788724

Figure S65. Racemic chromatogram of the compound **(*RS*)-3ab**.

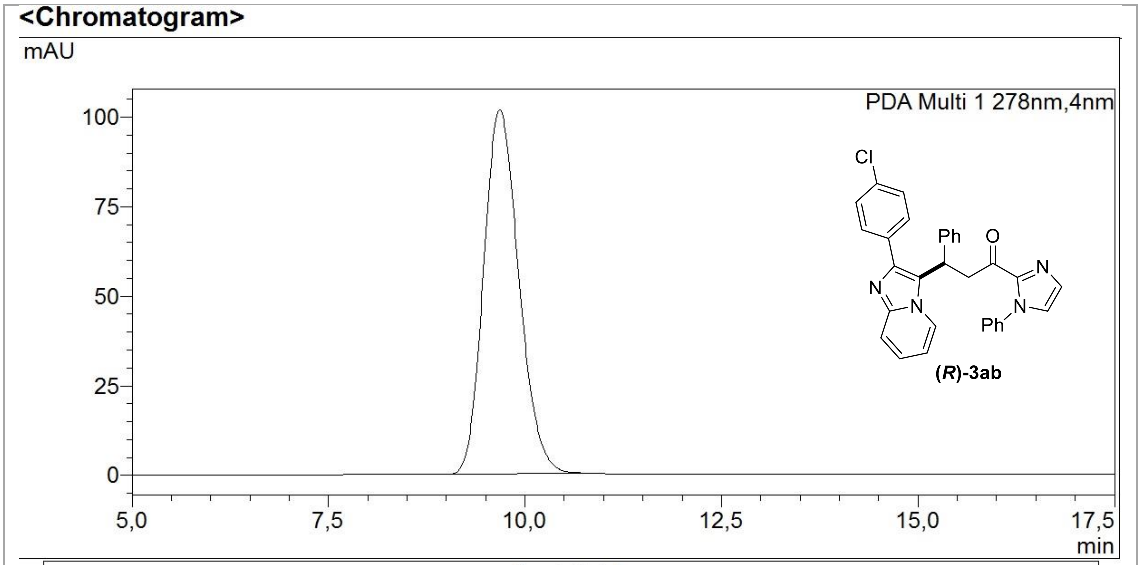

Peak Table

PDA Ch1 278nm

Peak#	Resolution(USP)	Ret. Time	Area	Area%
1	--	9,679	3163878	100,000
Total			3163878	100,000

Figure S66. Chiral chromatogram of the compound **(*R*)-3ab**.

<Chromatogram>

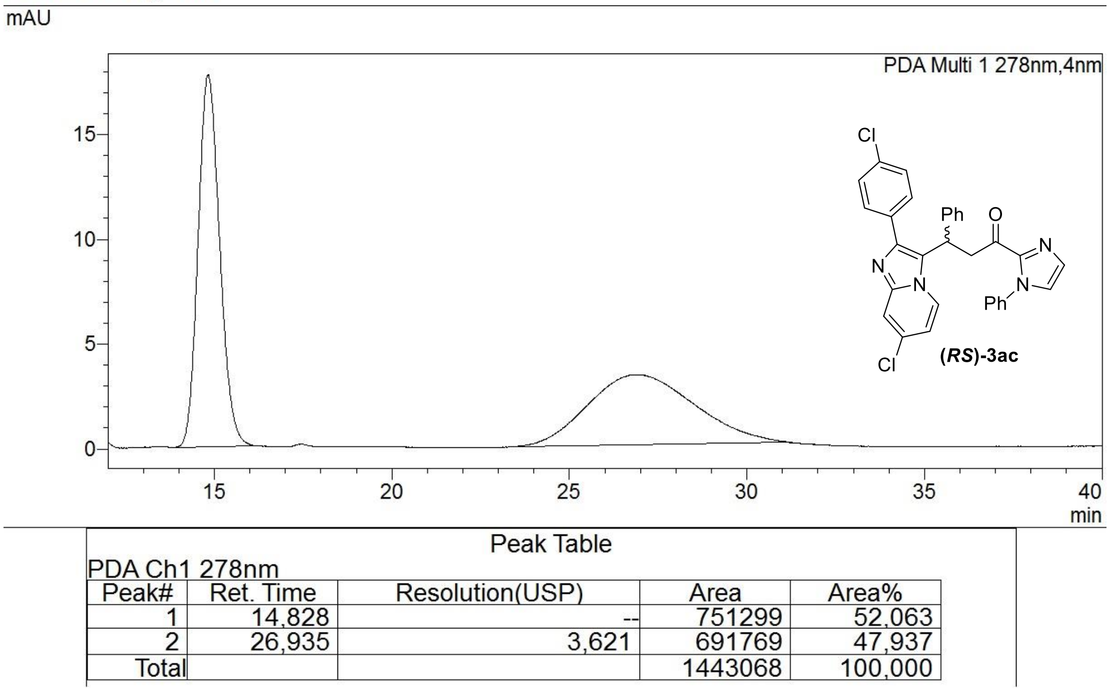

Peak Table

PDA Ch1 278nm

Peak#	Ret. Time	Resolution(USP)	Area	Area%
1	14,828	--	751299	52,063
2	26,935	3,621	691769	47,937
Total			1443068	100,000

Figure S67. Racemic chromatogram of the compound **(*RS*)-3ac**.

<Chromatogram>

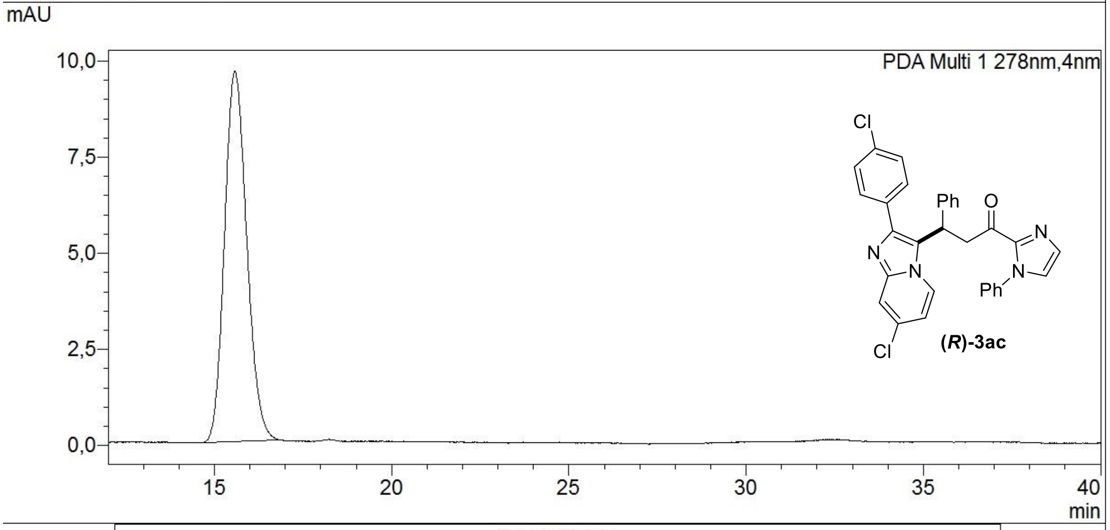

Peak Table

PDA Ch1 278nm

Peak#	Resolution(USP)	Ret. Time	Area	Area%
1	--	15,580	419128	100,000
Total			419128	100,000

Figure S68. Chiral chromatogram of the compound **(*R*)-3ac**.

<Chromatogram>

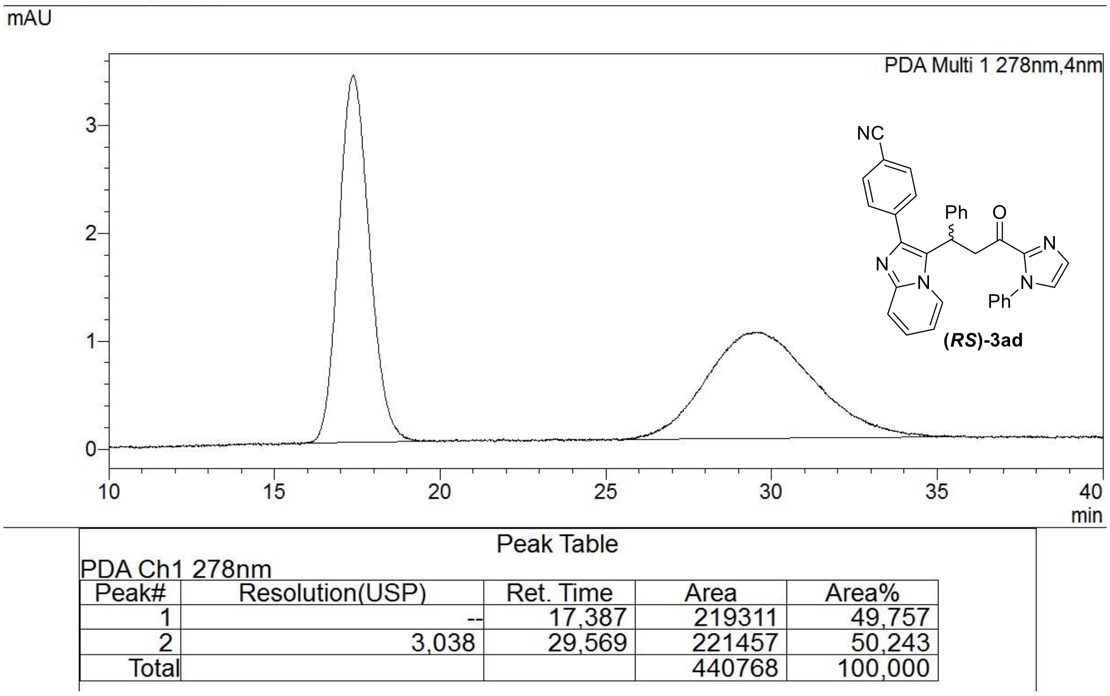

Peak Table

PDA Ch1 278nm

Peak#	Resolution(USP)	Ret. Time	Area	Area%
1	--	17,387	219311	49,757
2	3,038	29,569	221457	50,243
Total			440768	100,000

Figure S69. Racemic chromatogram of the compound **(*RS*)-3ad**.

<Chromatogram>

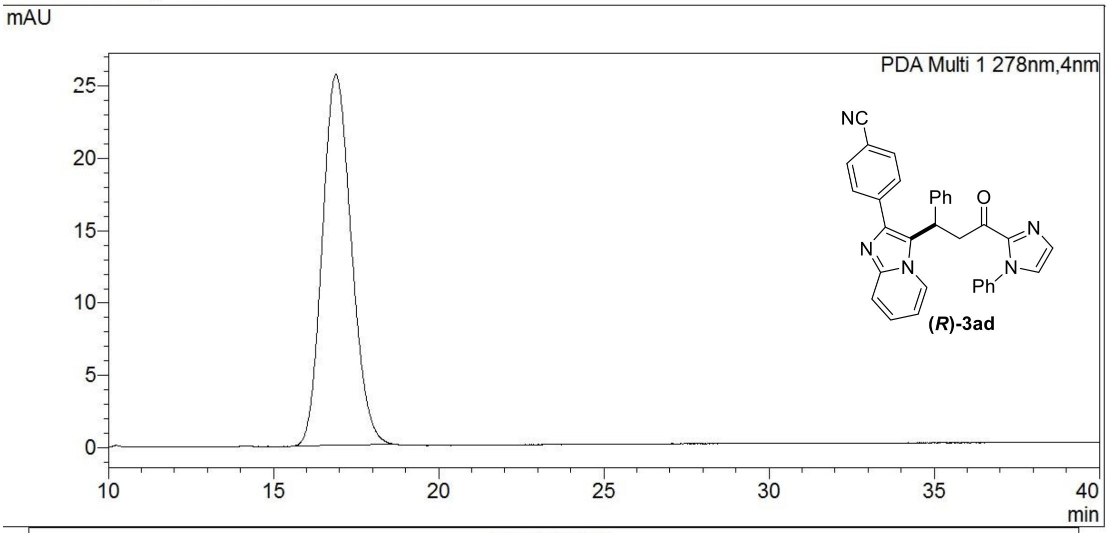

Peak Table

PDA Ch1 278nm

Peak#	Resolution(USP)	Ret. Time	Area	Area%
1	--	16,889	1568742	100,000
Total			1568742	100,000

Figure S70. Chiral chromatogram of the compound **(*R*)-3ad**.

<Chromatogram>

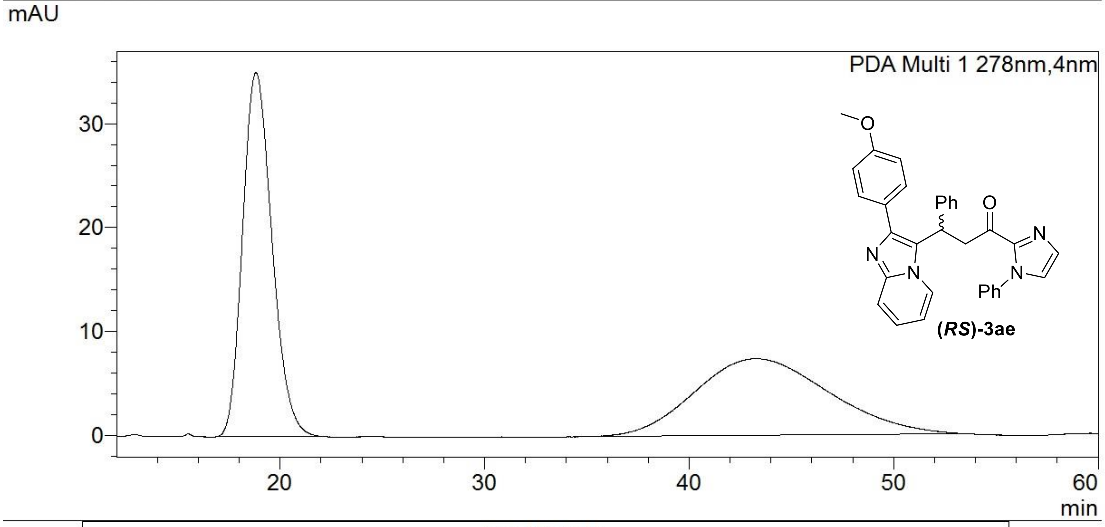

Peak Table

PDA Ch1 278nm

Peak#	Resolution(USP)	Ret. Time	Area	Area%
1	--	18,817	3497875	51,309
2	3,317	43,283	3319358	48,691
Total			6817233	100,000

Figure S71. Racemic chromatogram of the compound **(*RS*)-3ae**.

<Chromatogram>

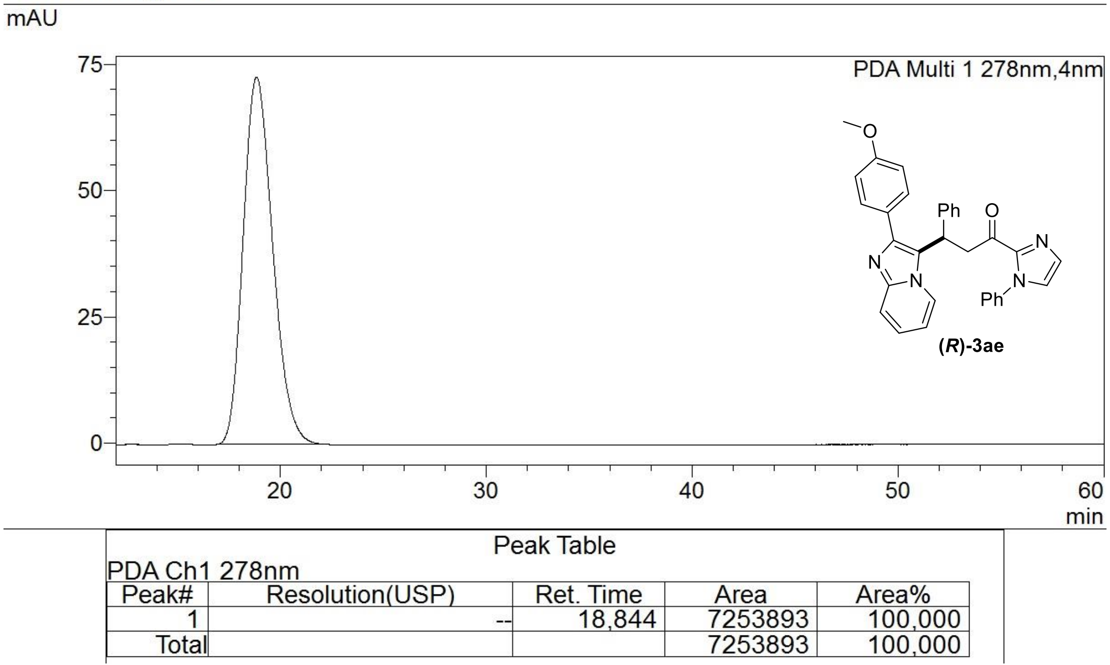

Peak Table

PDA Ch1 278nm

Peak#	Resolution(USP)	Ret. Time	Area	Area%
1	--	18,844	7253893	100,000
Total			7253893	100,000

Figure S72. Chiral chromatogram of the compound **(*R*)-3ae**.

<Chromatogram>

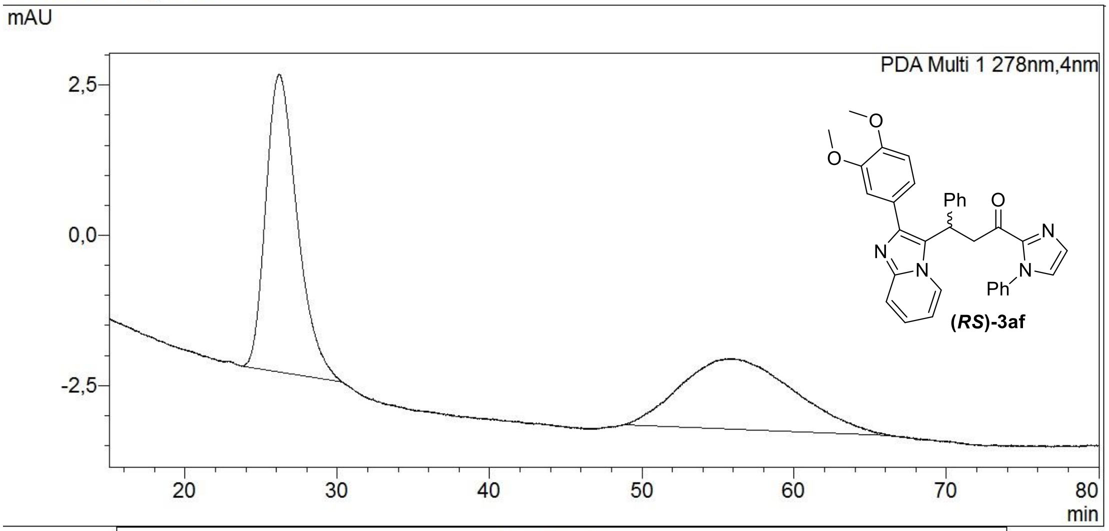

Peak Table

PDA Ch1 278nm

Peak#	Resolution(USP)	Ret. Time	Area	Area%
1	--	26,166	712836	54,075
2	3,938	55,967	605407	45,925
Total			1318243	100,000

Figure S73. Racemic chromatogram of the compound **(*RS*)-3af**.

<Chromatogram>

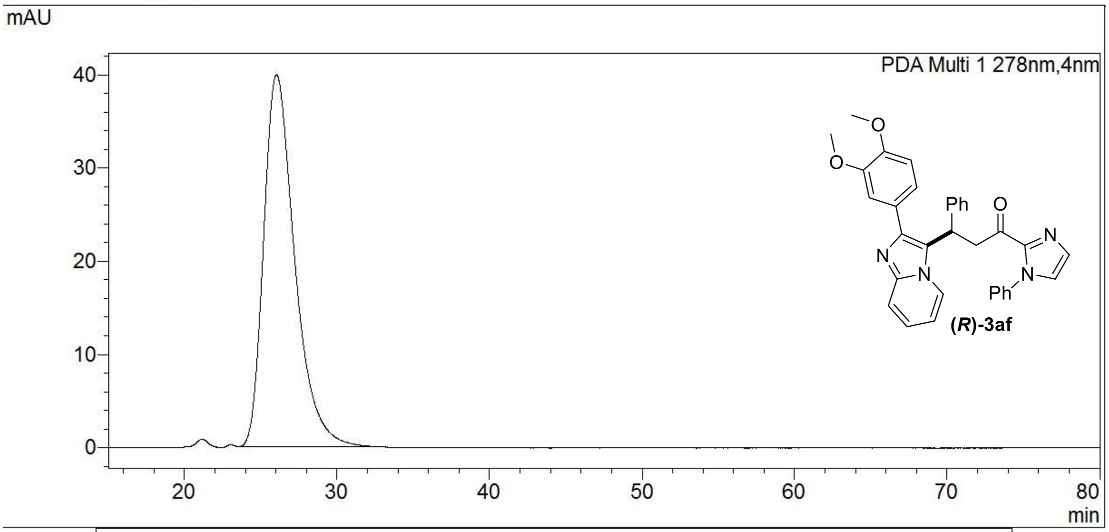

Peak Table

PDA Ch1 278nm

Peak#	Resolution(USP)	Ret. Time	Area	Area%
1	--	26,034	5632204	100,000
Total			5632204	100,000

Figure S74. Chiral chromatogram of the compound **(*R*)-3af**.

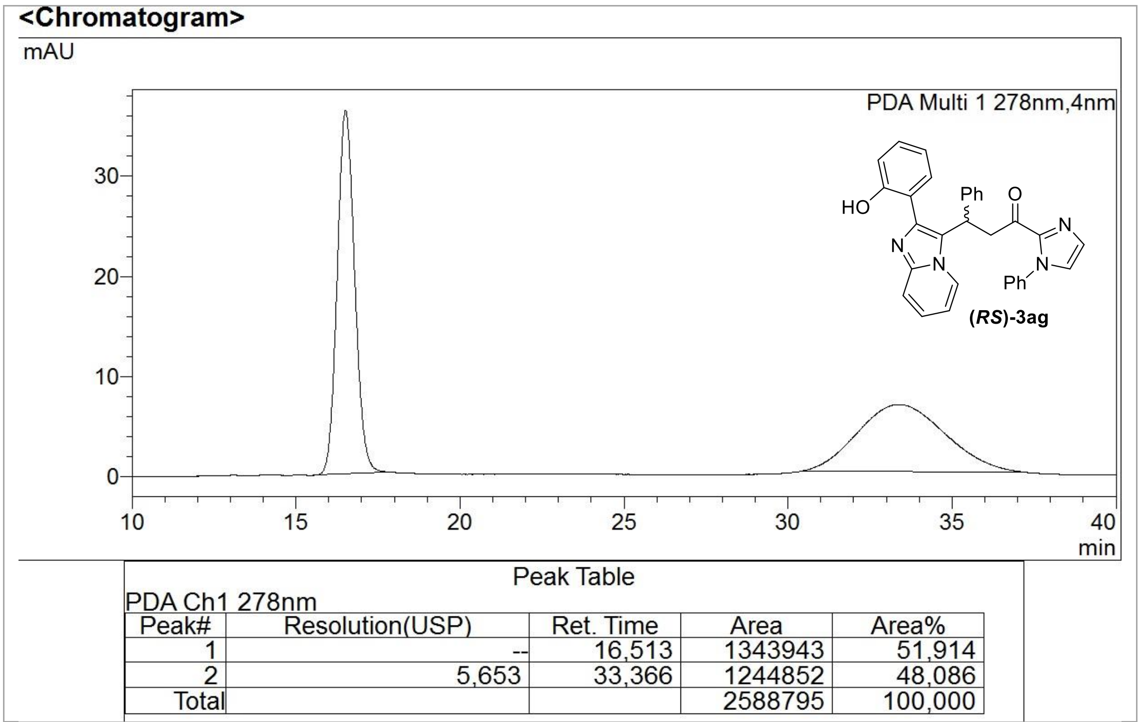

Peak Table

PDA Ch1 278nm

Peak#	Resolution(USP)	Ret. Time	Area	Area%
1	--	16,513	1343943	51,914
2	5,653	33,366	1244852	48,086
Total			2588795	100,000

Figure S75. Racemic chromatogram of the compound **(*RS*)-3ag**.

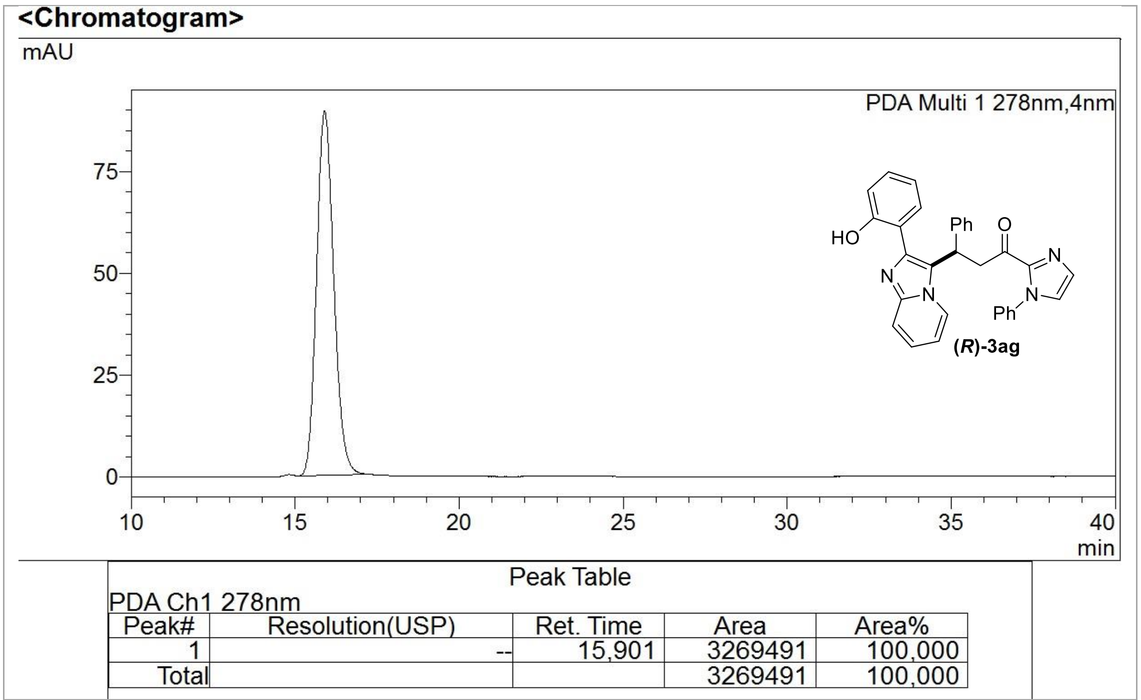

Peak Table

PDA Ch1 278nm

Peak#	Resolution(USP)	Ret. Time	Area	Area%
1	--	15,901	3269491	100,000
Total			3269491	100,000

Figure S76. Chiral chromatogram of the compound **(*R*)-3ag**.

<Chromatogram>

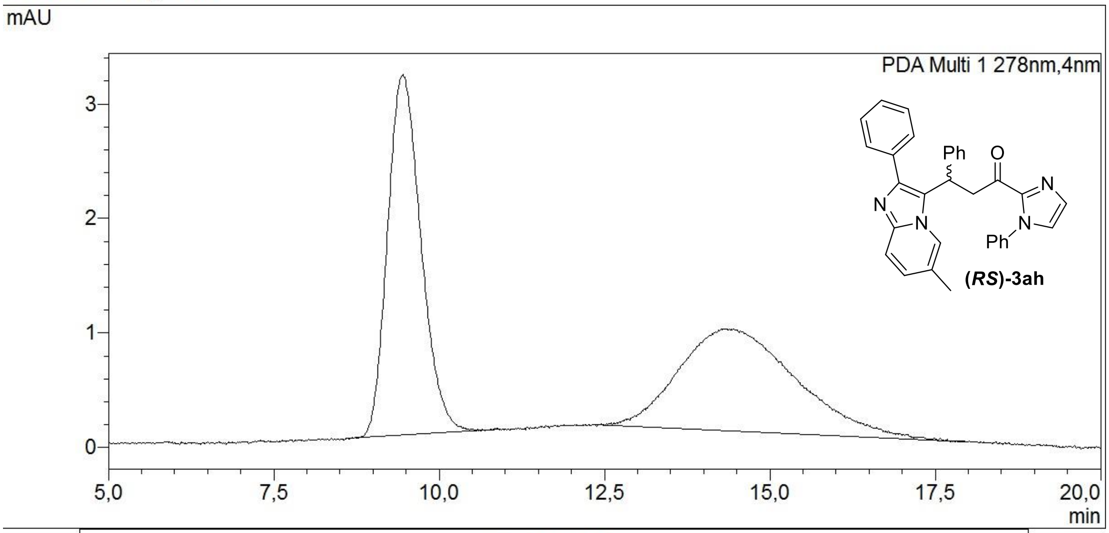

Peak Table

PDA Ch1 278nm

Peak#	Resolution(USP)	Ret. Time	Area	Area%
1	--	9,448	110813	50,327
2	2,394	14,264	109375	49,673
Total			220189	100,000

Figure S77. Racemic chromatogram of the compound **(*RS*)-3ah**.

<Chromatogram>

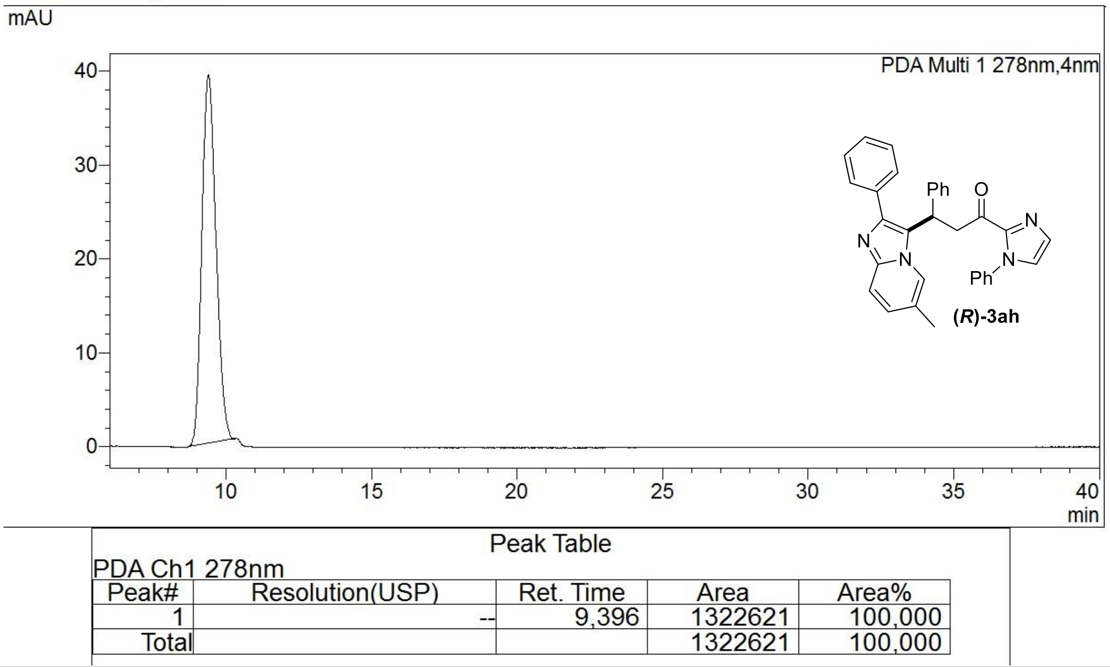

Peak Table

PDA Ch1 278nm

Peak#	Resolution(USP)	Ret. Time	Area	Area%
1	--	9,396	1322621	100,000
Total			1322621	100,000

Figure S78. Chiral chromatogram of the compound **(*R*)-3ah**.

<Chromatogram>

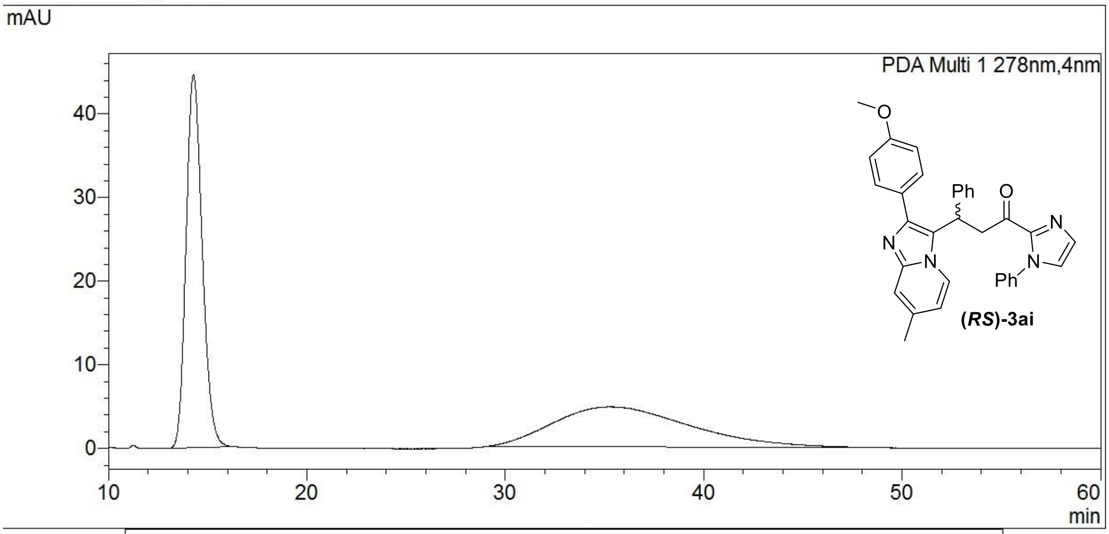

Peak Table

PDA Ch1 278nm

Peak#	Resolution(USP)	Ret. Time	Area	Area%
1	--	14,290	2523101	52,491
2	3,075	35,354	2283660	47,509
Total			4806761	100,000

Figure S79. Racemic chromatogram of the compound **(*RS*)-3ai**.

<Chromatogram>

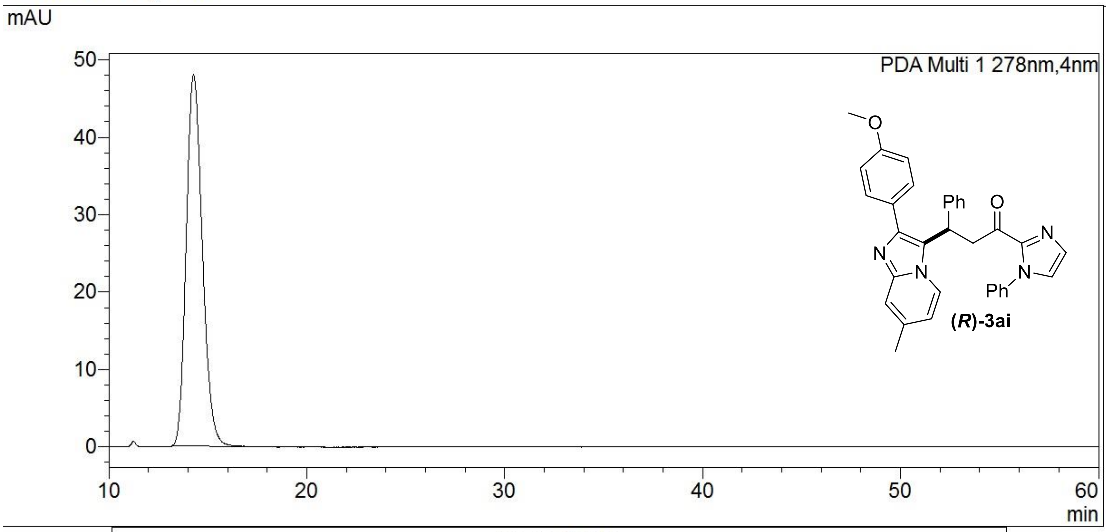

Peak Table

PDA Ch1 278nm

Peak#	Resolution(USP)	Ret. Time	Area	Area%
1	--	14,284	2707714	100,000
Total			2707714	100,000

Figure S80. Chiral chromatogram of the compound **(*R*)-3ai**.

<Chromatogram>

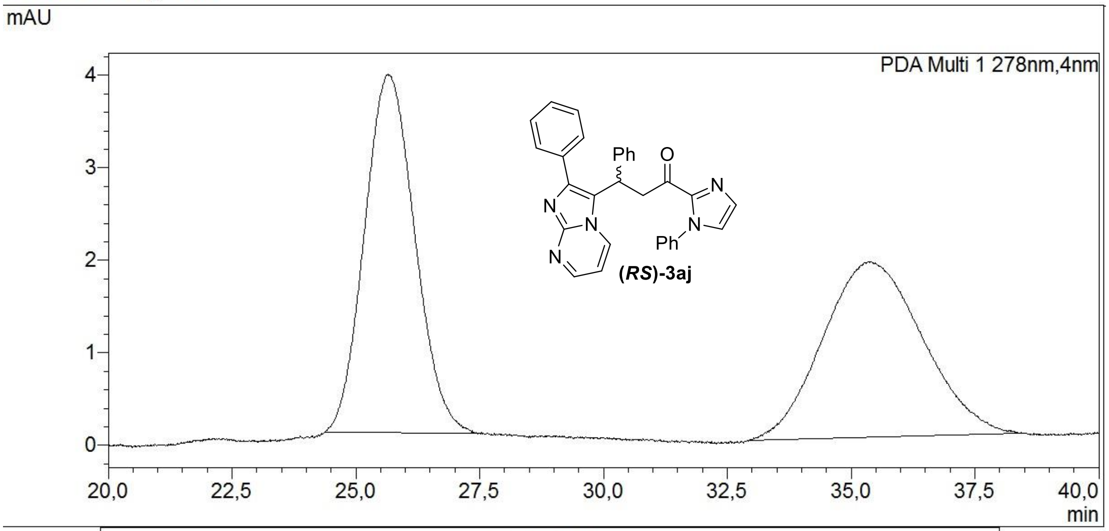

Peak Table

PDA Ch1 278nm

Peak#	Resolution(USP)	Ret. Time	Area	Area%
1	--	25,655	273849	50,752
2	3,494	35,399	265731	49,248
Total			539581	100,000

Figure S81. Racemic chromatogram of the compound **(*RS*)-3aj**.

<Chromatogram>

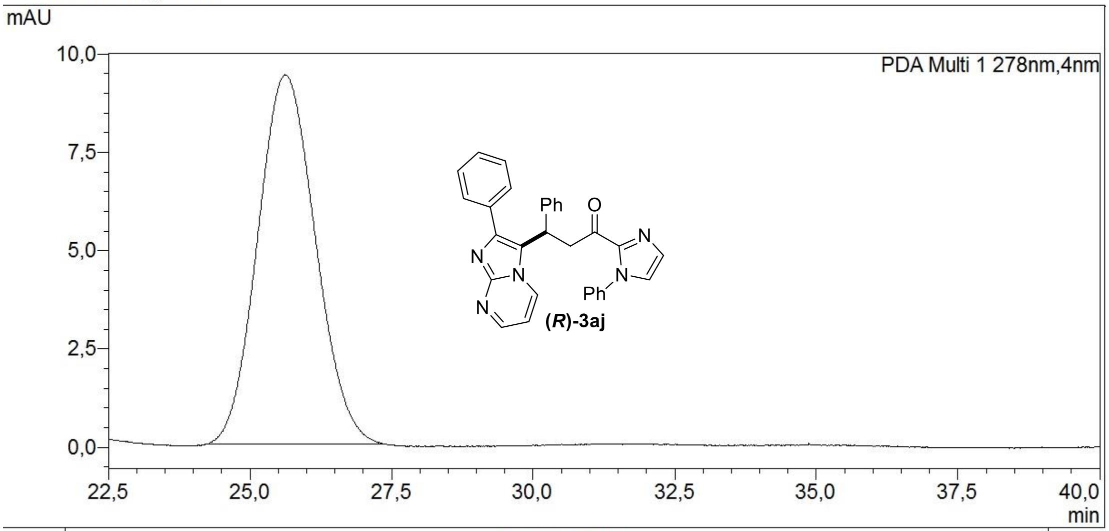

Peak Table

PDA Ch1 278nm

Peak#	Resolution(USP)	Ret. Time	Area	Area%
1	--	25,613	666533	100,000
Total			666533	100,000

Figure S82. Chiral chromatogram of the compound **(*RS*)-3aj**.

<Chromatogram>

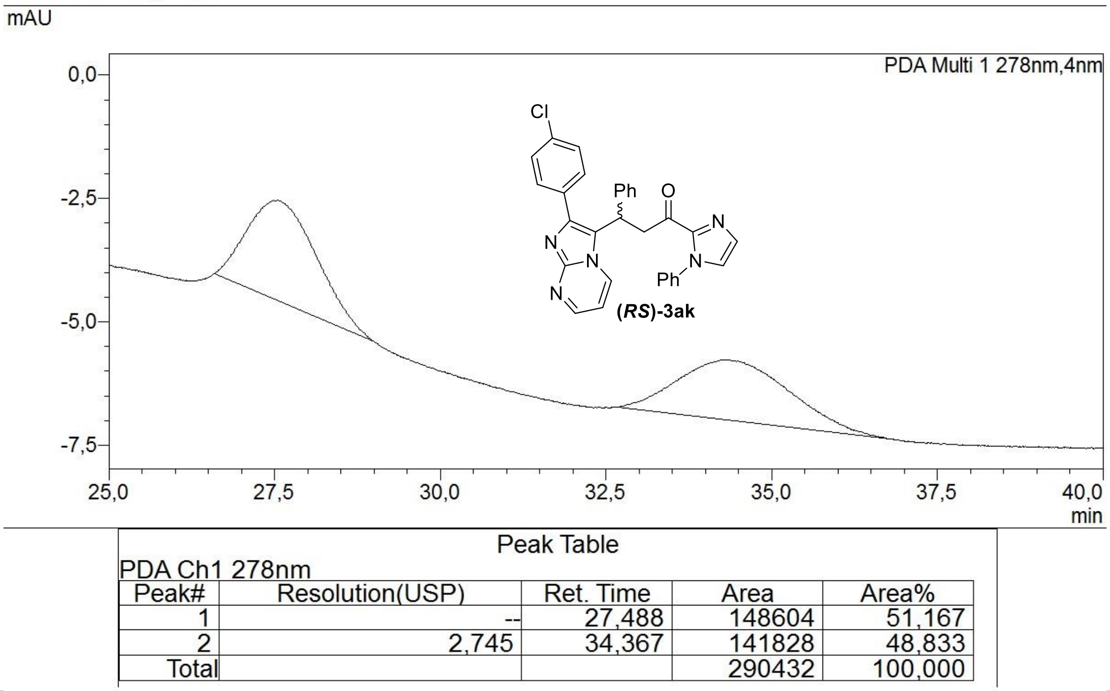

Peak Table

PDA Ch1 278nm

Peak#	Resolution(USP)	Ret. Time	Area	Area%
1	--	27,488	148604	51,167
2	2,745	34,367	141828	48,833
Total			290432	100,000

Figure S83. Racemic chromatogram of the compound **(*RS*)-3ak**.

<Chromatogram>

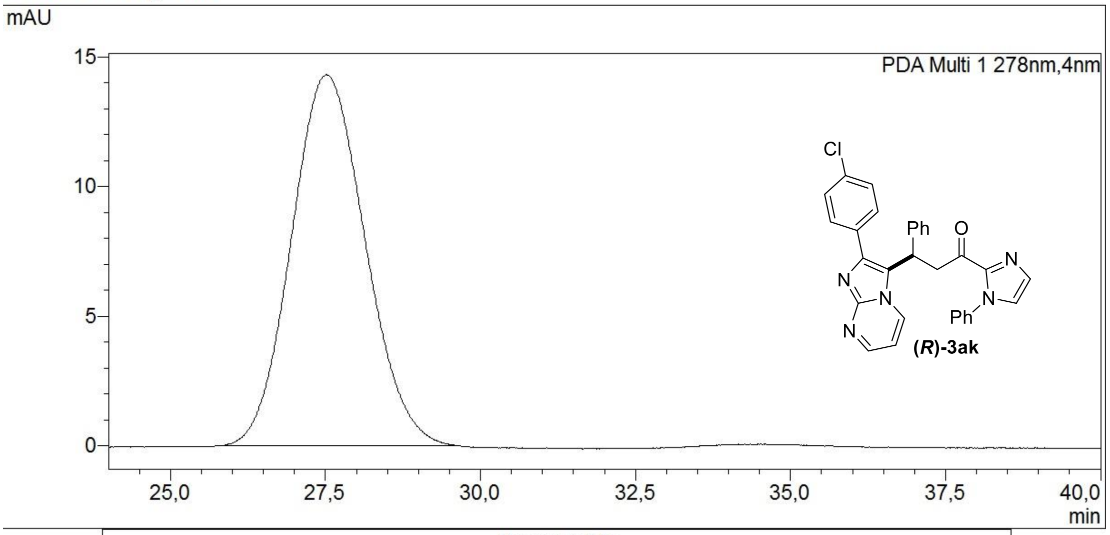

Peak Table

PDA Ch1 278nm

Peak#	Resolution(USP)	Ret. Time	Area	Area%
1	--	27,519	1183137	100,000
Total			1183137	100,000

Figure S84. Chiral chromatogram of the compound **(*R*)-3ak**.

<Chromatogram>

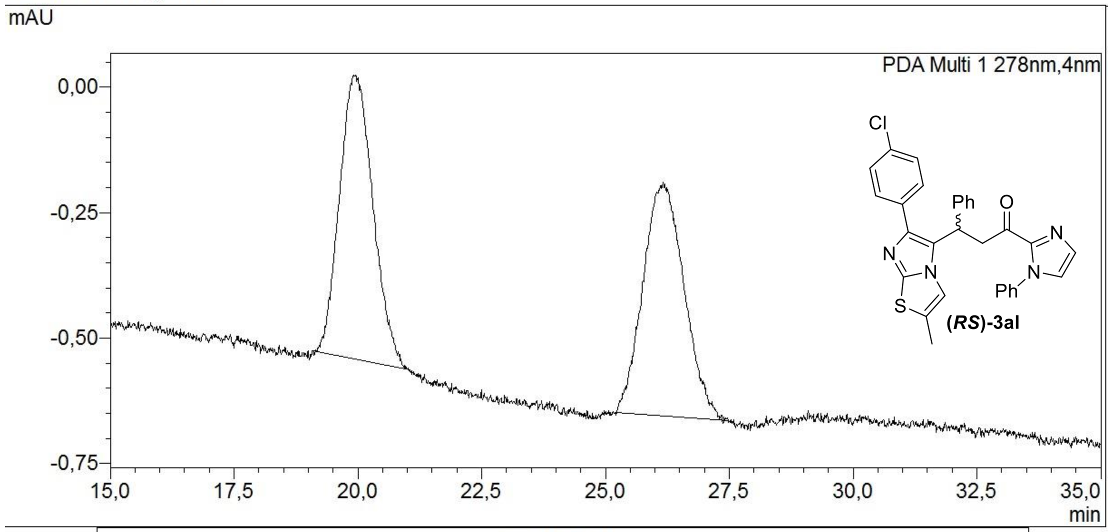

Peak Table

PDA Ch1 278nm

Peak#	Resolution(USP)	Ret. Time	Area	Area%
1	--	19,948	26723	49,980
2	4,611	26,159	26744	50,020
Total			53467	100,000

Figure S85. Racemic chromatogram of the compound **(*RS*)-3al**.

<Chromatogram>

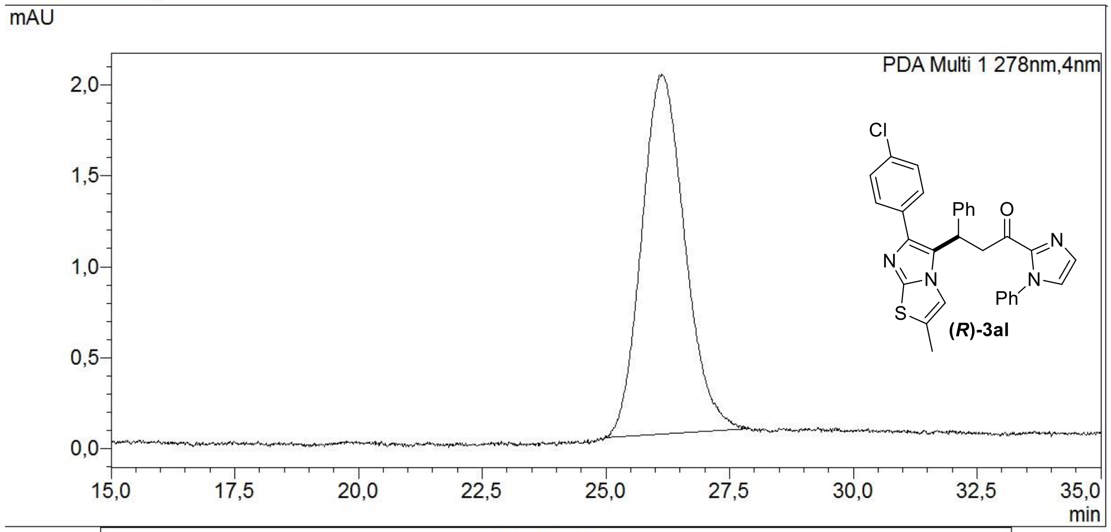

Peak Table

PDA Ch1 278nm

Peak#	Resolution(USP)	Ret. Time	Area	Area%
1	--	26,134	120615	100,000
Total			120615	100,000

Figure S86. Chiral chromatogram of the compound **(*R*)-3al**.

<Chromatogram>

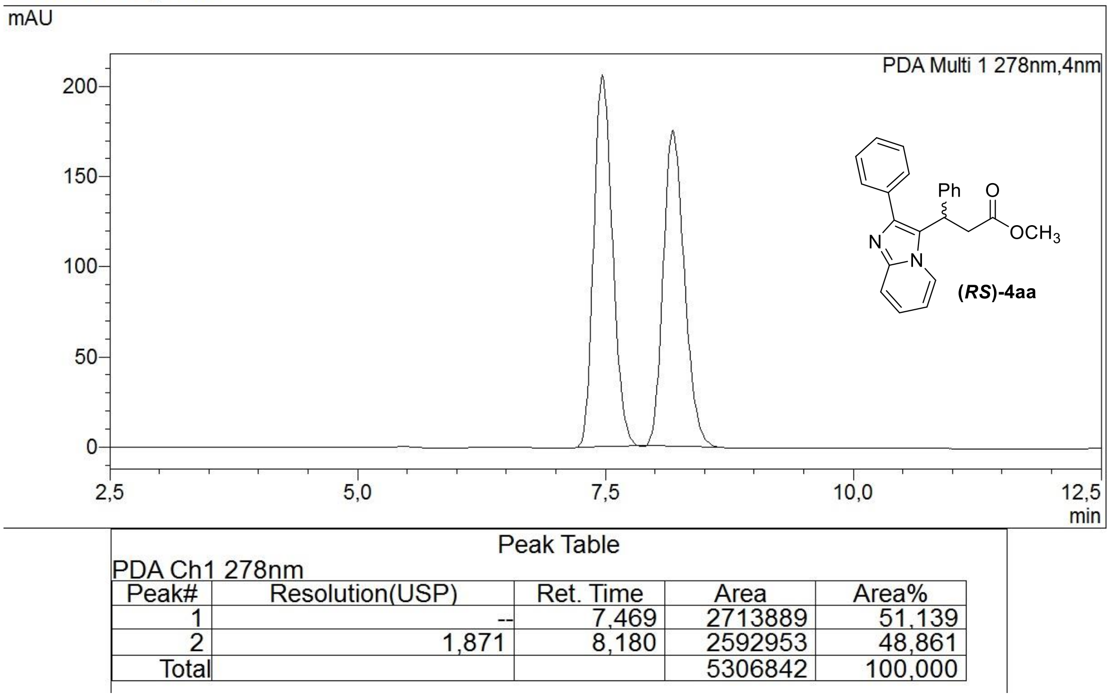

Peak Table

PDA Ch1 278nm

Peak#	Resolution(USP)	Ret. Time	Area	Area%
1	--	7,469	2713889	51,139
2	1,871	8,180	2592953	48,861
Total			5306842	100,000

Figure S87. Racemic chromatogram of the compound **(*RS*)-4aa**.

<Chromatogram>

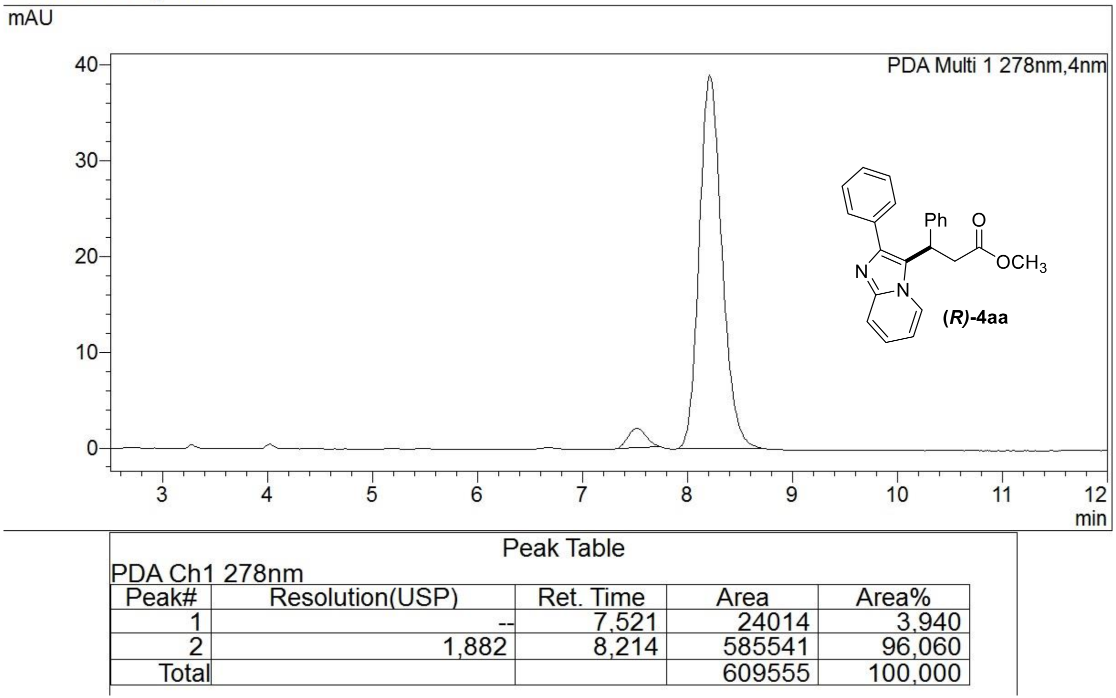

Peak Table

PDA Ch1 278nm

Peak#	Resolution(USP)	Ret. Time	Area	Area%
1	--	7,521	24014	3,940
2	1,882	8,214	585541	96,060
Total			609555	100,000

Figure S88. Chiral chromatogram of the compound **(*R*)-4aa**.

3. Single Crystal X-Ray Diffraction of (*S*)-3ia:

Crystallographic analysis of compound **(*S*)-3ia** was carried out at 150 K on an APEX II Duo diffractometer (Bruker) using graphite monochromated Mo *Kα* radiation (λ = 0.71073 Å). Images were recorded using ϕ and ω scan method and the intensities were integrated with the Bruker SAINT software package.[1] All intensities were corrected for Lorentz, polarization effects and for absorption (SADABS).[2] The structure was solved by dual space method and refined by the full matrix least-squares method using SHELXT [3C] and SHELXL-2019/3,[3] respectively. H atoms attached to C atoms were placed at their idealized positions, with C–H distances and Ueq values taken from the default settings of the refinement program. ORTEP plots and packing pictures were drawn with PLATON program[4] and Mercury,[5] respectively. Full crystallographic tables (including structure factors) for compound **(*S*)-3ia** have been deposited with the Cambridge Crystallographic Data Centre as supplementary publication number CCDC-2313981. Selected crystallographic information are summarized below.

Molecular structure: Compound **(*S*)-3ia** crystallizes in chiral C2 space group and its absolute structure was established by anomalous dispersion effects in diffracted data. The C8 chiral center shows *S* absolute configuration as can be seen in Figure S89.

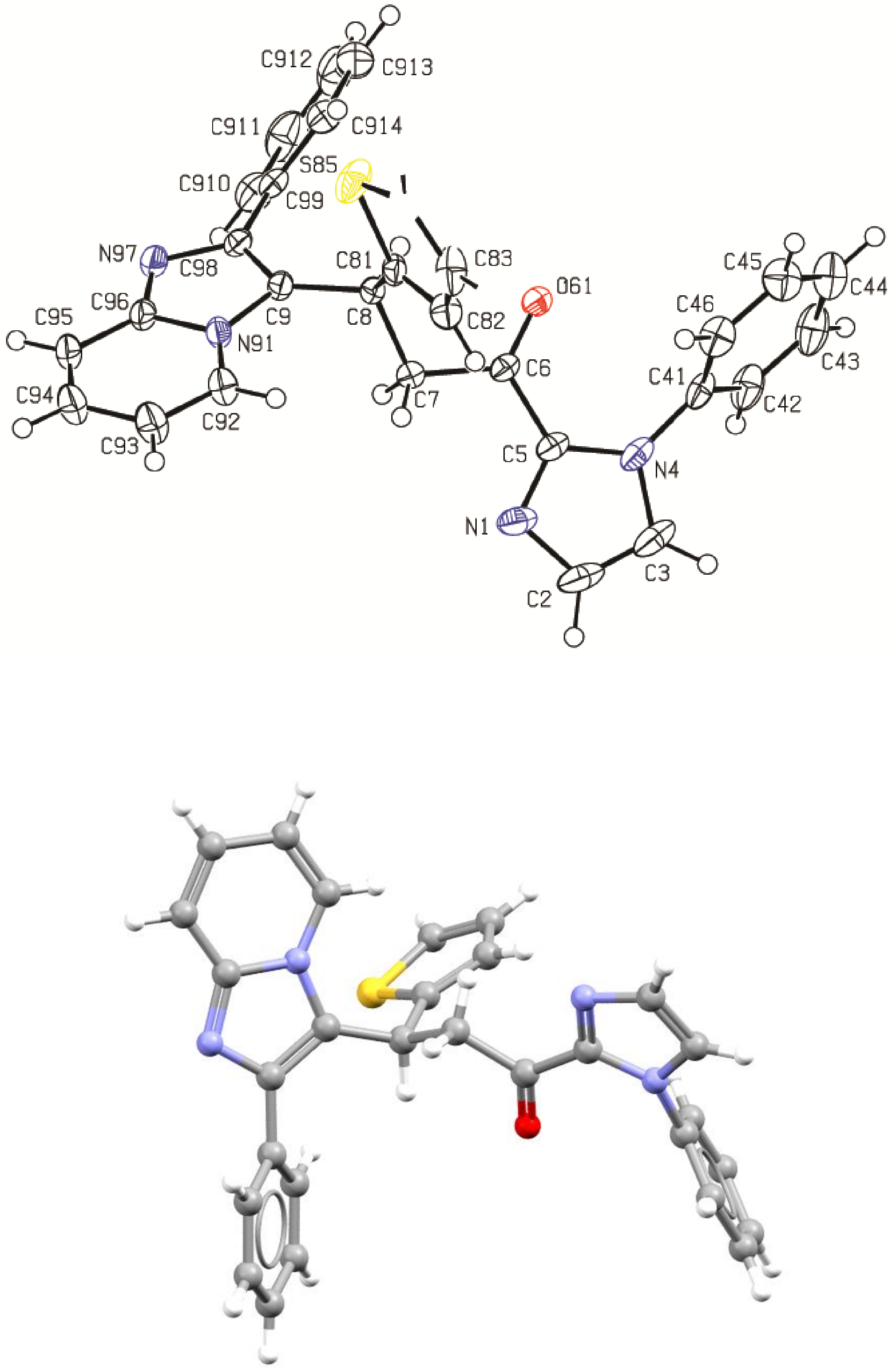

Figure S89. ORTEP plot of **(*S*)-3ia** (top). Elipsoids are drawn at 50% probability level. Molecular structure of **(*S*)-3ia** performed by Mercury (bottom).

Table S1. Crystal data and structure refinement for ***(S)*-3ia**.

Empirical formula	$C_{29.50}H_{23}Cl_{1.50}N_4O_{1.50}S$
Formula weight	542.75
Temperature	150(2) K
Wavelength	0.71073 Å
Crystal system	Monoclinic
Space group	C2
Unit cell dimensions	a = 15.1024(16) Å
	b = 18.2377(19) Å
	c = 11.9404(13) Å
	b= 127.8480(10)°
Volume	2597.0(5) $Å^3$
Z	4
Density (calculated)	1.388 Mg/m^3
Absorption coefficient	0.313 mm^{-1}
F(000)	1126
Crystal size	0./360 x 0.320 x 0.060 mm^3
Theta range for data collection	2.040 to 30.965°.
Index ranges	-21 ≤ h ≤ 21, -26 ≤ k ≤ 26, -17 ≤ l ≤ 17
Reflections collected	25804
Independent reflections	8237 [R(int) = 0.0286]
Completeness to theta = 25.242°	100.0 %
Absorption correction	Semi-empirical from equivalents
Max. and min. transmission	0.7462 and 0.6949
Refinement method	Full-matrix least-squares on F^2
Data / restraints / parameters	8237 / 1 / 344
Goodness-of-fit on F^2	1.031
Final R indices [I>2sigma(I)]	R1 = 0.0481, wR2 = 0.1163
R indices (all data)	R1 = 0.0697, wR2 = 0.1287
Absolute structure parameter	-0.003(15)
Largest diff. peak and hole	0.813 and -0.568 e.$Å^{-3}$

Table S2. Bond lengths [Å] and angles [°] for ***(S)*-3ia**.

N(1)-C(5)
1.327(4)
N(1)-C(2)
1.371(4)
C(2)-C(3)
1.347(6)
C(3)-N(4)
1.374(4)
N(4)-C(5)
1.372(4)
N(4)-C(41)
1.442(4)
C(5)-C(6)
1.476(4)
C(6)-O(61)

1.214(3)
C(6)-C(7)
1.523(4)
C(7)-C(8)
1.543(4)
C(8)-C(9)
1.501(3)
C(8)-C(81)
1.508(4)
C(9)-C(98)
1.384(4)
C(9)-N(91)
1.393(3)
C(41)-C(42)

1.379(4)
C(41)-C(46)

1.393(4)
C(42)-C(43)

1.386(5)
C(43)-C(44)

1.383(6)
C(44)-C(45)

1.391(5)
C(45)-C(46)

1.383(5)
C(81)-C(82)

1.367(4)
C(81)-S(85)

1.726(3)
C(82)-C(83)

1.432(4)
C(83)-C(84)

1.347(6)
C(84)-S(85)

1.717(4)
N(91)-C(92)

1.371(4)
N(91)-C(96)

1.394(4)
C(92)-C(93)

1.362(4)
C(93)-C(94)

1.431(5)
C(94)-C(95)

1.354(5)
C(5)-N(1)-C(2)

105.1(3)
C(3)-C(2)-N(1)

110.9(3)
C(2)-C(3)-N(4)

106.5(3)
C(5)-N(4)-C(3)

106.3(3)
C(5)-N(4)-C(41)

130.1(2)
C(3)-N(4)-C(41)

123.4(3)

C(95)-C(96)

1.418(4)
C(96)-N(97)

1.328(4)
N(97)-C(98)

1.370(4)
C(98)-C(99)

1.473(4)
C(99)-C(910)

1.388(5)
C(99)-C(914)

1.393(5)
C(910)-C(911)

1.392(6)
C(911)-C(912)

1.361(7)
C(912)-C(913)

1.395(7)
C(913)-C(914)

1.392(5)
C(1S)-Cl(1)

1.540(14)
C(1S)-Cl(2)

1.823(8)
C(1S)-Cl(1)^{i}

2.004(13)
C(1S)-H(1S)

1.0000
O(1W)-H(1WD)

0.8587

N(1)-C(5)-N(4)

111.2(3)
N(1)-C(5)-C(6)

123.1(3)
N(4)-C(5)-C(6)

125.3(3)
O(61)-C(6)-C(5)

122.0(3)
O(61)-C(6)-C(7)

122.0(2)
C(5)-C(6)-C(7)

115.9(2)

C(6)-C(7)-C(8)

111.8(2)
C(9)-C(8)-C(81)

113.6(2)
C(9)-C(8)-C(7)

112.0(2)
C(81)-C(8)-C(7)

111.9(2)
C(98)-C(9)-N(91)

104.8(2)
C(98)-C(9)-C(8)

130.2(3)
N(91)-C(9)-C(8)

124.7(2)
C(42)-C(41)-C(46)

120.9(3)
C(42)-C(41)-N(4)

119.3(3)
C(46)-C(41)-N(4)

119.7(3)
C(41)-C(42)-C(43)

119.3(3)
C(44)-C(43)-C(42)

120.8(3)
C(43)-C(44)-C(45)

119.3(4)
C(46)-C(45)-C(44)

120.5(3)
C(45)-C(46)-C(41)

119.2(3)
C(82)-C(81)-C(8)

129.4(2)
C(82)-C(81)-S(85)

110.6(2)
C(8)-C(81)-S(85)

120.0(2)
C(81)-C(82)-C(83)

112.9(3)
C(84)-C(83)-C(82)

112.4(3)
C(83)-C(84)-S(85)

112.1(3)
C(84)-S(85)-C(81)

92.03(17)
C(92)-N(91)-C(9)

131.4(2)
C(92)-N(91)-C(96)

121.9(2)
C(9)-N(91)-C(96)

106.7(2)
C(93)-C(92)-N(91)

119.5(3)
C(92)-C(93)-C(94)

120.3(3)
C(95)-C(94)-C(93)

119.9(3)
C(94)-C(95)-C(96)

120.0(3)
N(97)-C(96)-N(91)

111.3(2)
N(97)-C(96)-C(95)

130.2(3)
N(91)-C(96)-C(95)

118.4(3)
C(96)-N(97)-C(98)

105.4(2)
N(97)-C(98)-C(9)

111.7(3)
N(97)-C(98)-C(99)

120.9(3)
C(9)-C(98)-C(99)

127.4(3)
C(910)-C(99)-C(914)

119.2(3)
C(910)-C(99)-C(98)

119.5(3)
C(914)-C(99)-C(98)

121.3(3)
C(99)-C(910)-C(911)

120.2(4)
C(912)-C(911)-C(910)

120.7(4)
C(911)-C(912)-C(913)

120.0(4)
C(914)-C(913)-C(912)

119.8(4)	
C(913)-C(914)-C(99)	108.0(4)
	Cl(2)-C(1S)-Cl(1)^{i}
120.2(4)	
Cl(1)-C(1S)-Cl(2)	97.9(6)
	H(1WD)-O(1W)-H(1WD)^{i}
118.2(7)	
Cl(1)-C(1S)-Cl(1)^{i}	100.0

Symmetry code: (i) -x+1,y,-z+1

4. References

(1) Bruker (2012). SAINT, Bruker AXS Inc., Madison, Wisconsin, USA.

(2) Bruker (2001). SADABS/, Bruker AXS Inc., Madison, Wisconsin, USA.

(3) Sheldrick, G. M. SHELXT-Integrated Space-Group and Crystal-Structure Determination. *Acta Cryst* **2015***,* A*71*, 3-8.

(4) Spek, A. L. Structure validation in chemical crystallography. *Acta Cryst* **2009**, D*65*, 148-155.

(5) Macrae, C. F.; Edgington, P. R. McCabe, P.; Pidcock, E.; Shields, G. P.; Taylor, R.; Towler, M. & van de Streek, J. Mercury: Visualization and Analysis of Crystal Structures. *J Appl Cryst* **2006**, *39*, 453-457.
